# Supplementary material for: Atomistic modeling of lysophospholipids from the Campylobacter jejuni lipidome
Source: Biophys J. 2025 Aug 28;124(19):3227–43. doi: 10.1016/j.bpj.2025.08.024 (PMC12709257; doi:10.1016/j.bpj.2025.08.024)
Supplement: Document S1. Figures S1–S13 and Tables S1–S12 [file mmc1.pdf]

Biophysical Journal, Volume *124*

Supplemental information

Atomistic modeling of lysophospholipids from the *Campylobacter jejuni* lipidome

Astrid F. Brandner, Kahlan E. Newman, Jonathan W. Essex, and Syma Khalid

Supplemental Material

Atomistic modelling of lysophospholipids from the *Campylobacter jejuni* lipidome

Kahlan E. Newman,[†,&] Astrid F. Brandner,[‡,&] Jonathan W. Essex,[†] and Syma Khalid[†,¶,*]

†*School of Chemistry, University of Southampton, Southampton, UK*

‡*Department of Biochemistry, University of Oxford, Oxford, UK*

¶*Department of Biochemistry, University of Oxford, Oxford, UK*

*E-mail: syma.khalid@bioch.ox.ac.uk

[&]These authors contributed equally

Self-assembly Simulations, Excess Water

Table S1: System contents for self-assembly simulations in excess water

Molecule	Small Single LPL systems				Large Single LPL systems (Anisotropic)			
	$LysoPE_{(18:1)}$	$LysoPE_{(16:0)}$	$LysoPE_{(19:0c)}$	$LysoPG_{(18:1)}$	$LysoPE_{(18:1)}$	$LysoPE_{(16:0)}$	$LysoPE_{(19:0c)}$	$LysoPG_{(18:1)}$
$LysoPE_{(18:1)}$	140	0	0	0	500	0	0	0
$LysoPE_{(16:0)}$	0	140	0	0	0	500	0	0
$LysoPE_{(19:0c)}$	0	0	140	0	0	0	500	0
$LysoPG_{(18:1)}$	0	0	0	140	0	0	0	500
Water	7000	7000	7000	7000	31059	29373	31967	33002
K^+	10	10	10	150	40	40	40	500
CL^-	10	10	10	10	40	40	40	0
Initial box dimensions / nm	7.0 x 7.0 x 7.0				10.9 x 10.9 x 10.9			

Self-assembly Simulations, C_W=0.4

Table S2: System contents for self-assembly simulations at C_W = 0.4

Molecule	Small Single LPL systems				Large Single LPL systems (Anisotropic)			
	$LysoPE_{(18:1)}$	$LysoPE_{(16:0)}$	$LysoPE_{(19:0c)}$	$LysoPG_{(18:1)}$	$LysoPE_{(18:1)}$	$LysoPE_{(16:0)}$	$LysoPE_{(19:0c)}$	$LysoPG_{(18:1)}$
$LysoPE_{(18:1)}$	140	0	0	0	500	0	0	0
$LysoPE_{(16:0)}$	0	140	0	0	0	500	0	0
$LysoPE_{(19:0c)}$	0	0	140	0	0	0	500	0
$LysoPG_{(18:1)}$	0	0	0	140	0	0	0	500
Water	2512	2377	2585	2870	8874	8392	9134	9429
K^+	2	2	2	142	20	20	20	500
CL^-	2	2	2	2	20	20	20	0
Initial box dimensions / nm	7.0 x 7.0 x 7.0				10.9 x 10.9 x 10.9			

Self-assembly Simulations, C_W=0.1

Table S3: System contents for self-assembly simulations at C_W = 0.1

Molecule	Small Single LPL systems				Large Single LPL systems (Anisotropic)			
	LysoPE(18:1)	LysoPE(16:0)	LysoPE(19:0c)	LysoPG(18:1)	LysoPE(18:1)	LysoPE(16:0)	LysoPE(19:0c)	LysoPG(18:1)
**LysoPE(18:1)**	140	0	0	0	500	0	0	0
**LysoPE(16:0)**	0	140	0	0	0	500	0	0
**LysoPE(19:0c)**	0	0	140	0	0	0	500	0
**LysoPG(18:1)**	0	0	0	140	0	0	0	500
**Water**	419	396	431	478	1479	1399	1523	1572
**K^+**	1	1	1	141	5	5	5	500
**CL^-**	1	1	1	1	5	5	5	0
**Initial box dimensions / nm**	5.4 x 5.4 x 5.4				9.9 x 9.9 x 9.9			

Bilayer Self-assembly Simulations

Table S4: System contents for bilayer self-assembly simulations

Molecule	Small Single LPL systems			
	POPG	POPE	POPA	20 % LPL
$LysoPE_{(18:1)}$	0	0	0	7
$LysoPE_{(16:0)}$	0	0	0	7
$LysoPE_{(19:0c)}$	0	0	0	7
$LysoPG_{(18:1)}$	0	0	0	7
POPG	140	0	0	63
POPE	0	140	0	42
POPA	0	0	140	7
Water	7000	7000	7000	7000
K^{+}	10	10	150	87
CL^{-}	10	10	10	10
Initial box dimensions / nm	7.2 x 7.2 x 7.2			

Equilibrium Bilayer Simulations

Table S5: System contents for equilibrium simulation of bilayers with and without lysophospholipids

Molecule	20% LPL			Phospholipids Only		
	R1	R2	R3	R1	R2	R3
$LysoPE_{(18:1)}$	63	63	63	0	0	0
$LysoPE_{(16:0)}$	63	63	63	0	0	0
$LysoPE_{(19:0c)}$	63	63	63	0	0	0
$LysoPG_{(18:1)}$	63	63	63	0	0	0
POPG	567	567	567	828	828	828
POPE	376	376	376	552	552	552
POPA	63	63	63	92	92	92
Water	103808	104583	104663	71713	71691	71756
K^{+}	1145	1147	1147	1113	1114	1113
CL^{-}	452	454	454	193	194	193
Initial box dimensions / nm	7.2 x 7.2 x 7.2			21.1 x 21.1 x 8.9	21.0 x 21.0 x 9.0	21.1 x 21.1 x 8.9

Equilibrium Bilayer Simulation Details

Table S6: Restraints for lipid phosphorus atoms and dihedrals; timestep (dt); and duration for equilibration stages

**Equilibration stage**	**Position restraint** kJ mol $^{-1}$nm^{-2}		**dt** / fs	**Length /** ps
	**Dihedrals**	**Lipid P**		
**NVT1**	1000	1000	1	125
**NVT2**	400	400	1	125
**NPT1**	400	200	2	500
**NPT2**	200	200	2	500
**NPT3**	40	100	2	500
**NPT4**	-	-	2	500

Native protein embedded in Mixed Bilayer

Table S7: System contents for native protein in mixed bilayer. PglB protein was glycosylated in Asn534 (more details in main Method section). Pept refers to the acceptor sequon peptide; LLO refers to the glycosylated lipid donor.

**Molecule**	**20% LPL**<br>**R1-R3**
**LysoPE$_{(18:1)}$**	22
**LysoPE$_{(16:0)}$**	24
**LysoPE$_{(19:0c)}$**	23
**LysoPG$_{(18:1)}$**	24
**POPG**	211
**POPE**	114
**POPA**	21
**LLO**	1
**PglB**	1
**Pept**	1
**MG^{2+}**	2
**Water**	61969
**K^+**	420
**CL^-**	169
**Initial box dimensions / nm**	12.6x12.6x15.8

Electroporation Simulations

Table S8: System contents for electric field simulation of bilayers with and without lysophospholipids

Molecule	20% LPL			Phospholipids Only		
	R1	R2	R3	R1	R2	R3
**$LysoPE_{(18:1)}$**	28	28	28	0	0	0
**$LysoPE_{(16:0)}$**	28	28	28	0	0	0
**$LysoPE_{(19:0c)}$**	28	28	28	0	0	0
**$LysoPG_{(18:1)}$**	28	28	28	0	0	0
**POPG**	252	252	252	324	324	324
**POPE**	168	168	168	216	216	216
**POPA**	28	28	28	36	36	36
**Water**	40150	40122	40194	40320	40323	40323
**K^+**	489	490	492	457	458	458
**CL^-**	181	182	184	97	98	98
**Initial box dimensions / nm**	13.0x13.0x10.9	13.1x13.1x10.8	12.9x12.9x11.1	13.5x13.5x10.4	13.5x13.5x10.4	13.5x13.5x10.5

Simulation box collapse

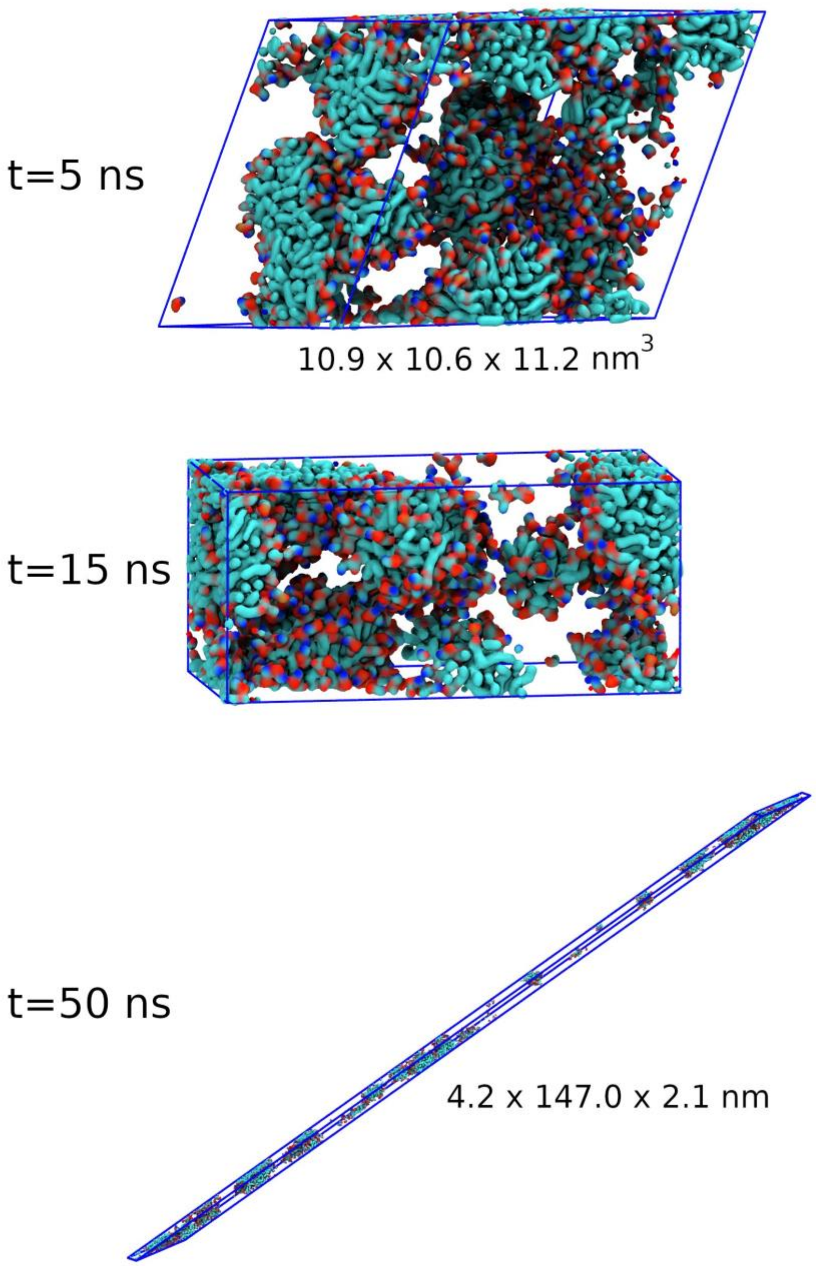

Figure S1: Collapse of a simulation box under anisotropic pressure coupling. Lipids shown as surfaces, coloured by element. Unit cell shown by blue lines. Simulation of lysoPE$_{(18:1)}$ (500 lipids, C_W=0.7) initially forms micelles. The unit cell deforms as the simulation progresses, resulting in box dimensions less than twice the electrostatics cut-off. The simulation crashes shortly after 50 ns.

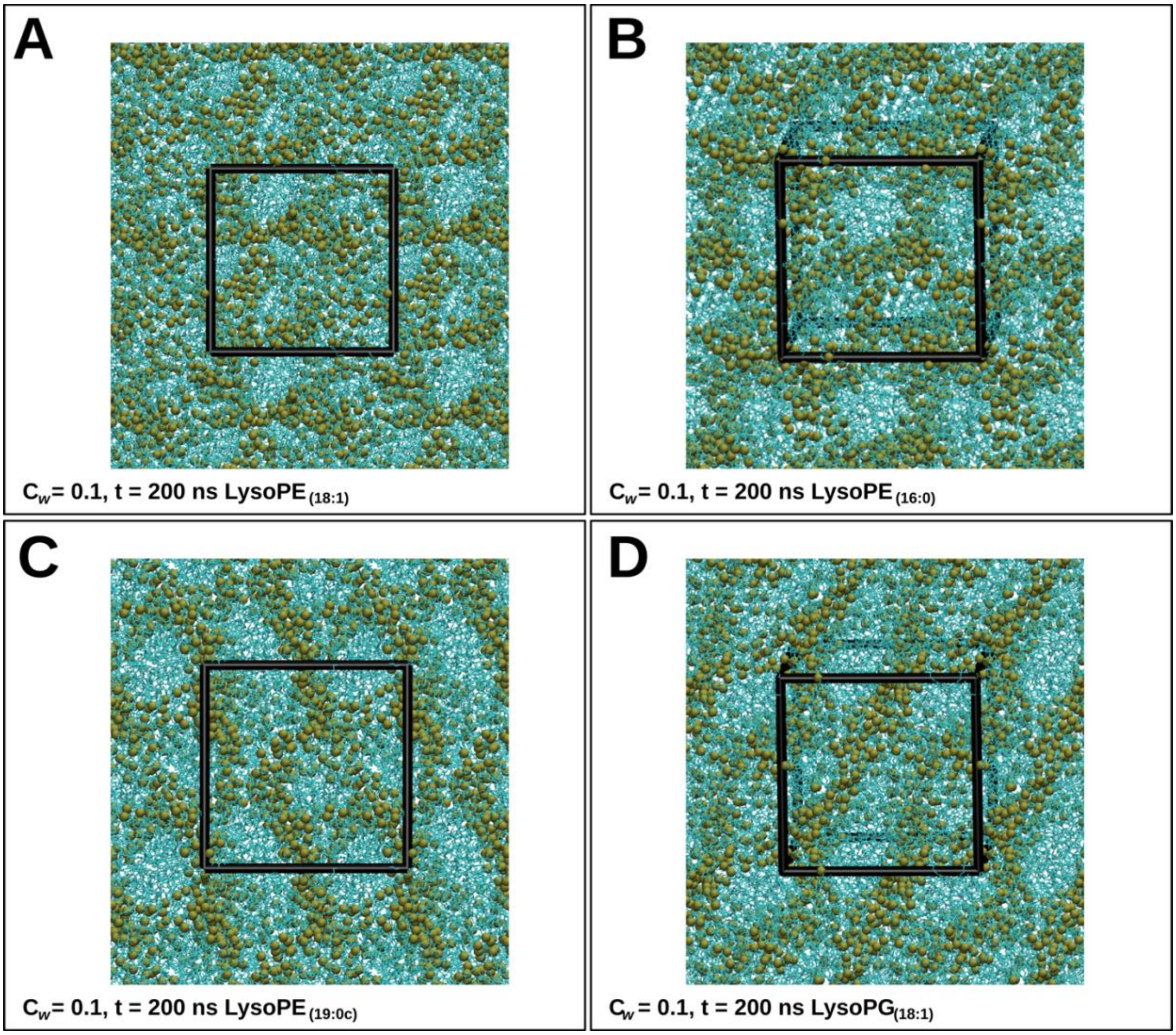

Figure S2: Final snapshots of the isotropic single LPL self-assembly of larger boxes at Cw= 0.1 (most concentrated systems). **(A)** LysoPE $_{(18:1)}$, **(B)** LysoPE $_{(16:0)}$, **(C)** LysoPE, **(D)** LysoPG(18:1). The simulated box is shown in black, and replicated in space to aid the visualisation of the aggregates.

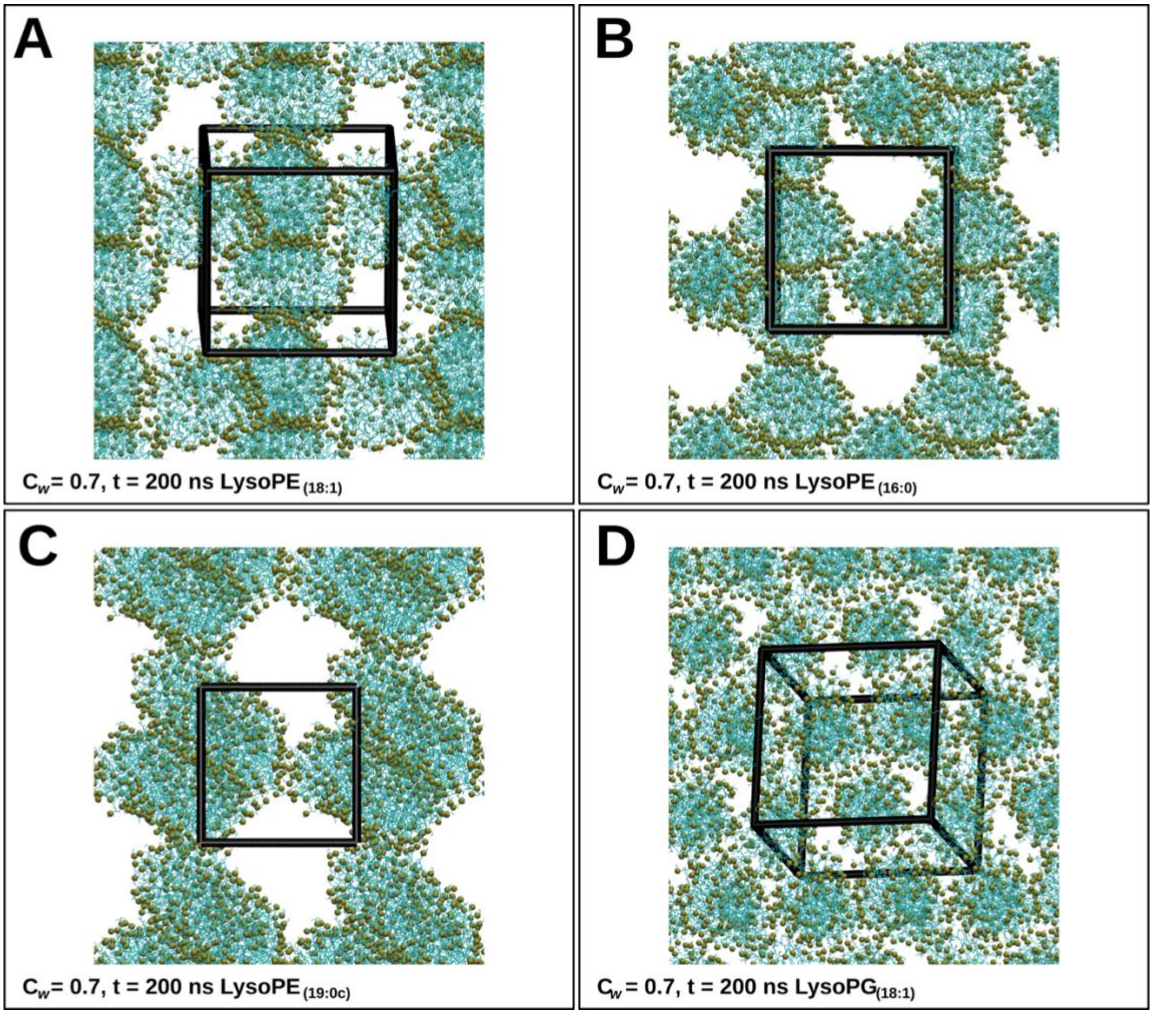

Figure S3: Final snapshots of the isotropic single LPL self-assembly of larger boxes at Cw= 0.7 (most diluted systems). **(A)** LysoPE$_{(18:1)}$, **(B)** LysoPE$_{(16:0)}$, **(C)** LysoPE$_{(19:0c)}$, **(D)** LysoPG(18:1). The simulated box is shown in black, and replicated in space to aid the visualisation of the aggregates.

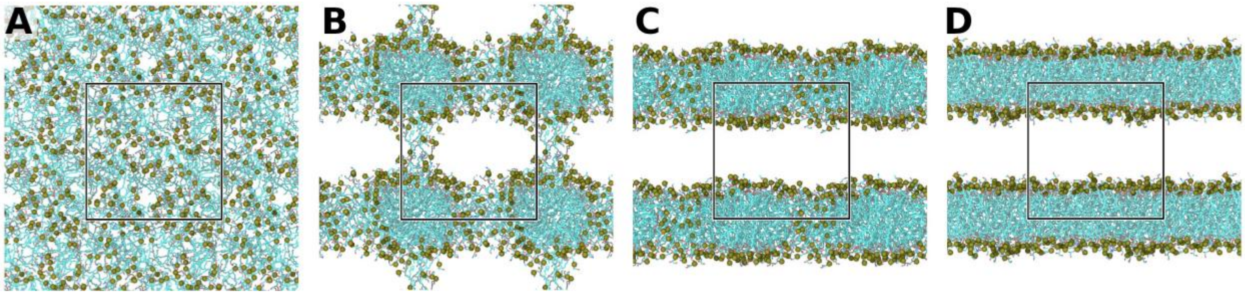

Figure S4: Stages of bilayer self-assembly as described by Skjevik et al. [71]. Snapshots taken from self-assembly simulation of 20% LPL-phospholipid mixture. Lipids shown in stick representation coloured by element. Phosphorus atoms of the lipid headgroups highlighted as tan spheres. Water and ions omitted for clarity. Unit cell indicated by a black box. **(A)** Initial lipid solution. **(B)** Micelle-like assembly with lipid bridges between micelles and their periodic images. **(C)** Porated bilayer with lipid headgroups within the hydrophobic core region. **(D)** Non-porous bilayer formed.

Depletion-enrichment indices

Table S9: Depletion-enrichment indices (DEI) for lipids in the mixed bilayers. Values are presented as the mean ± standard deviation over the second half (final 500 ns) of each simulation. Calculated using the Python package LiPyphilic[41].

Replica	Molecule	DEI						
		**POPG**	**POPE**	**POPA**	**lysoPE**$_{(18:1)}$	**lysoPE**$_{(16:0)}$	**lysoPE**$_{(19:0c)}$	**lysoPG**$_{(18:1)}$
**R1**	**POPG**	0.95 ± 0.02	1.00 ± 0.02	0.92 ± 0.05	0.96 ± 0.05	1.06 ± 0.04	0.97 ± 0.06	0.95 ± 0.05
	**POPE**	1.05 ± 0.02	0.97 ± 0.04	1.11 ± 0.09	1.02 ± 0.08	0.95 ± 0.07	1.02 ± 0.07	1.10 ± 0.06
	**POPA**	0.96 ± 0.05	1.11 ± 0.10	0.9 ± 0.26	1.17 ± 0.28	0.90 ± 0.21	1.17 ± 0.22	0.93 ± 0.21
	**lysoPE**$_{(18:1)}$	1.02 ± 0.06	1.03 ± 0.09	1.19 ± 0.28	0.92 ± 0.21	1.18 ± 0.19	0.98 ± 0.19	1.11 ± 0.20
	**lysoPE**$_{(16:0)}$	1.12 ± 0.05	0.95 ± 0.07	0.90 ± 0.21	1.16 ± 0.20	0.8 ± 0.26	0.97 ± 0.22	0.93 ± 0.20
	**lysoPE**$_{(19:0c)}$	1.04 ± 0.06	1.04 ± 0.08	1.20 ± 0.23	0.99 ± 0.19	0.99 ± 0.23	1.09 ± 0.31	0.99 ± 0.17
	**lysoPG**$_{(18:1)}$	0.95 ± 0.05	1.04 ± 0.07	0.88 ± 0.20	1.04 ± 0.19	0.89 ± 0.20	0.93 ± 0.17	0.93 ± 0.28
**R2**	**POPG**	0.96 ± 0.02	0.99 ± 0.02	0.9 ± 0.05	0.98 ± 0.05	1.03 ± 0.06	0.97 ± 0.06	0.92 ± 0.05
	**POPE**	1.05 ± 0.02	0.98 ± 0.04	1.11 ± 0.06	1.01 ± 0.06	0.98 ± 0.08	1.00 ± 0.06	1.10 ± 0.07
	**POPA**	0.95 ± 0.05	1.11 ± 0.07	1.07 ± 0.39	1.08 ± 0.19	1.16 ± 0.25	1.07 ± 0.19	0.84 ± 0.24
	**lysoPE**$_{(18:1)}$	1.04 ± 0.05	1.02 ± 0.07	1.09 ± 0.187	0.83 ± 0.2	0.97 ± 0.21	1.15 ± 0.19	1.16 ± 0.26
	**lysoPE**$_{(16:0)}$	1.08 ± 0.06	0.97 ± 0.08	1.16 ± 0.24	0.96 ± 0.20	0.75 ± 0.25	0.95 ± 0.23	1.11 ± 0.19
	**lysoPE**$_{(19:0c)}$	1.04 ± 0.06	1.02 ± 0.07	1.09 ± 0.20	1.16 ± 0.19	0.96 ± 0.23	1.14 ± 0.26	1.06 ± 0.19
	**lysoPG**$_{(18:1)}$	0.93 ± 0.05	1.04 ± 0.07	0.80 ± 0.23	1.10 ± 0.25	1.06 ± 0.18	0.99 ± 0.17	0.95 ± 0.30
**R3**	**POPG**	0.95 ± 0.01	1.00 ± 0.02	0.9 ± 0.05	0.98 ± 0.05	1.01 ± 0.06	1.00 ± 0.05	0.95 ± 0.05
	**POPE**	1.06 ± 0.02	0.97 ± 0.04	1.13 ± 0.07	1.02 ± 0.07	0.95 ± 0.07	1.01 ± 0.06	1.05 ± 0.06
	**POPA**	0.95 ± 0.05	1.13 ± 0.08	0.86 ± 0.24	1.23 ± 0.22	1.08 ± 0.23	0.94 ± 0.17	1.04 ± 0.20
	**lysoPE**$_{(18:1)}$	1.04 ± 0.05	1.03 ± 0.08	1.24 ± 0.21	1.01 ± 0.32	0.99 ± 0.19	0.88 ± 0.22	1.06 ± 0.21
	**lysoPE**$_{(16:0)}$	1.07 ± 0.06	0.95 ± 0.08	1.09 ± 0.24	0.98 ± 0.18	1.04 ± 0.30	0.98 ± 0.22	1.14 ± 0.20
	**lysoPE**$_{(19:0c)}$	1.07 ± 0.06	1.02 ± 0.07	0.95 ± 0.17	0.88 ± 0.22	0.99 ± 0.22	1.01 ± 0.24	1.18 ± 0.18
	**lysoPG**$_{(18:1)}$	0.95 ± 0.05	1.0 ± 0.06	0.98 ± 0.19	1.0 ± 0.20	1.08 ± 0.19	1.1 ± 0.18	0.71 ± 0.30

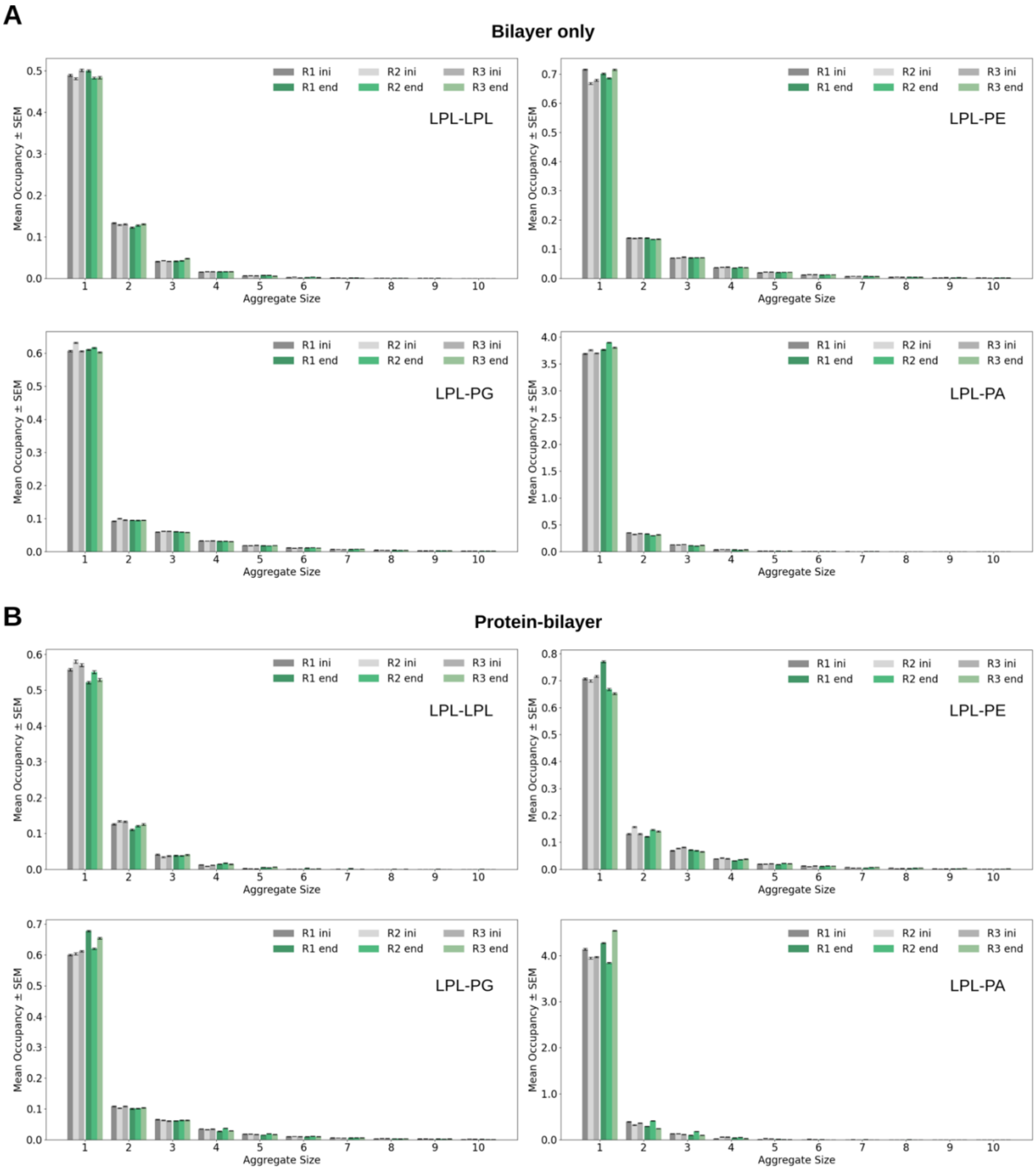

Figure S5: Aggregation propensity of LPLs with different lipid types. The frequency of observed aggregates was computed for each replica separately for the first and last 200 ns of the production run. Values are normalised by the total number of molecules of the second lipid species in the plot label. Plots are separated by phospholipid types in contact with LPL: POPA (LPL-PA), POPG (LPL-PG) and POPE (LPL-PE), or lysophopholipids (LPL-LPL). For clarity only aggregates up to size 10 are shown. Aggregates larger than 10 (if existed at all) were insignificant.

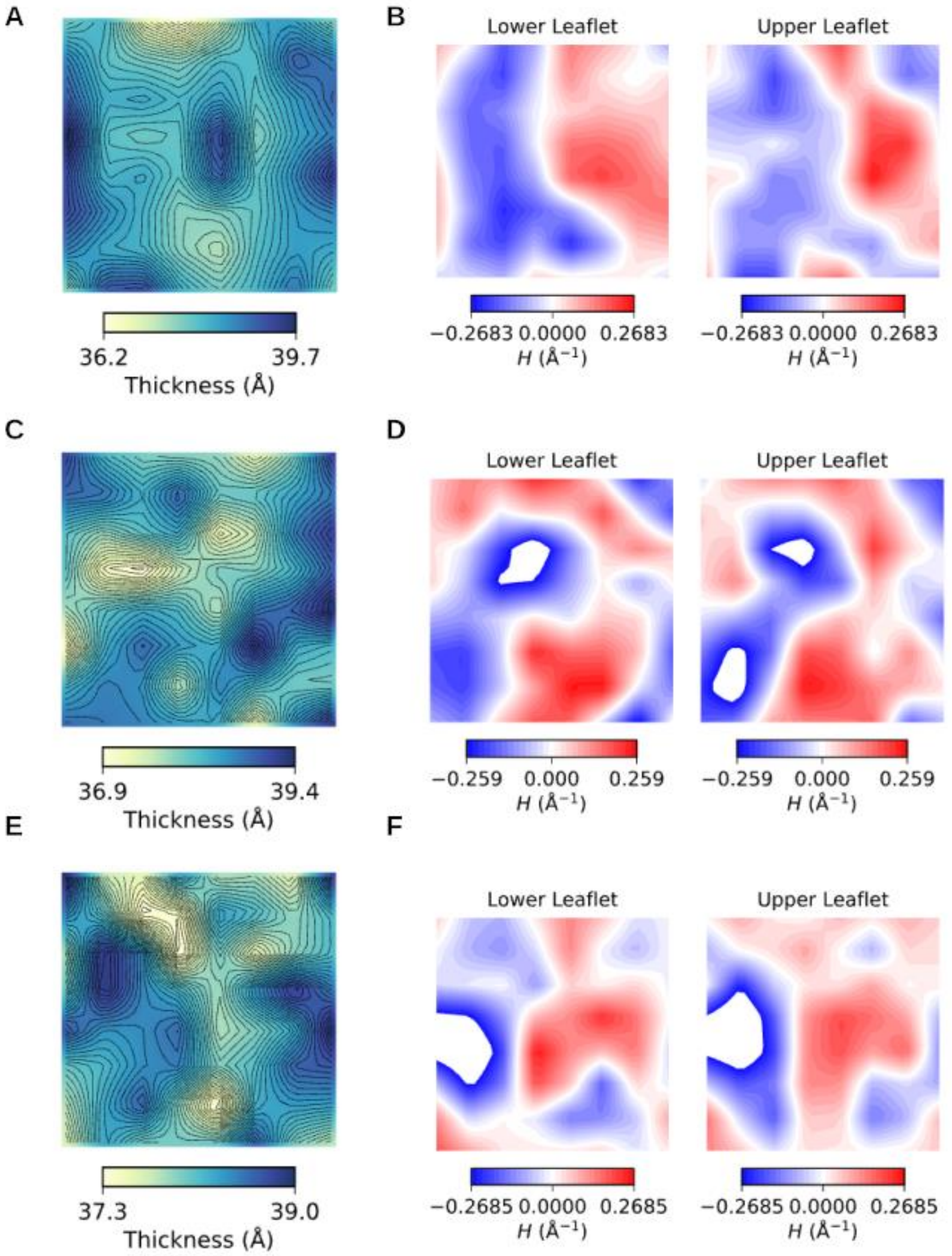

Figure S6: Mean Thickness and Mean Curvature for all 3 replicas (R1-R3) of 20% LPL bilayer systems.The last 100 ns of the production run were used to compute the average thickness and the mean curvature. (A,C,E) Average local thickness for R1, R2, and R3 respectively, projected on to the bilayer plane. (B,D,F) Plots of mean curvature for the lower and upper leaflet of the studied systems

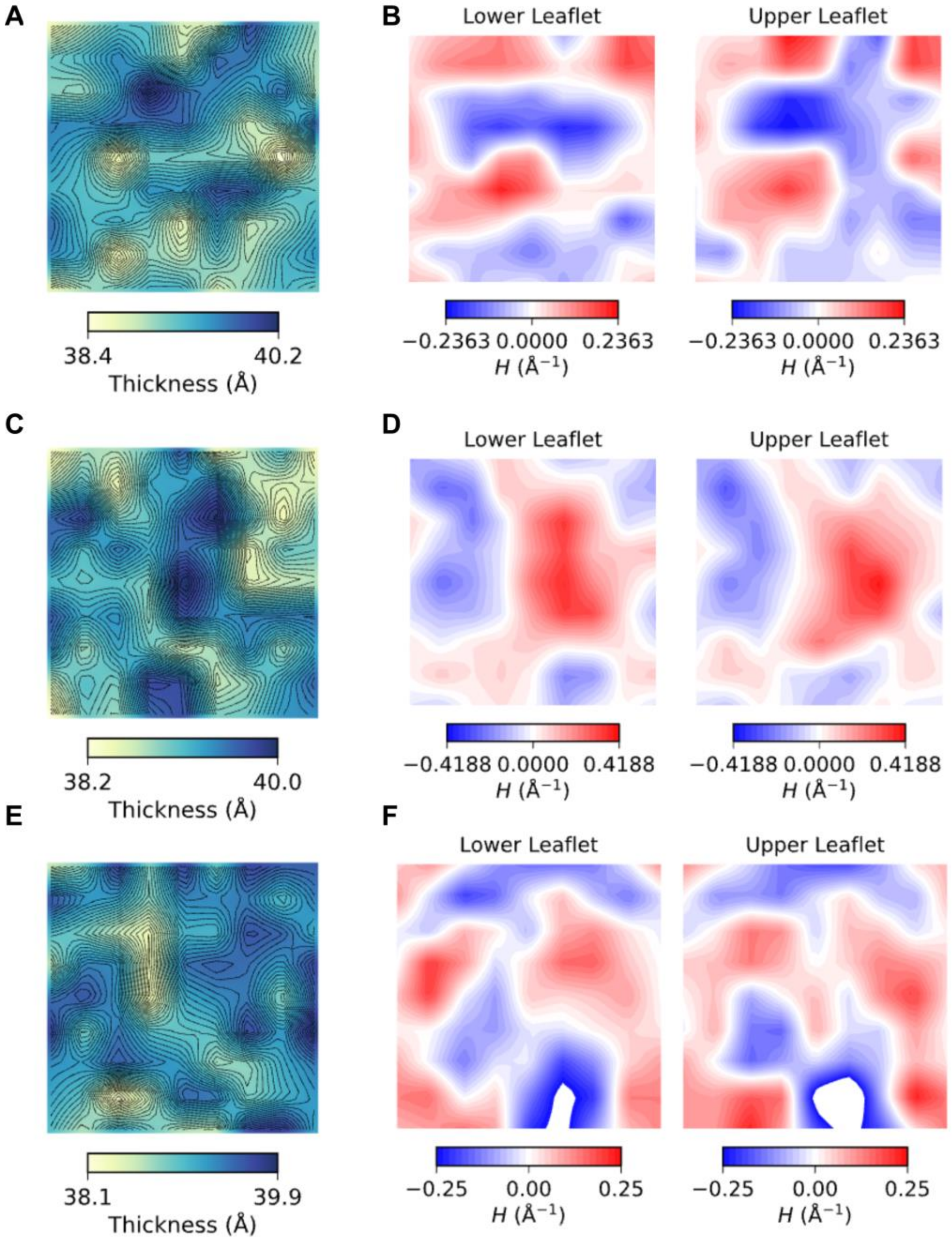

Figure S7: Mean Thickness and Mean Curvature for all 3 replicas (R1-R3) of the bilayers containing only POPA, POPG, POPE. The last 100 ns of the production run were used to compute the average thickness and the mean curvature. (A,C,E) Average local thickness for R1, R2, and R3 respectively, projected on to the bilayer plane. (B,D,F) Plots of mean curvature for the lower and upper leaflet of the studied systems

Table S10: Area compressibility moduli for bilayer systems. A block analysis was performed for the last 500 ns of the respective trajectories. Blocks of 100 ns were used, the means for each replica are reported below with their associated standard errors in brackets.

	**K_A (mN/m)**	
	**Phospholipids only**	**20 % LPL**
**R1**	201 (16)	200 (2)
**R2**	223 (13)	210 (26)
**R3**	202 (6)	186 (12)

Membrane Protein

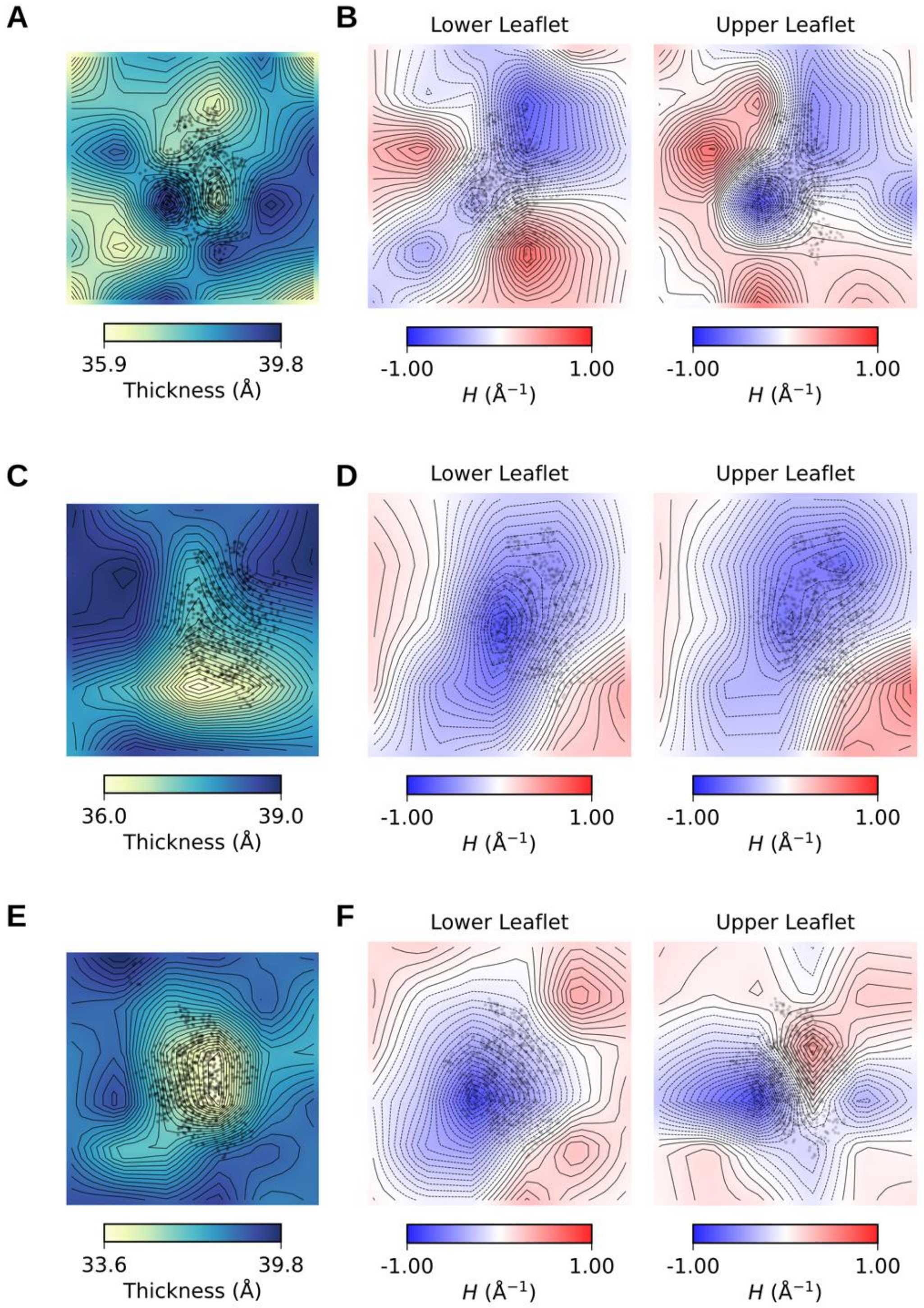

Figure S8: Mean Thickness and Mean Curvature for all 3 replicas (R1-R3) of Pglb-Bilayer systems.The last 100 ns of the production run were used to compute the average thickness and the mean curvature. (A,C,E) Average local thickness for R1, R2, and R3 respectively, projected on to the bilayer plane. (B,D,F) Plots of mean curvature for the lower and upper leaflet of the studied systems. In all plots the average protein positions are also plotted as black transparent dots for reference.

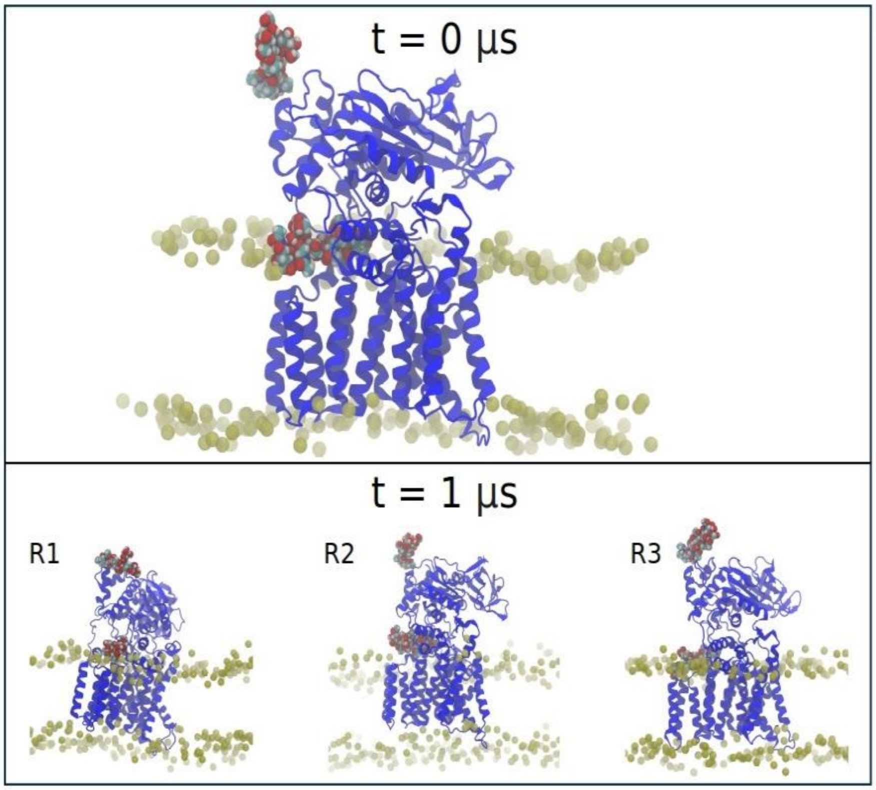

Figure S9: Zoomed-in sideviews of Initial and final snapshots of PglB/mixed bilayer for each of the replicas (R1-R3). PglB is coloured in blue cartoon representation, the glycans are depicted as coloured spheres and lipid phosphorus atoms are depicted as brown spheres.

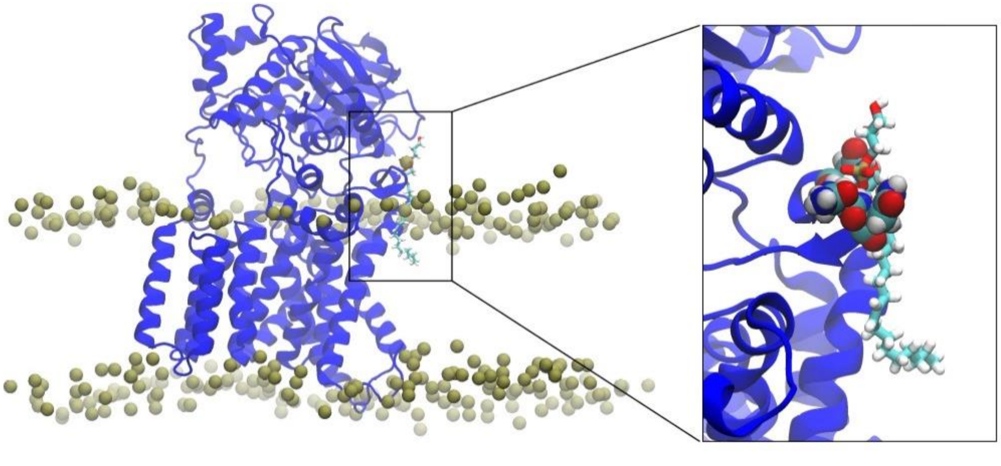

Figure S10: Final snapshot of R1 replica of PglB/mixed bilayer highlighting LysoPG$_{(18:1)}$ lipid (sticks) is located outside the bilayer alongside PglB (blue cartoon representation). Asparagine residues forming a 'cage' of interactions with the extracted LysoPG$_{(18:1)}$ are shown as spheres in the zoomed-in box. Lipid phosphorus atoms are depicted as brown spheres. Some phosphorus atoms in the immediate plane of vision in front of the protein have been omitted for clarity.

Table S11: Average Sugar-lipid contacts over the whole trajectories

**Molecule**	**Average Sugar-lipid contacts**		
	**R1**	**R2**	**R3**
**POPG**	0.416 ± 0.008	0.736 ± 0.011	1.021 ± 0.011
**POPE**	1.591 ± 0.014	0.386 ± 0.009	1.512 ± 0.013
**POPA**	0 ± 0	0.159 ± 0.005	0.266 ± 0.006
**LysoPE**(18:1)	0.0204 ± 0.002	0.137 ± 0.005	0.184 ± 0.006
**LysoPE**(16:0)	0 ± 0	0.042 ± 0.003	0 ± 0
**LysoPE**(19:0*c*)	0.002 ± 0.001	0.091 ± 0.004	0.014 ± 0.002
**LysoPG**(18:1)	0.019 ± 0.002	0.214 ± 0.006	0.021 ± 0.002

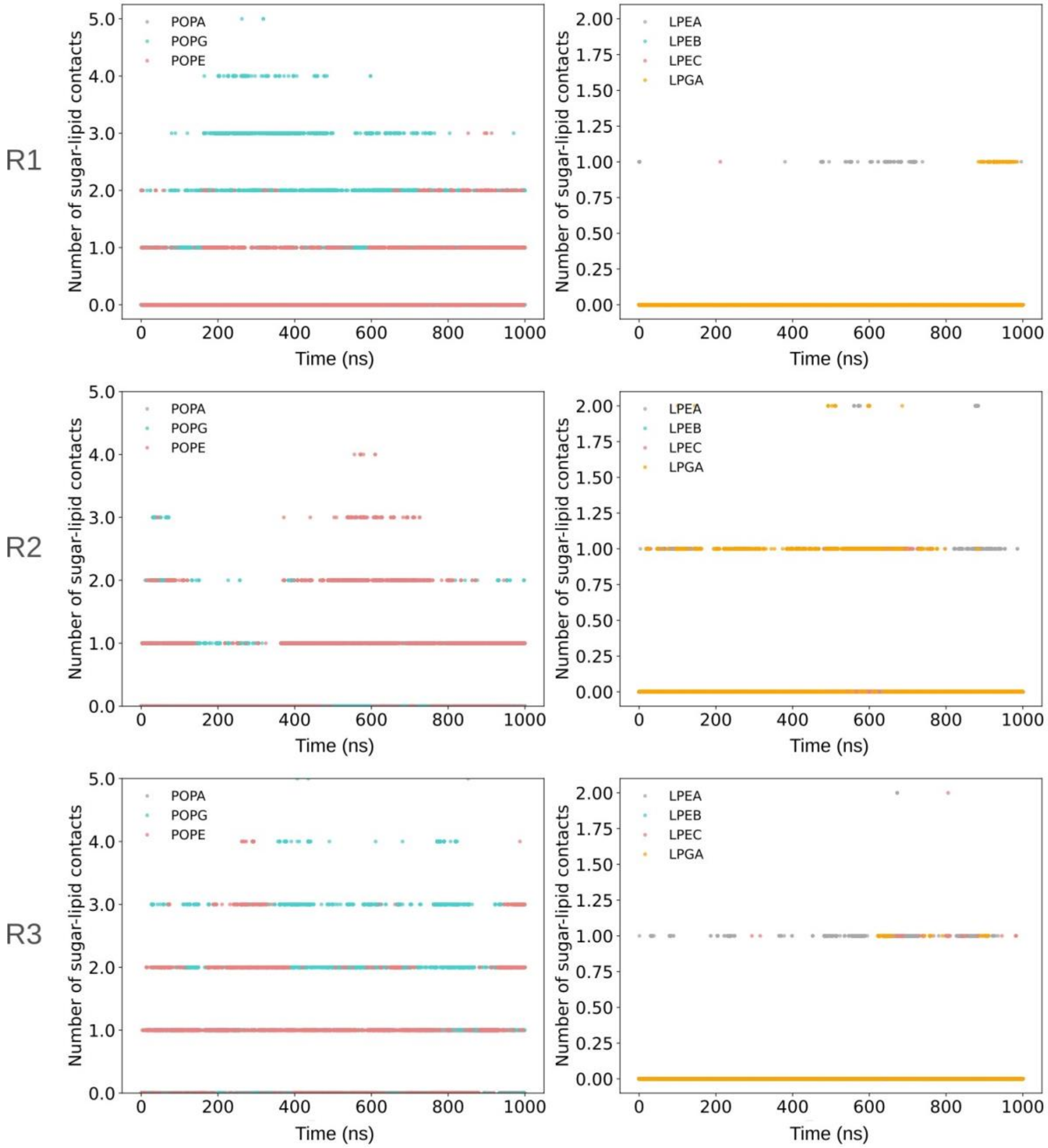

Figure S11 Contacts were computed between the sugar moieties of the LLO donor lipid and any lipid atom of the corresponding lipid type. Plots are separated for either phospholipids: POPA, POPG and POPE, or lysophopholipids: LPEA: $LysoPE_{(18:1)}$, LPEB: $LysoPE_{(16:0)}$, LPEC: LysoPE(19:0*c*), LPGA: LysoPG(18:1)

Electroporation Simulations

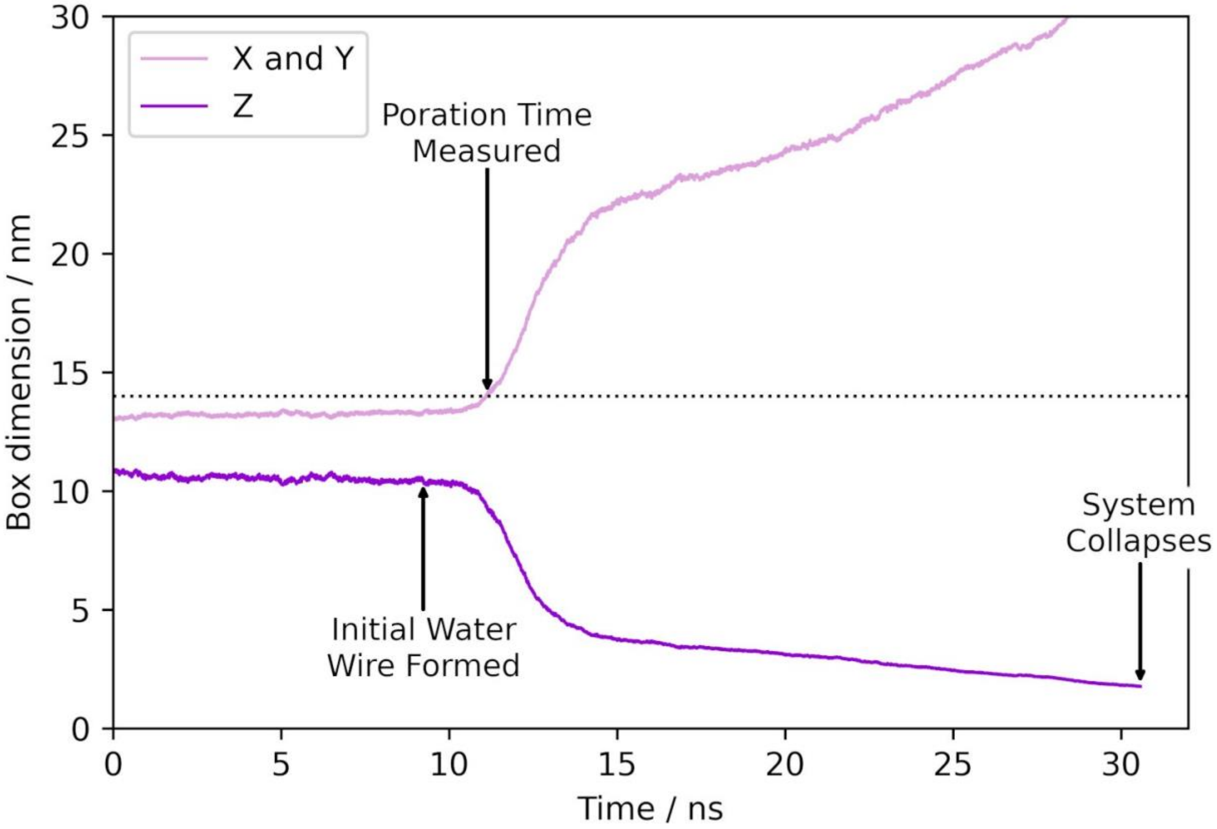

Figure S12: Box dimensions during electroporation of a mixed bilayer (R1, field strength 0.15 V nm $^{-1}$). The formation of the water wire has a negligible effect on the box dimensions, but as lipid headgroups move into the hydrophobic core to stabilise the water channel the bilayer expands in the *xy*-plane. The poration time was measured as the point at which the *x* (and *y*) box dimension increased to > 10% above the equilibrium box dimension; at this point, a substantial water channel has formed in the bilayer.

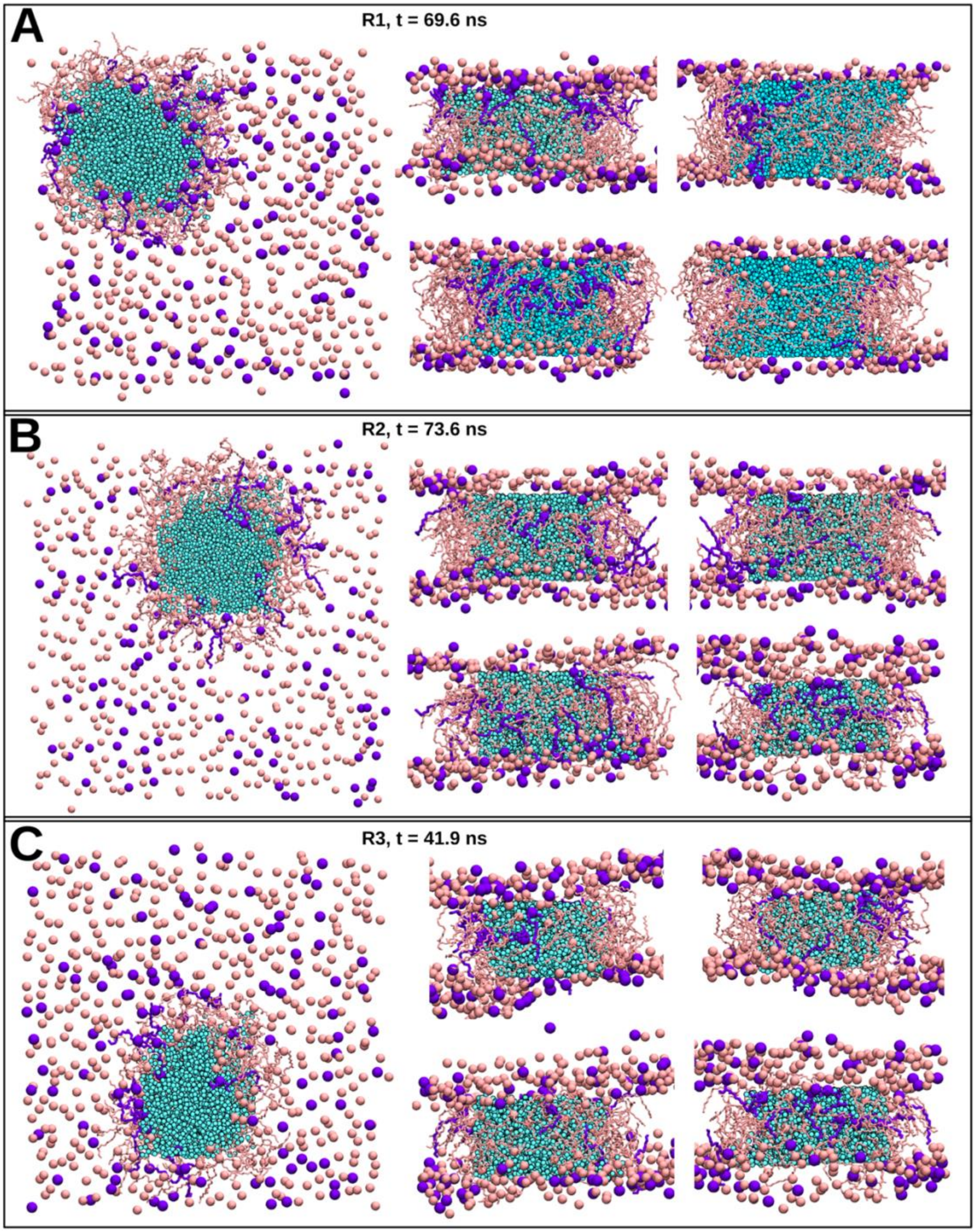

Figure S13: Simulation snapshots from the respective pore formation times reported for a field strength E= 0.175V nm^{-1}; the lowest studied strength that generated pores in all 3 replicas. (A,B,C) Top and side views for replicas R1, R2, and R3 respectively. The colouring scheme follows the same as in Fig.11: POP, POPG, POPA in pink; LPLs in purple; oxygen atoms from water inside the water pore are depicted as cyan spheres. Only the lipids in direct contact with the water in the pore are shown as sticks. All the phosphorus atoms from the lipids are shown as spheres.

Table S12: LPL ratio from lipids in contact with waters forming the initial water wire or the reported pore.

Replica	E= 0.15 Vnm^{-1}				E= 0.175 Vnm-1			
	water wire	# LPL; # PL	pore	# LPL; # PL	water wire	# LPL; # PL	pore	# LPL; # PL
**R1**	0.25	5; 15	0.23	18; 61	0.25	5; 15	0.21	20; 75
**R2**	-				0.16	5; 27	0.20	15; 63
**R3**	0.23	6; 20	0.26	22; 61	0.04	1; 24	0.19	12; 50
