# Supplementary material for: Atomistic modeling of lysophospholipids from the Campylobacter jejuni lipidome
Source: Biophys J. 2025 Aug 28;124(19):3227–43. doi: 10.1016/j.bpj.2025.08.024 (PMC12709257; doi:10.1016/j.bpj.2025.08.024)
Supplement: Document S2. Article plus Supporting Material [file mmc2.pdf]

Biophysical Journal
Article

Biophysical Society

Atomistic modeling of lysophospholipids from the *Campylobacter jejuni* lipidome

Astrid F. Brandner,[2] Kahlan E. Newman,[1] Jonathan W. Essex,[1] and Syma Khalid[1,2,*]
[1]School of Chemistry, University of Southampton, Southampton, UK and [2]Department of Biochemistry, University of Oxford, Oxford, UK

ABSTRACT Lysophospholipids are an important class of lipids in both prokaryotic and eukaryotic organisms. These lipids typically constitute a very small proportion (<1%) of the bacterial lipidome but can constitute 20%–45% of the *Campylobacter jejuni* lipidome under stress conditions. It is thus of importance to include these lipids in model *C. jejuni* membrane simulations for an accurate representation of the lipidic complexity of these systems. Here, we present atomistic models for four lysophospholipids from the *C. jejuni* lipidome, each derived from existing phospholipid models. Herein, we use molecular dynamics simulations to evaluate the ability of these models to reproduce the expected micellar, hexagonal, and lamellar phases at varying levels of hydration. Mixtures of phospholipids and lysophospholipids emulating the *C. jejuni* lipidome under ideal growth conditions were found to self-assemble into bilayers in solution. The properties of these mixed bilayers were compared with those containing only phospholipids: the presence of the selected lysophospholipids causes a subtle thinning of the bilayer and a reduction in area per lipid, but no significant change in lipid diffusion. We further test the mixed bilayer model running simulations in which a native inner membrane protein is embedded within the bilayer. Finally, we show that lysophospholipids facilitate the formation of pores in the membrane, with lysophospholipid-containing bilayers more susceptible to electroporation than those containing only phospholipids.

SIGNIFICANCE Biological membranes serve as mechanical barriers whose structural order, composition, and dynamics vary across organisms, enabling adaptation via lipid regulation. This study developed atomistic models of lysophospholipids found in the inner membranes of *Campylobacter jejuni*, a common foodborne pathogen. These models enable more accurate molecular dynamics simulations of *Campylobacter jejuni* membranes than was previously possible. The models are validated via analysis of key physicochemical properties and dynamics, including computational electroporation assays. Furthermore, a glycosylated native inner membrane native protein is simulated with the new models. Overall, we have expanded the available repertoire of lipids for bacterial membrane simulations.

INTRODUCTION

Bacterial phospholipids have a great degree of compositional heterogeneity. Although many bacterial membranes contain mostly the ubiquitous molecules with a polar headgroup connected to two hydrophobic acyl tails via a glycerol 3-phosphate group, others may have an additional acyl chain or may have just one tail; such lipids are termed acylphospholipids and lysophospholipids (LPLs), respectively. LPLs are intermediates in phospholipid synthesis (1) and metabolites of phospholipid degradation (2–5) (Fig. 1).

LPLs are known to be important in signaling, lipid raft formation, and membrane remodeling in eukaryotes (6). The role(s) of LPLs in bacteria remain poorly understood, although they are thought to be involved in bacterial survival and invasion (6,7). Diverse mixtures of lipids likely aid bacteria in maintaining their shape, which in turn is important for function (e.g., the helical shapes of *Campylobacter* and *Helicobacter* are important for motility in the gut) (8). LPLs have been shown to constitute a substantial proportion of the *C. jejuni* lipidome (9). Under ideal growth conditions the *C. jejuni* lipidome was shown to contain 45% phosphatidylglycerol (PG), 28% phosphatidylethanolamine (PE), 16% lysoPE, 4% PX (unknown headgroup), 3% lysoPG, 2% phosphatidic acid (PA), and 1% acylPG, determined via high-performance

Submitted May 6, 2025, and accepted for publication August 25, 2025.
*Correspondence: syma.khalid@bioch.ox.ac.uk
Astrid F. Brandner and Kahlan E. Newman contributed equally to this work.
Editor: James Gumbart.
https://doi.org/10.1016/j.bpj.2025.08.024
© 2025 The Authors. Published by Elsevier Inc. on behalf of Biophysical Society.
This is an open access article under the CC BY license (http://creativecommons.org/licenses/by/4.0/).

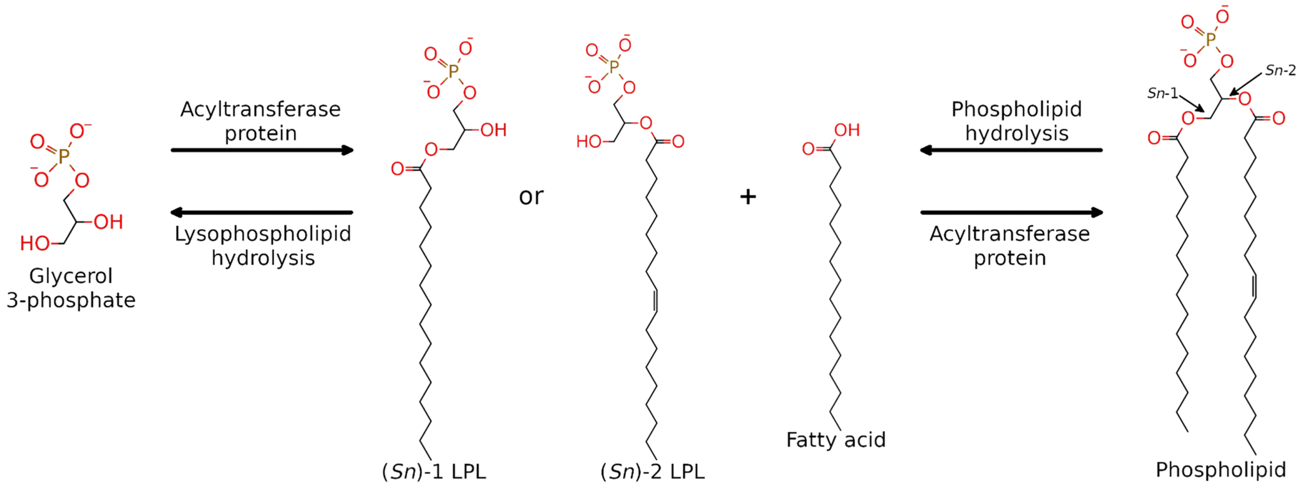

FIGURE 1 LPLs are generated as intermediates in phospholipid biosynthesis and as by-products of phospholipid degradation.

liquid chromatography tandem-mass spectrometry (9). Under stress conditions, the proportion of LPLs increased to as much as 45% (9). This is in contrast to other bacterial species where LPLs are typically found in concentrations below 1% (2). This high lysophospholipid content may be due to the absence of the LplT-Aas "phospholipid repair" system in *C. jejuni* (9): LplT proteins translocate LPLs across the inner membrane of Gram-negative bacteria where they can be re-acylated by the protein Aas (acyltransferase-acyl carrier protein synthetase) on the cytoplasmic face of the membrane (2,10,11). Without this system, LPLs can accumulate in the membranes of the bacterium, thereby perturbing the balance of the lipidome. LPLs are thus of interest for modeling a biologically relevant *C. jejuni* membrane.

Lipid geometry

Phospholipid molecules can adopt a range of shapes based on their headgroup, number of acyl tails, tail length, and tail saturation. LPLs have a single acyl tail and typically self-assemble into micelles in solution (12–15). Pure LPLs are non-bilayer-forming lipids under physiological conditions (16). However, LPLs can stabilize membranes containing multiple lipid types via shape complementarity, for example, in a bilayer rich in inverted conical lipids such as cardiolipin (17). Conversely, LPLs can disrupt membranes through detergent-like character, increasing the permeability of phospholipid bilayers (18–20).

Assemblies can also be affected by pH, salt concentration, temperature, and hydration level (14,15,21). Phase diagrams relating temperature and hydration have been resolved for some LPLs (19): the phase diagrams for 1-palmitoyl-2-hydroxy-sn-glycero-3-phosphatidylcholine ($lysoPC_{(16:0)}$) and 1-stearoyl-2-hydroxy-sn-glycero-3-phosphatidylcholine ($lysoPC_{(18:0)}$) are shown in Fig. 2 (22). In excess water, LPLs typically self-assemble into micelles (M_I phase). As the level of hydration decreases, different phases are known to form. For the lysoPC lipids shown in Fig. 2, hexagonal (H_I), cubic (Q_I), and lamellar (L_{ff}, L_c) phases form as the ratio of water to lipids decreases. Similarly, at low temperatures, lysoPE has been shown to achieve a metastable interdigitated lamellar gel phase (13,14).

Lipid modeling

Due to a lack of readily available models, LPLs were omitted in our previous simulation study of the *C. jejuni* outer membrane (23). However, due to their wide-ranging effects on membranes and embedded proteins (24,25), as well as their high concentration in this bacterial lipidome, we feel it important to include this family of lipids in further *C. jejuni* membrane models. Four of the most abundant LPLs were selected from the *C. jejuni* lipidome (9) for modeling: lysoPE (16:0, 18:1, and 19:0c) and lysoPG (18:1) (Fig. 3).

The development of parameters for individual molecular models is ordinarily an iterative process, wherein an initial model is refined to better match experimental (or quantum mechanical) data (26). However, experimental data for all but the most common lipid species are sparse; to the best of our knowledge, there are no experimental data available to iteratively improve any models we generate specifically relating to the selected lipids. It is also important to note that quantum mechanical (QM) data alone are insufficient for parameterization of large biomolecules (27). Although ab initio data are useful to parameterize quantities such as dihedral energy surfaces and optimal bond lengths, other properties important for the biophysics of lipids such as phase behavior can often be usefully captured using empirical data. We will therefore qualitatively evaluate our models against general properties of lysophospholipids.

To this end, we have produced models for four LPLs by modifying existing, experimentally validated lipid models in the CHARMM36m force field, with the aim to generate more diverse bilayers that better represent the complexity of *C. jejuni* membranes. Here, we have tested these models via aqueous self-assembly simulations and by evaluating their effects on the properties of phospholipid bilayers.

MATERIALS AND METHODS

All MD simulations were performed in the CHARMM36m force field (28–30) with modified TIP3P water (31) in GROMACS 2021.5 (32,33). Hydrogen to heavy atom bonds were constrained using LINCS (34). Long-range electrostatics were treated using Particle Mesh Ewald (35) (1.2-nm cutoff). van der Waals interactions were smoothed to zero between an inner cutoff of 1.0 nm to a final cutoff of 1.2 nm. Unless otherwise stated, the velocity rescale thermostat (36) ($\tau_T = 1.0$ ps) was used to couple systems to a heat bath; pressure coupling (full details in subsequent sections) used a time constant, τ_p, of 2 ps and compressibility b of $4.5e^{-5}$ bar^{-1}; and a timestep of 2 fs was used throughout. Analyses were performed using GROMACS, MDAnalysis (including MembraneCurvature package), and LiPyphilic utilities (37–41) and in-house scripts. To check the formation of lipid aggregates, a cutoff of 0.9 nm between phosphorus atoms was used to define a contact, and an aggregate was defined as the group of molecules that are connected via a contacting lipid. Molecular graphics were generated in VMD 1.9.4a55 (42). Aggregates split across periodic boundaries were reassembled using FixBox (43).

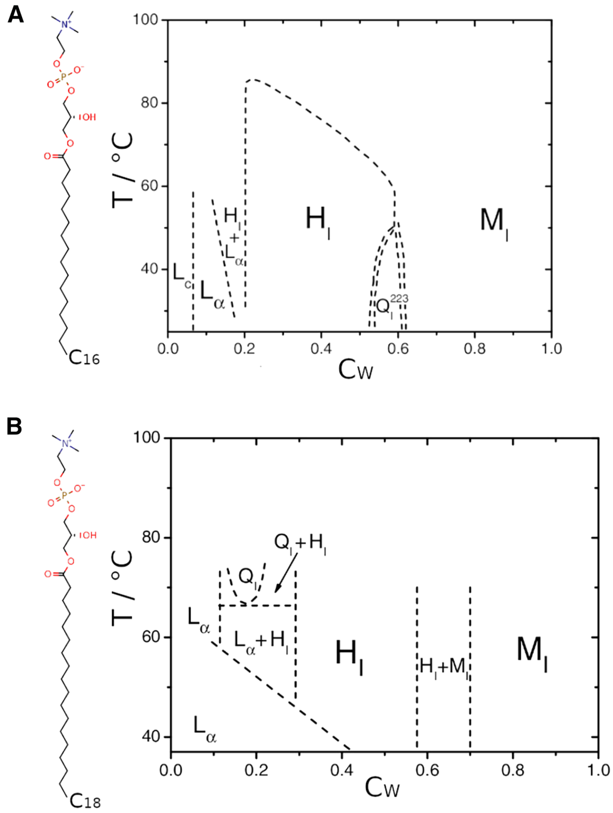

FIGURE 2 Phase diagrams for lysoPC lipids (14), adapted from (15). L_C, solid crystalline phase; L_{ff}, liquid crystalline (fluid) bilayer phase; Q^x_I, cubic phase; H_I, hexagonal phase; M_I, micellar phase. (*A*) Structure and phase diagram of 1-palmitoyl-2-hydroxy-sn-glycero-3-phosphatidylcholine (lysoPC, 16:0). (*B*) Structure and phase diagram of 1-stearoyl-2hydroxy-sn-glycero-3-phosphatidylcholine (lysoPC, 18:0). Both of these LPLs aggregate into micelles in excess water but form hexagonal, cubic, and lamellar phases when the water content by weight (C_W) is reduced.

Model generation

Models were derived from existing phospholipid models in the CHARMM36m force field (29,30,44). LysoPE lipids were generated by modifying 1-palmitoyl-2-oleoyl-sn-glycero-3-phosphoethanolamine (POPE) and 1-palmytoil-2-cis-9,10-methylenehexadecanoyl-phosphatidylethanolamine (PMPE) models; lysoPG was generated by modifying the 1-palmitoyl-2-oleoyl-sn-glycero-3-phosphoglycerol (POPG) model. In each case, the relevant lipid tail was removed, and the linking ester group replaced with a hydroxyl group. Parameters for the O-H bond and hydroxyl hydrogen were taken from the CHARMM36m model for the lysophospholipid 1-myristoyl-2-hydroxy-snglycero-3-phospho-(1′-rac-glycerol) (LMPG) (saturated C_{14} tail). Where necessary, the partial charge of the headgroup phosphorus atom was modified to yield the correct net integer charge. This was the case for lysoPG$_{(18:1)}$ (named LPGA in the topologies), where the charge was reduced by 0.005 e. The resulting models were energy minimized as individual molecules in vacuo in 5000 steps using steepest descent (45).

Lipid tail order parameters

The tail order parameter, S_{CD}, is a measure of the orientational order of lipid acyl chain(s) in a bilayer. This can be calculated experimentally using quadrupolar splitting measured from deuterium NMR experiments (46,47), and/or it can be extracted from MD trajectories (48–52). The order parameter for each carbon atom in the acyl chain is calculated as follows (46,47):

$$S_{CD} = \frac{1}{2}\langle 3\, cos^2\ \theta_{CD} - 1 \rangle, \tag{1}$$

where θ is the angle between the bilayer normal (*z*) and the carbon-hydrogen/deuterium bond vector. Angular brackets indicate the ensemble average (molecular and temporal). Where the carbon has more than one bonded hydrogen, S_{CD} may be calculated for each individual C-H bond, though through rotation these hydrogens are generally considered equivalent, and the average C-H bond vector is used. A value of 0 indicates an isotropic ensemble with no overall preference in orientation, with positive and negative values indicating alignment with the bilayer normal or bilayer plane, respectively.

Although there are several tools available to calculate order parameters (53–56), many rely on a united-atom approach, which yields inaccurate results for unsaturated carbons (51). Due to these inaccuracies, and difficulties in implementing existing all-atom tools, an in-house MDAnalysis script was used (available on Zenodo; see supporting material).

Area compressibility

The area compressibility, K_A, was computed as

$$K_A = k_B T \frac{\langle A \rangle}{\langle \delta A^2 \rangle}, \tag{2}$$

where k_B is the Boltzmann constant, T the temperature, <A> the average total area, and < δA^2> the mean-square fluctuation (57). A block analysis was performed to obtain the means and standard errors for each system. Only the last 500 ns of simulation was considered for this analysis, using blocks of 100 ns.

Self-assembly simulations

How the LPLs assemble in solution was assessed by simulating the lipids in water boxes. Lipid molecules were placed randomly in a simulation box

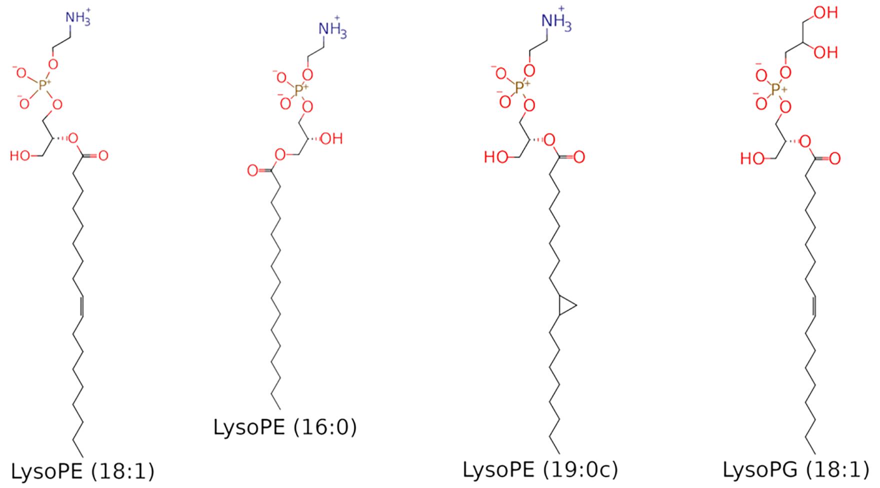

FIGURE 3 Structures of the lysophospholipids modeled. Three lysoPE lipids and one lysoPG lipid were selected from the *C. jejuni* lipidome.

using the GROMACS utility insert molecules, with neutralizing counterions (K^+) plus additional KCl in solution (50–150 mM, system dependent). Several setups were explored, detailed in the following subsections. The contents and initial unit cell sizes of all simulated systems are detailed in the supporting material (Tables S1–S4). All self-assembly simulation cells were initially cubic.

Small single-lysophospholipid systems

In this setup, each box contained 140 lysophospholipid molecules, plus ions and water molecules such that water weight fraction (C_W) was either $C_W \sim$ 0.65–0.7 (ratio of lipids to water, 1:50), $C_W = 0.4$ (ratio ~1:17.8–20.5), or $C_W = 0.1$ (ratio ~1:2.8–3.4). Three unique box configurations were generated for each lipid at each concentration. Systems were energy minimized in 5000 steps (steepest descent (45)). This was followed by NVT and NPT equilibration stages. In the NVT stage, the system was coupled to a heat bath at 315 K for 100 ps, with position restraints of 20 kJ mol^{-1} nm^{-2} on all lipid heavy atoms. The NPT stage introduced pressure coupling via the isotropic Parrinello-Rahman barostat (58,59) to bring the system to a target pressure of 1 bar over 100 ps. Temperature coupling was maintained during the NPT stage. Each system was then simulated at 315 K under the isotropic Parrinello-Rahman barostat at 1 bar. Simulations lasted 1 μs for excess water ($C_W = 0.65$–0.7) and 1.5 μs for C_W of 0.4 or 0.1. One replicate for each lipid species at C_W 0.1, 0.4 was extended to 2 μs.

Larger single-lipid systems

Larger systems containing more lipids were also simulated. For each lysophospholipid, three unique configurations were generated containing the following: 500 lipid molecules; water such that $C_W = 0.7$, 0.4, or 0.1; and neutralizing counterions and KCl to 80–150 mM (system dependent). Systems were energy minimized and equilibrated as above. To allow the volume and density of the box to stabilize, a 5-ns unrestrained simulation under the conditions as described for the small single-lysophospholipid systems (isotropic pressure coupling) was applied after the equilibration stages. Each system was then simulated with anisotropic pressure coupling at 1 bar (Parrinello-Rahman). Simulated annealing was applied to discourage "trapping" of the systems in metastable phases: each system was simulated at 315 K for 40 ns followed by 10 ns at 400 K, cycling for 500 ns in total.

To check for any bias originating from the barostat, a subset of these larger single-lipid systems were also simulated with isotropic pressure coupling. One simulation of the most concentrated (Cw = 0.1) and dilute (Cw = 0.7) system for each LPL type was performed (Tables S1 and S3). The protocol for these simulations followed the original large self-assembly protocol up to the first NVT equilibration. It was followed up by two NPT equilibration steps: 1) a 50-ns-long simulation with Berendsen isotropic pressure coupling at 1 bar and V-rescale temperature coupling (315 K); 2) a 50-ns-long equilibration with Parrinello-Rahman isotropic pressure coupling at 1 bar and with a timestep of 1 fs. Finally, the production run was set to 200 ns (timestep = 2 fs), following the same conditions as in the anisotropic coupling, except for using isotropic pressure coupling in this instance.

Small mixed-lipid systems

To assess whether a bilayer containing both phospholipids and LPLs would form spontaneously, we repeated the above isotropic simulations (small single-lysophospholipid systems) with phospholipids and phospholipid-lysophospholipid mixtures. Three unique systems were established for each pure phospholipid species: 140 POPE, 1-palmitoyl-2-oleoyl-sn-glycero-3-phosphatidic acid (POPA), or POPG molecules were each placed randomly in boxes with 50 water molecules per lipid, counterions and KCl to 80 mM. Three unique boxes containing a lipid mixture with 20% LPLs were also generated: molecules of POPG, POPE, POPA, and the four LPLs were placed in the simulation box in a 9:6:1:1:1:1:1 ratio. These systems were energy minimized, equilibrated, and simulated for 1 μs under the conditions described for the small single-lysophospholipid systems.

Equilibrium bilayer simulations

The self-assembled 20% LPLs bilayer systems were tiled in a 3 × 3 grid (i.e., nine copies of the above self-assembled bilayers) to yield three larger bilayers. The three smaller self-assembled replicates were combined in three unique configurations, with some tiles reflected in the *xy* plane to minimize asymmetry in lipid content across the leaflets, and with a small gap between tiles to avoid steric clashes. The final compositions of all the systems are reported in Table S5. After energy minimization (5000 steps steepest descent), these gaps in the bilayer were closed using 2 × 1 ns NPT stages at 300 K. Position restraints were applied to the *z* coordinates of lipid heavy atoms to allow only lateral translation. Systems were coupled to heat and pressure baths using the Berendsen regime (60) in both stages. A semi-isotropic barostat was employed; a pressure of 1.2 bar was applied in the *xy* plane ($\beta = 4.5e^{-3}$ bar $^{-1}$) to encourage compression, and a pressure of 1 bar was applied along the *z*-axis ($\beta = 4.5e^{-5}$ bar^{-1}). Position restraints (500 kJ mol^{-1} nm^{-2}) were applied to lipid P and C/N/O atoms in the first nanosecond, and restraints of 200 and 50 kJmol^{-1} nm^{-2}, respectively, were applied in the second nanosecond. Each bilayer was then solvated in 150 mM KCl with additional neutralizing counterions. Three analogous phospholipid-only bilayers (POPG, POPE, POPA, 9:6:1) of comparable size were generated using CHARMM-GUI Membrane Builder (61–63).

Systems were energy minimized in 50,000 steps (steepest descent) and then equilibrated sequentially using two NVT and four NPT stages, decreasing position restraint strength at each stage (Table S6). All NPT stages coupled the system to a pressure bath at 1 bar using the semiisotropic Berendsen scheme ($\tau_p = 5.0$ ps). This was followed by 1-μs unrestrained production simulation. Systems were coupled to a temperature bath of 315 K using the Nosé-Hoover thermostat ($\tau_T = 1.0$ ps) and a pressure bath of 1 bar using the semiisotropic Parrinello-Rahman regime ($\tau_p =$ 5.0 ps).

Native protein embedded in bilayer system

SWISS-MODEL (64) was used to fit the PglB sequence of *C. Jejuni* to the *C. lari* PglB structure (PDB: 5OGL (65)). Default protonation states were assigned for all residues in this model. PglB was glycosylated using the CHARMM-GUI PDB Reader and Manipulator module (61,66,67) with the following glycan sequence: GalNAc-α1,4-GalNAc-α1,4-[Glc-β1,3]GalNAc-α1,4-GalNAc-α1,4-GalNAc-α1,3-diNAcBac-β1, where diNAcBac is N′,N′-diacetylbacillosamine [2,4-diacetamido-2,4,6 trideoxy-glucopyranose]; GalNAc is N-acetylgalactosamine; and Glc is glucose. This template contained two magnesium ions, a donor substrate (lipid-linked oligosaccharide (LLO), with a truncated lipid tail), and an acceptor peptide. The magnesium ions were maintained at the locations from the template. The structure of the acceptor peptide for PglB was extracted from the x-ray structure PDB: 5OGL (65). The sequence was modified to match that of the known occupied CmeB glycosite (KDRNVSAD) using in silico mutations in PyMOL (68). The glycan donor LLO was generated in CHARMM-GUI Ligand Modeler (69) with the lipid tail of the LLO truncated to the same length as that in the PglB crystal structure. The lipid tail and bacillosamine unit were aligned with those of the crystal structure. The remaining six moieties of the heptasaccharide were manually arranged. This substrate-bound protein was energy minimized (5000 steps steepest descent) to resolve any steric clashes and inappropriate bond lengths/angles/dihedrals that had arisen. The complex containing PglB was embedded in a membrane patch obtained from a previously equilibrated 20% LPL mixed bilayer, so as to match the hydrophobic transmembrane domains of the protein with the bilayer core. The full system was energy minimized via steepest descent algorithm for 50,000 steps, equilibrated in NPT with a 1-fs timestep for 100 ps using a Berendsen barostat with semiisotropic coupling (1 bar, $\tau_p = 5.0$ ps), and the temperature was regulated via a Berendsen thermostat ($\tau_t = 1.0$ ps, reference temperature of 315 K). Three replicas were subjected to the production run protocol, firstly for 1 ns only, and subsequently, each of the replicas was run for 1 μs for analysis with a 2-fs integration timestep. The full details of these systems are available in Table S7. In the production run, systems were coupled to a temperature bath of 315 K using the V-rescale thermostat ($\tau_t = 1.0$ ps) and a pressure bath of 1 bar using the semiisotropic Parrinello-Rahman regime ($\tau_p = 5.0$ ps, compressibility $= 4.5e^{-5}$ bar^{-1}). GROMACS rms and rmsf tools, MDAnalysis, and fatslim (70) were used to create in-house scripts for data analysis. Phosphorus atoms were used to define the headgroups for thickness and area per lipid analysis. A contact between sugar and lipid was computed if the distance between the sugar moieties of the LLO donor lipid and any lipid atom of the corresponding lipid type was within 0.4 nm. The contacts were only counted once per glycan-lipid interaction, regardless of the total number of atoms in contact. When mean values and their associated errors are reported, unless otherwise stated, values were computed for each replica based on the last 500 ns of production run.

Electroporation simulations

Three mixed phospholipid-LPL bilayers were generated as described in the previous section using a 2 × 2 grid (four tiles total). In this case, each bilayer was generated from four copies of a single self-assembled bilayer replicate; two tiles were flipped in the xy plane to generate fully symmetric bilayers. Three bilayers of comparable size containing only phospholipids (9:6:1 POPG, POPE, POPA) were generated in CHARMM-GUI Membrane Builder. Bilayers were solvated in 150 mM KCl, minimized, equilibrated, and subjected to 100-ns unrestrained equilibrium simulation as described for the 3 × 3 tiled bilayers. An electric field was then applied to each system along the *z*-axis. Field strengths of 0.100, 0.125, 0.150, 0.175, and 0.200 V nm^{-1} were applied, and the time taken for each bilayer to electroporate was measured.

Each of the porated bilayers generated in the 0.200 V nm^{-1} simulations (3× phospholipid only, 3 × 20% LPLs) was simulated under equilibrium conditions to assess whether the pores would close in the absence of an external electric field. Initial structures for these subsequent simulations were taken as the frame at which the box x dimension had increased to 110% of its equilibrium value. These systems were simulated for 100 ns under the conditions described for the equilibrium bilayer simulations. The system compositions are detailed in Table S8.

RESULTS AND DISCUSSION

Micelle formation in excess water

LPLs in excess water are known to form micelles (Fig. 2) (14,15). Our simulations of these four lysophospholipids are consistent with this observation. Each lipid model was simulated in solution at $C_W = 0.65$–0.7 in a small box (140 lipids) under isotropic pressure coupling for 1 μs (Fig. 4 *A*). Approximately spherical micelles formed in most cases. At the start of most production simulations, multiple micelles form as the hydrophobic tails aggregate; usually, this was two micelles, each containing approximately half of the lipids (Fig. 4 *B*). As the simulations progress, these aggregates combine to form a single micelle containing all 140 LPLs that is roughly spherical (Fig. 4 *C*). In two simulations, a single micelle containing all the lipids formed immediately. In eight of the 12 simulations, these spherical micelles were maintained for the remainder of each simulation.

In 3× lysoPE$_{(19:0c)}$ replicates and a single lysoPE$_{(18:1)}$ replicate, where the shape of the lysophospholipid was less conical, cylindrical micelles formed. These simulations also initially proceed via spherical micelle aggregates (Fig. 4 *A–C*). One side of the micelle then interacts with the opposite side in the adjacent periodic image and a larger structue forms over periodic boundaries (Fig. 4, *D* and *E*).

Further simulations of these lipids in solution in a larger box (500 lipids, $C_W = 0.7$) with anisotropic pressure coupling displayed similar micelle formation. In each case, multiple micelles formed in each simulation box. However, as there were no extended, rigid structures formed over the periodic boundaries, there is a lack of both cohesive forces that would prevent expansion and of repulsive forces that prevent collapse of the simulation cell along a given axis. Box deformation and subsequent collapse (a box dimension became smaller than the electrostatic cutoff) occurred rapidly in all replicates (Fig. S1).

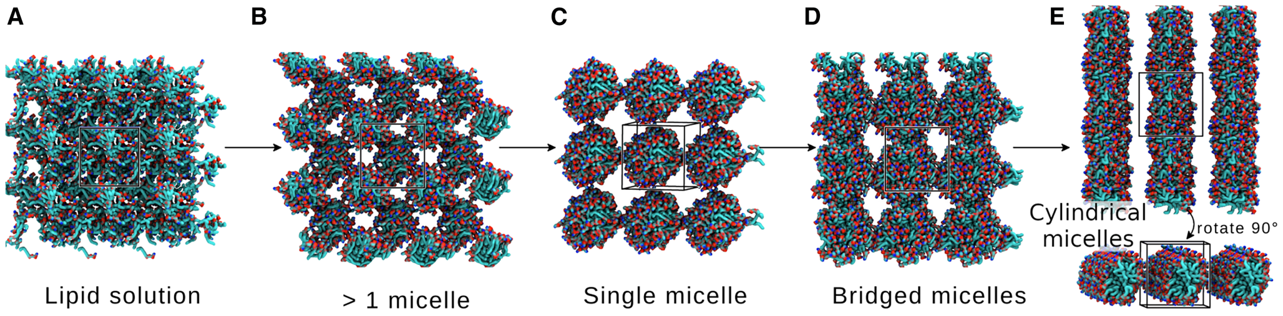

FIGURE 4 Formation of micelles in solution. Snapshots from lysoPE$_{(19:0c)}$ R1, 50 waters per lipid, isotropic pressure coupling. Lipids are shown in surface representation, colored by element. Unit cells are shown with black lines. Water and ions are omitted for clarity. (*A*) Initial lipid solution. (*B*) Lipid tails aggregate to form multiple (two) smaller micelles. (*C*) Smaller micelles combine to form a single spherical micelle. (*D*) Lipid bridges form between one side of the micelle and the opposite side in the adjacent periodic image. (*E*) Bridged micelles rearrange to form cylindrical micelles that are continuous across periodic boundaries in some replicates.

Aggregates at higher lipid concentrations

Small isotropic boxes

LPLs at a similar temperature but higher concentration/lower C_W self-assemble into other phases, such as hexagonal, cubic, and lamellar phases (14,15) (Fig. 2). Each lysophospholipid model was simulated in solution at $C_W = 0.4$ and 0.1 in a small box (140 lipids) under isotropic pressure coupling for 1.5–2 μs to assess whether these phases would also form. These simulations displayed some success in reproducing the expected phases. At $C_W = 0.1$, four of the 12 replicates approach a lamellar phase, with one replicate of lysoPE$_{(19:0c)}$ forming a multilayer L_{ff} system in 1.25 μs (Fig. 5 *A* and *B*). One replicate each of lysoPE$_{(16:0)}$, lysoPE$_{(18:1)}$, and lysoPG$_{(18:1)}$ also approached a multilayered lamellar phase but did not reach the $L_{c/ff}$ phase on the timescales simulated; while a clearly layered aggregate is formed, headgroups remain in what should be an exclusively hydrophobic region (Fig. 5 *A*). As this arrangement is an intermediate stage of the bilayer formation for the lysoPE$_{(19:0c)}$ replicate, it is likely that a lamellar phase may form if these replicates were extended.

The other eight replicates at $C_W = 0.1$ formed a state that resembled neither a lamellar nor a hexagonal phase. Instead, these replicates displayed cubic phases, wherein highly interconnected micelle-like aggregates organize in a primitive-cubic unit (Fig. 5 *C*). Where this state formed, it was stable for the remainder of the simulation.

At $C_W = 0.4$ the aggregates formed can be clustered into two broad categories: cylindrical micelle-based assemblies and bilayer-like assemblies. In six of the 12 systems, cylindrical micelles formed: in one replicate (1xlysoPG$_{(18:1)}$) a hexagonal arrangement of these aggregates is observed, consistent with the expected H_I phase (Fig. 5 *D*). In the other five replicates (3× lysoPE$_{(16:0)}$; 2× lysoPG$_{(18:1)}$), these were in a primitive cubic arrangement (Fig. 5 *E*). The remaining six replicates formed bilayer-like aggregates. All three replicates of lysoPE$_{(18:1)}$ and two replicates of lysoPE$_{(19:0c)}$ were observed to assemble into a porated bilayer (Fig. 5 *F*). The final replicate of lysoPE$_{(19:0c)}$ was observed to enter the L_{ff} phase (Fig. 5 *G*) within the first 200 ns of the simulation. Longer simulations of these systems, than those feasible in the present study, would likely reduce these differences.

The use of isotropic pressure coupling and a small simulation box affects the likelihood of particular phases forming. When the box is small, the requirement for periodic replication can trap the system in a particular phase. Isotropic pressure coupling enforces uniform scaling along all box dimensions; the ability of a given lipid to form a lamellar bilayer will be dependent on selecting an appropriate box size and hydration level or require a multilayered bilayer to form to appropriately span the periodic boundaries. Furthermore, whereas the simulations were relatively long in an attempt to sample more of the available phase space, most replicates became trapped in the first stable/metastable state they sampled. We attempted to address these issues with additional simulations under a different regime.

Larger boxes: Anisotropic simulated annealing

To further assess the formation of these phases, each lipid model was simulated in solution at $C_W = 0.4$ and 0.1 in a larger system (500 lipids) under anisotropic pressure coupling with simulated annealing. The use of anisotropic pressure coupling allows more degrees of freedom for extended structure formation and reduces templating effects as the simulation cell can deform in each dimension independently. Simulated annealing enhances sampling by temporarily increasing the kinetic energy of the system, encouraging the crossing of energy barriers and reducing the risk of the system becoming trapped in a metastable state.

At $C_W = 0.1$, four of the 12 simulations approached a multilamellar assembly (Fig. 6 *A*): two replicates each lysoPE$_{(18:1)}$ and lysoPE$_{(19:0c)}$ reach this state by the end of the 500-ns simulations. A fully lamellar assembly (where all headgroups have retreated from the hydrophobic core)

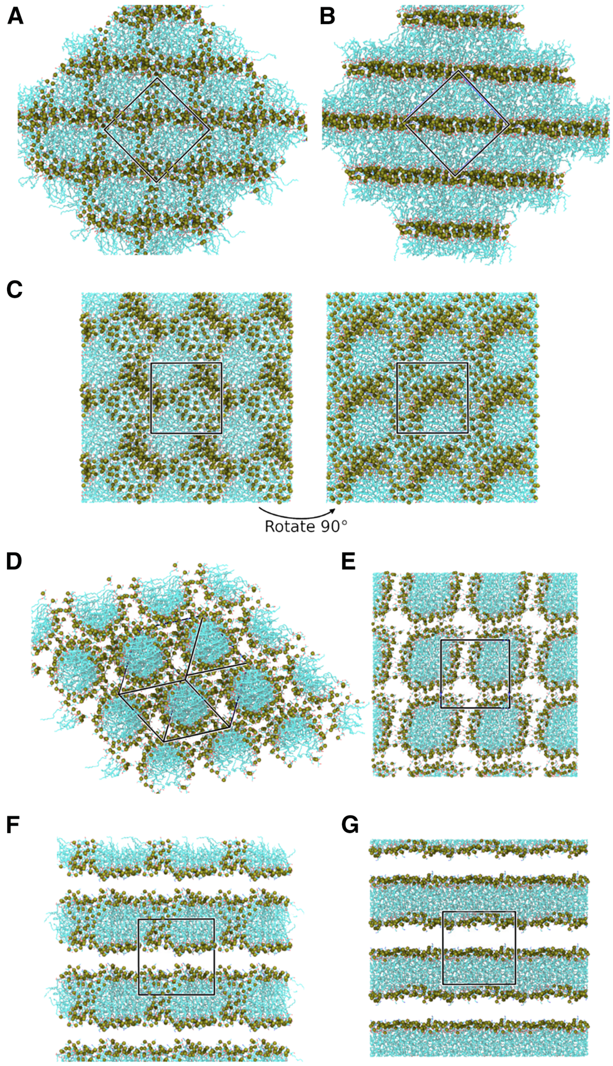

FIGURE 5 Final phases formed by the modeled lysophospholipids at low hydration under isotropic pressure coupling. (*A–C*) snapshots from $C_W = 0.1$ systems. (*D–G*) Snapshots from $C_W = 0.4$ systems. Unit cells are indicated by a black box. Lipids are colored by element in stick representation, with headgroup phosphorus atoms shown as tan spheres. Water and ions are omitted for clarity. (*A*) $\text{LysoPE}_{(18:1)}$ approaching a multilayered lamellar phase; there are still headgroups within the hydrophobic core of the bilayers. One replicate each of $\text{lysoPE}_{(16:0)}$ and $\text{lysoPG}_{(18:1)}$ also reached this phase. (*B*) Multilayered L_{ff} phase formed by a single replicate of $\text{lysoPE}_{(19:0c)}$. (*C*) Cubic phase formed in eight of the 12 simulations at $C_W = 0.1$. (*D*) Hexagonal (H_I) phase formed by a single replicate of $\text{lysoPG}_{(18:1)}$. (*E*) Primitive cubic arrangement of cylindrical micelles, formed by three replicates of $\text{lysoPE}_{(16:0)}$ and two replicates of $\text{lysoPG}_{(18:1)}$. (*F*) A porated bilayer formed by all three replicates of $\text{lysoPE}_{(18:1)}$. (*G*) L_{ff} phase formed by a single replicate of $\text{lysoPE}_{(19:0c)}$.

was not achieved by any replicate on the timescale simulated. All other replicates at $C_W = 0.1$ formed cubic assemblies as observed in the smaller isotropic systems (Fig. 5 *C*).

The $C_W = 0.4$ simulations displayed some diversity in the phases formed. Importantly, two replicates (2× $\text{lysoPG}_{(18:1)}$) achieved the expected H_I phase (Fig. 6 *B*). The remaining replicates formed extended aggregates in two dimensions. In some cases, this was in the form of bilayer-like assemblies that extended across periodic boundaries in one plane, intersected by cylindrical micelles that extend across periodic boundaries along the perpendicular axis (Fig. 6 *C*). In others, cylindrical micelles formed along two axes that intersect through lipid bridges (Fig. 6 *D*). The simulation box collapsed within the first 300 ns in all replicates except those achieving the H_I phase.

We note here that in the additional simulation in which isotropic pressure coupling was used (one simulation of each lipid type in Cw = 0.1 and Cw = 0.7 systems), we saw intermediate states between cubic lattice and multilamellar phase (Fig. S2) whereas for Cw = 0.7, an array of either micelles or bicelle-like aggregates (Fig. S3). No cylindric micelles nor hexagonal phases were observed in the timescales of these simulations. This agrees with the phase diagram for lysoPC lipids in Fig. 2.

Mixed bilayer formation

With the aim of simulating a bilayer more representative of the *C. jejuni* lipidome, we moved to assemble bilayers containing these LPLs. The *C. jejuni* lipidome (under ideal growth conditions) contains ∼20% LPLs (9). We hypothesized that a mixture similar to that described in the literature would self-assemble into a bilayer. We first simulated the phospholipids POPG, POPE, and POPA in excess water (50 waters per lipid) to verify that these existing models were bilayer forming.

Systems containing only POPA, POPE, or POPG in solution all formed bilayers within 400 ns under the simulated conditions. The mechanism of self-assembly was as described by Skjevik et al. (71) (Fig. 7): 1) initial solution of lipids, ions, and water. 2) Tails aggregate to form a micelle-like assembly. Lipid bridges form between one side of the micelle and the periodic image. 3) Bridging lipids insert into the assembly, forming a porous lamellar bilayer. 4) Lipid headgroups retreat from the hydrophobic core to the water-lipid interface; a nonporous bilayer is formed. These bilayers were stable for the remainder of the simulation time (1 μs). PE and PA lipids are of an inverted truncated cone shape and may therefore be expected to form a more thermodynamically favorable inverse hexagonal phase (H_{II}). However, the energetic cost is sufficiently high to prevent the formation of nonlamellar phases under the simulated conditions. Each leaflet may display regions with spontaneous curvature resulting in a membrane presenting curvature frustration (72–74).

We then simulated a phospholipid-lysophospholipid mixture mimicking the proportions found in *C. jejuni* under ideal growth conditions (9). Three systems, each containing 140 lipid molecules, were generated, with 20% lysophospholipid content: 63 POPG (45%); 42 POPE (30%); 7 POPA (5%); 7 $\text{lysoPE}_{(18:1)}$ (5%); 7 $\text{lysoPE}_{(16:0)}$ (5%); 7 $\text{lysoPE}_{(19:0c)}$ (5%); and 7 $\text{lysoPG}_{(18:1)}$ (5%). Similar to the

A

B

C

D

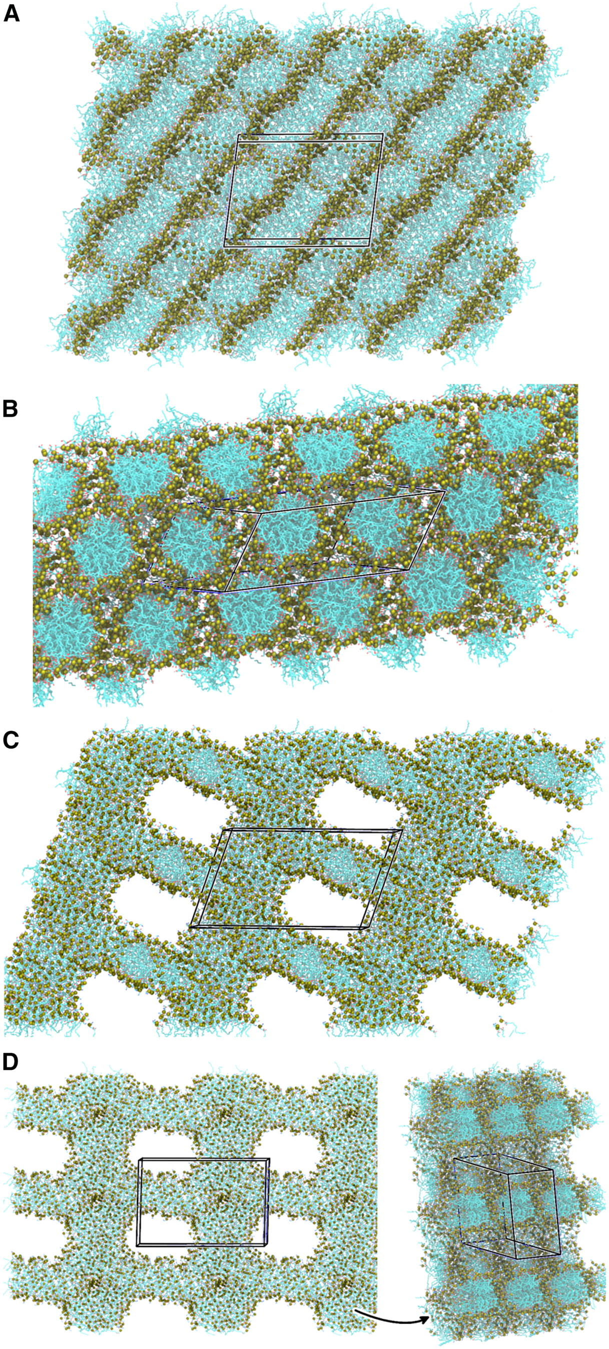

FIGURE 6 Final phases formed by the modeled lipids at low hydration under anisotropic pressure coupling. Unit cells are indicated by a black box. Lipids are colored by element in stick representation, with headgroup phosphorus atoms shown as tan spheres. Water and ions are omitted for clarity. (*A*) $C_W = 0.1$. LysoPE$_{(18:1)}$ approaching a multilayered lamellar phase; there are still headgroups within the hydrophobic core of the bilayers. Two replicates each of lysoPE$_{(18:1)}$ and lysoPE$_{(19:0c)}$ reached this phase. (*B–D*) $C_W = 0.4$. (*B*) Hexagonal phase (H_I) formed by two replicates of lysoPG$_{(18:1)}$. (*C*) Mixed phase: combination of a bilayer-like assembly and a cylindrical assembly. (*D*) Cylindrical micelles in two directions, with lipid bridges at intersections.

phospholipid-only systems, each replicate yielded a bilayer. The mechanism of bilayer self-assembly closely followed that described above (Fig. 7). A nonporous bilayer was formed in under 150 ns for two replicas and in ~300 ns in the third. In all cases, these bilayers remained stable and nonporous for the remainder of the simulation (1 μs).

The contents of each leaflet were calculated to assess the level of asymmetry in the self-assembled bilayers (Table 1). Although a strictly symmetric bilayer was not possible owing to an odd number of molecules for most species, the lysophospholipid content of each leaflet was considered. We would expect that the contents of each leaflet should approximately reflect the contents of the whole system (i.e., ~20% LPLs) to minimize asymmetry and therefore curvature stress. For replicates 1 and 3, this was found to be the case, with the lysophospholipid content of each leaflet within 2.1 percentage points of the expected value. However, replicate 2 displayed greater asymmetry, with substantially more LPLs in the lower leaflet (18 versus 10).

Formation of the porated bilayers (Fig. S4) occurred within 40 ns in each case. As lipid diffusion is relatively slow, the initial aggregation of the lipids will influence the final contents of each leaflet; once a stable/metastable bilayer arrangement is achieved, it is unlikely that this will change substantially without additional energetic input. Further to this, no lipid flip-flopping was observed during the simulations; the lipid content of each leaflet was constant once the bilayer had formed in each case. This is expected as the energy barrier to lipid flip-flop events is large (typically > 20 $kJmol^{-1}$ (75,76)) resulting in timescales on the order of hours to weeks (77,78).

We did not observe any large persistent clusters of LPLs within the formed bilayers. Although the lateral distribution of the LPLs in the self-assembled bilayers was not uniform, there was no obvious phase separation or clear enrichment of LPLs that was maintained for long periods of time (Fig. 7; Table S9). However, the stochastic dimerization of LPLs over the leaflets does appear to be important in poration events; this is further discussed in the Electroporation section.

Mixed bilayer properties

The properties of the bilayers containing 20% LPLs were compared with those containing only phospholipids under equilibrium conditions. To increase sampling, larger bilayers were generated (materials and methods). There was no clear propensity for any of the LPLs to form aggregates with either other LPLs or PLs either in the presence or absence of protein (Fig. S5).

Bilayer biophysical properties

The biophysical properties of the bilayers were compared using the following metrics: bilayer thickness, mean curvature, and area compressibility.

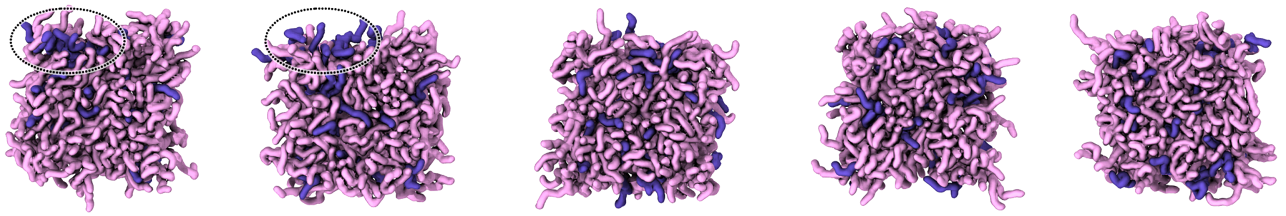

FIGURE 7 Lateral distribution of lysophospholipids in a self-assembled bilayer. Snapshots from replicate 1 of the self-assembled bilayer at different times. Lysophospholipids are shown in purple and phospholipids in pink. Although there is occasionally some aggregation of the LPLs in the bilayer (e.g., t = 300–320 ns, highlighted with a circle), these clusters are not long-lived.

Bilayer thickness was calculated over the final 500 ns of each simulation using LiPyphilic (39–41). Each bilayer was divided into a 10 $\times$ 10 grid and the bilayer thickness calculated in each bin as the distance between phosphorus atoms in the two leaflets. The bilayer thickness returned is the mean across all bins. The phospholipid-only bilayers were found to be slightly thicker than those containing LPLs: the bilayers containing only POPG, POPE, and POPA displayed a mean ($\pm$ standard deviation) thickness of 3.917 $\pm$ 0.025 nm, whereas the 20% LPLs bilayers displayed a thickness of 3.826 $\pm$ 0.027 nm.

The partial densities of different groups within the systems were calculated over the z-axis to visualize these differences. Densities were calculated for lipid phosphorus atoms, acyl tails, glycerol groups, and water molecules over the final 500 ns of each simulation. Segmenting the z-axis into 100 bins, these distributions were similar across the two configurations (Fig. 8). The main difference is a subtle shift toward a thinner bilayer for the LPL-containing systems. For each moiety, there is a small shift in the distributions toward the center of the bilayer ($z = 0$) for the 20% LPLs systems: the hydrophobic core is thinner, and the distance between glycerol/phosphate groups is reduced on inclusion of LPLs. Furthermore, the hydroxyl groups of the LPLs are more hydrophilic than the ester groups that link the headgroup to the acyl tails. The effective size of the polar headgroup is thus increased in a lysophospholipid compared with its diacyl counterpart, which allows water to penetrate deeper into the bilayer than in a pure phospholipid membrane (13).

TABLE 1 Contents of Each Leaflet in the Self-Assembled 20% LPL Bilayers

	R1		R2		R3	
	Upper	Lower	Upper	Lower	Upper	Lower
POPG	32	31	33	30	31	32
POPE	24	18	21	21	18	24
POPA	3	4	5	2	4	3
$\text{LysoPE}_{(18:1)}$	2	5	3	4	4	3
$\text{LysoPE}_{(16:0)}$	4	3	4	3	4	3
$\text{LysoPE}_{(19:0c)}$	3	4	1	6	2	5
$\text{LysoPG}_{(18:1)}$	4	3	2	5	5	2
% LPL	18.1	22.1	14.5	25.4	22.1	18.1

The bilayer thickness and mean leaflet curvature for each leaflet were compared for bilayers containing only PL and those with 20% LPL content (Figs. S6 and S7). Aside from the aforementioned overall thinning when LPL are present, we did not observe any particular trends in terms of mean curvature being impacted by the presence of the LPLs; the mean curvatures are similar whether LPL is present/absent.

To further characterize the differences in mechanical properties of the bilayer with lysophospholipids, we have computed the area compressibility modulus K_A (Table S10). Although Rice et al. (79) have reported a reduction in the K_A as lysophospholipid content of PL bilayers is increased from comparable MD simulations, here, we do not see a similar overall reduction across all three replicas, although the two lowest values of K_A are from the LPL-containing bilayers. We note that the lowest LPL content in the bilayers reported by Rice et al. was 50%, whereas ours contain only 20% LPL, and thus the effect on area compressibility is not expected to be as appreciable.

Lipid diffusion

Previous studies have shown that the addition of LPLs to egg phosphatidylcholine (16:0, 18:0 tails) bilayers can change membrane fluidity (80). Addition of $\text{lysoPC}_{(16:0)}$ significantly increased diffusion, whereas addition of $\text{lysoPE}_{(16:0)}$ slightly decreased diffusion (80). A combination of factors was cited, including changes in van der Waals interactions; lipid packing; hydrogen bonding between phosphate groups and differing headgroups; and changes in bilayer thickness due to different tail lengths (80). Owing to the complex mixture of lipids investigated here, it is difficult to predict how we would expect the diffusion of each lipid in the bilayer to change with composition.

The lateral diffusion constant for each lipid type in each bilayer was calculated over the final 500 ns of each simulation using the GROMACS utility msd (32,33). These values are presented in Table 2. The average value for the phospholipid diffusion coefficients appears to decrease slightly when LPLs are included. However, the relatively large standard

TABLE 2 Diffusion Coefficients for the Lipids in Each Bilayer System

	$D_{xy}/1 \times 10^{-7}$ cm^2 s^{-1}	
	Phospholipid Only	20% LPL Mix
POPG	1.32 ± 0.07	1.20 ± 0.25
POPE	1.23 ± 0.14	1.16 ± 0.15
POPA	1.36 ± 0.38	1.24 ± 0.25
$LysoPE_{(18:1)}$		1.29 ± 0.30
$LysoPE_{(16:0)}$		1.33 ± 0.69
$LysoPE_{(19:0c)}$		1.46 ± 0.36
$LysoPG_{(18:1)}$		1.34 ± 0.40

Each value is presented as the mean ± standard error of the mean across the three replicates of each system.

errors mean that the confidence in these values being representative of the true mean is low. We might expect a given lysophospholipid to display greater mobility compared with its diacyl counterpart (13) (e.g., lysoPE versus POPE). However, the standard error in these values again precludes any significant conclusions.

Area per lipid

The area per lipid (APL) was calculated over the final 500 ns of each trajectory for each system in two different ways. The average APL across all lipids in both leaflets was calculated by dividing the total area (twice the area of the *xy* plane) by the total number of lipids. The species-specific APL was then calculated using 2D Voronoi tessellation in LiPyphilic (41,81,82). The calculated areas are presented in Table 3.

Three main observations were made from these values: the APL for a given lysophospholipid is smaller than that for its diacyl counterpart; the average APL across all lipids is reduced in bilayers containing the LPLs; and the APL for POPG, POPE, and POPA decreases on inclusion of LPLs. As the LPLs possess only one acyl tail, they occupy less space in the *xy* plane, leading to a reduced APL compared with the equivalent two-tailed lipid. Further to this, the LPLs appear to encourage closer packing of the phospholipids. If each phospholipid continued to occupy the same area as in a purely phospholipid bilayer, the reduced volume of the hydrophobic tails of the LPLs could lead to small vacuums in the regions around the LPLs tails. Thus, the lipids occupy less space in order to maintain the hydrophobic core of the bilayer.

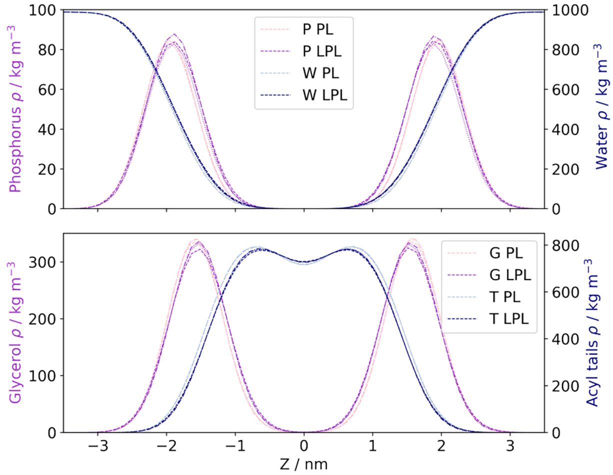

FIGURE 8 Partial densities of lipid moieties and water molecules in equilibrium simulations of the model bilayers. PL indicates phospholipid-only systems; LPL indicates 20% LPL systems. Top: lipid phosphorus atoms and water molecules. Bottom: lipid glycerol ester groups and lipid acyl tails. All partial densities are consistent with a subtle thinning of the bilayer on addition of LPLs.

Tail order parameters

The tail order parameter S_{CD} was calculated separately for each phospholipid (POPG, POPE, POPA) acyl chain across all lipids of the same species over the final 100 ns of each replicate. All three phospholipids display a small decrease in order on inclusion of LPLs (Fig. 9). As discussed in the previous section, the packing of the lipids in the bilayer changes on addition of LPLs to maintain the hydrophobic core: the APL is reduced in order to maximize hydrophobic interactions between the acyl tails. However, due to the conical shape of LPLs, there will still be an increased amount of free space within the core for the mixed bilayers compared with those containing only phospholipids. As a result, the phospholipid tails exhibit greater mobility and thus slightly reduced order parameters in the mixed bilayer systems.

Native protein embedded in mixed bilayer

We further tested the mixed membrane model by embedding the protein PglB in a preequilibrated bilayer. PglB is a native inner membrane protein in *C. jejuni* with oligosaccharyltransferase activity, which is needed for the transfer of glycans to acceptor proteins. This protein itself is glycosylated,

TABLE 3 Area per Lipid (APL) for the Lipids in Each Bilayer System

	Area per Lipid/nm^2		
	Phospholipid Only	20% LPL Mix	ΔAPL/%
All	0.6358 ± 0.0001	0.5977 ± 0.0001	5.99
POPG	0.6778 ± 0.0003	0.6471 ± 0.0003	4.53
POPE	0.6119 ± 0.0005	0.5797 ± 0.0004	5.26
POPA	0.6153 ± 0.0014	0.5761 ± 0.0011	6.37
$LysoPE_{(18:1)}$		0.5639 ± 0.0011	
$LysoPE_{(16:0)}$		0.5542 ± 0.0011	
$LysoPE_{(19:0c)}$		0.5636 ± 0.0011	
$LysoPG_{(18:1)}$		0.6220 ± 0.0012	

Each value is presented as the mean ± standard error of the mean across the three replicates of each system. The first row indicates the average APL across all lipids, calculated using the total area of the two leaflets divided by the total number of lipids. Species-specific values were calculated using 2D Voronoi tessellation of atomic positions in LiPyphilic. The final column presents the percentage change between the phospholipid only system and the phospholipid-LPL system.

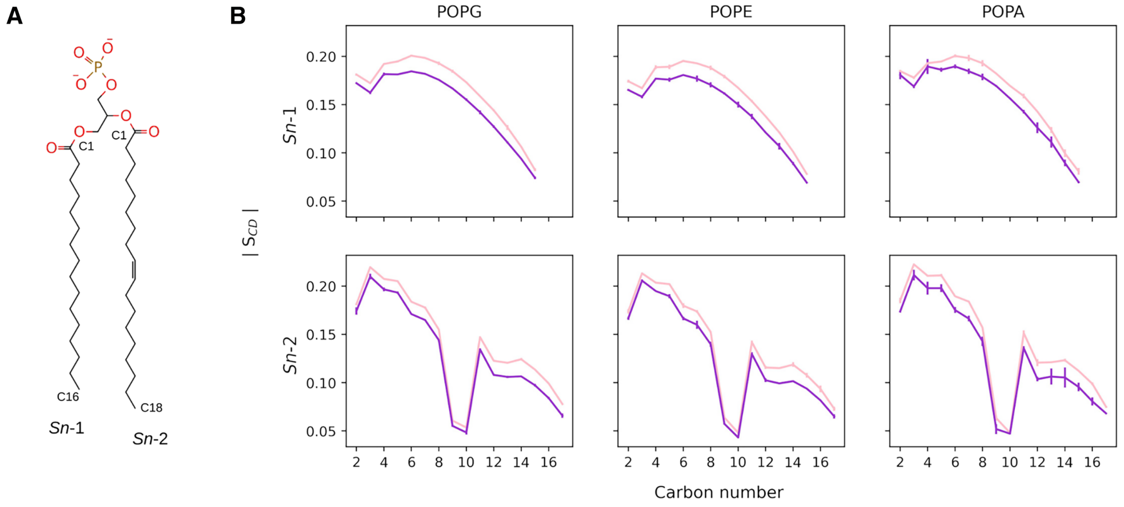

FIGURE 9 Comparison of tail order parameters for the phospholipids in different bilayer environments. (*A*) Structure of the phospholipid POPA with the *Sn*-1 and *Sn*-2 tails labeled, with start and end carbon atoms labeled on each chain. POPE and POPG share this tail structure. (*B*) Magnitude of the order parameter S_{CD} for each carbon in each tail. Pink line indicates the average over replicates containing only phospholipids; purple line indicates 20% LPLs systems. Error bars show the standard deviation across the three replicates of each system. In all cases, the presence of LPLs reduces the order of the tails.

with the acceptor sequon in the periplasmic domain. Three replicas of a system containing the mixed bilayer and one glycosylated PglB with its acceptor peptide and donor LLO were simulated for 1 μs each.

Membrane properties

Overall, the bilayer maintained its stability in all replicas, even though a slight, principally negative, mean local mean curvature was induced around the protein (Figs. S8 and S9). Lipid tail order parameters computed for each phospholipid type were consistent among all replicas (Fig. 10 *A*) and directly comparable with the order parameters reported for the mixed bilayer alone (Fig. 9).

For all replicas, the mean thickness values (± standard deviation) computed over the last 500 ns spanned values between 3.806 and 3.813 ± 0.041 nm. These values are comparable with the ones previously mentioned for a bilayer-only system (3.826 ± 0.027). The results show that the total average APL is consistent between the replicas (spanning values between 0.670 and 0.673 ± 0.001 nm^2; Table 4). The computed local APL showed slightly more variation between replicas, particularly so for the LPLs, as shown in Table 4. Perhaps, the most noticeable cases are lysoPE$_{(18:1)}$ and lysoPG$_{(18:1)}$, which show a difference in APL of around 0.10 nm^2 between replica 1 (R1) and 2–3. Toward the end of one of the trajectories, (R1) one lysoPG$_{(18:1)}$ molecule moved such that its headgroup was well outside the plane of the headgroups of the other lipids, where it appeared to be stabilized by interactions with asparagine residues on the surface of PglB (Fig. S10). This event only occurred in one simulation, and thus the significance is unclear.

Protein and glycan dynamics

Overall, PglB remained stably embedded inside the membrane for all replicas (Fig. S9). The secondary structure elements of the protein remained mainly stable along the simulation, as shown in Fig. 10 *C*. Some dynamical behavior can be observed around the region spanning residues 290–300, a mainly unstructured region that is highly mobile and whose structural prediction, as reported in the AlphaFold model present in Uniprot, have low to very low confidence score (including a section with pLDDT <50). The root mean-square deviation (RMSD) of the PglB backbone over time shows that the overall conformation of the protein is still changing slightly (particularly for replica R3), but in all cases, it reached values around 0.4 nm by the end of the simulation. This could be rationalized by considering a local onformational change around the region binding the acceptor sequon peptide. In two replicas (R2 and R3), this peptide spontaneously dissociated from its binding site. The residue-based root mean-square fluctuation was also computed and is shown in Fig. 10 *E*. All three replicas show the same qualitative profile indicating similar local dynamical behavior. In the case of R3, although a new peak appears in the region spanning residues 250–270, which corresponds to regions spanning two distal transmembrane helices, overall, the RMSF profile is qualitatively comparable with the other simulated systems. Finally, we assessed the interactions of the glycans with the bilayer. Although PglB is glycosylated, this analysis was carried out on the carbohydrate elements of the glycosylated lipid donor, since it is the only molecule in this system with its glycan moiety proximal to the membrane. The sugar-lipid contacts were computed considering the lipids separately

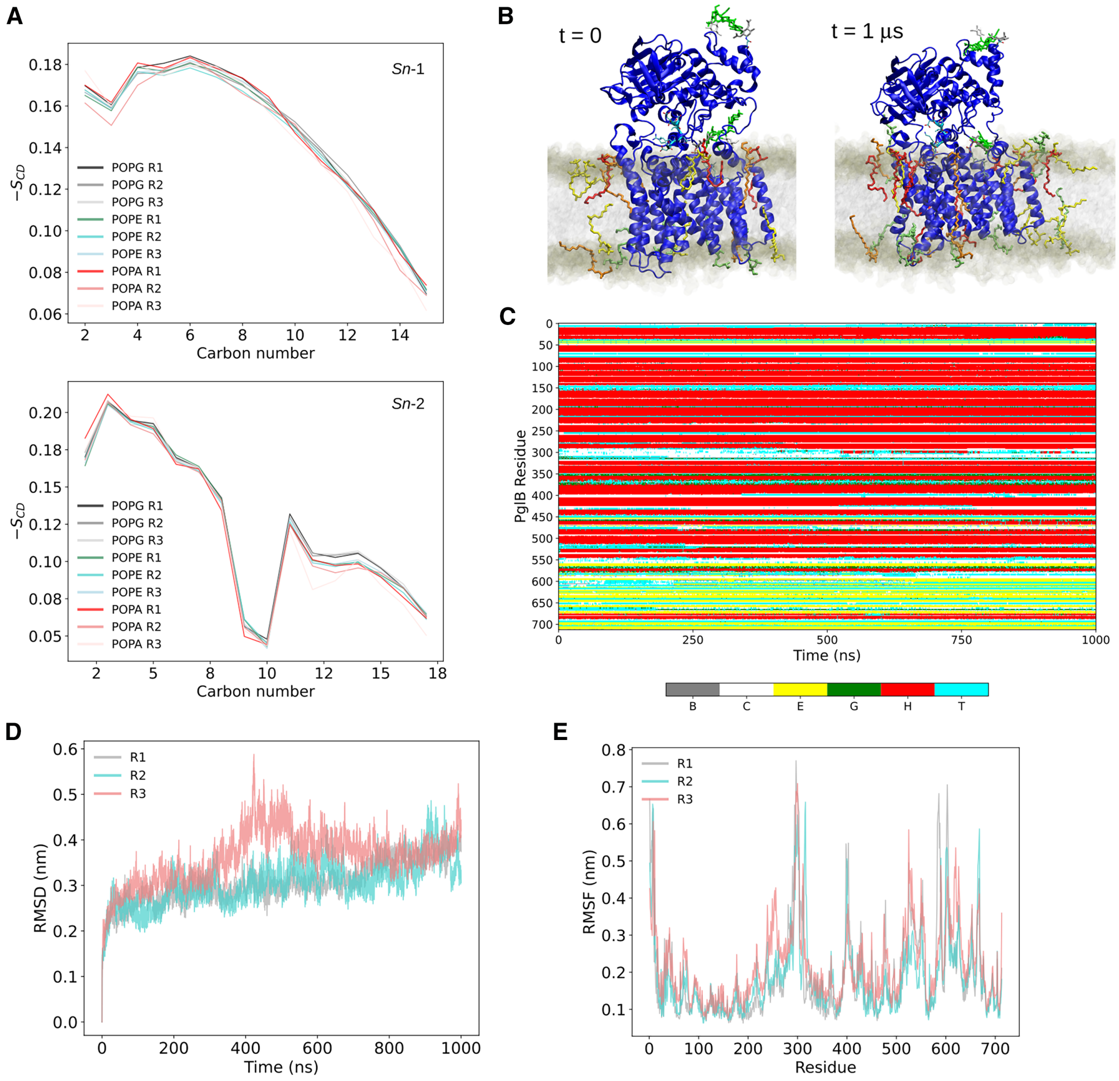

FIGURE 10 Properties of membrane-protein systems. (*A*) Deuterium tail order parameters computed separately for all the phospholipids in each replica (R1–R3). (*B*) Zoomed-in side view of the system at the beginning and end of the production run for one example simulation (R1). The membrane is depicted as a translucent gray surface with phosphorus atoms of the lipids colored in brown. PglB is shown as a blue cartoon representation. The acceptor sequon is shown with a cyan cartoon representation. Sugar components are shown by residue-based colored sticks (*white, gray, green*). LPLs that are in contact with PglB are depicted with residue-based colored sticks inside the bilayer (lysoPE$_{(18:1)}$: *red*, lysoPE$_{(16:0)}$). (*C*) Secondary structure analysis for PglB over time in a representative replica (R1). STRIDE was used to compute the following secondary structure elements: isolated bridge (B, *gray*), coil (C, *white*), extended conformation (E, *yellow*), 3–10 helix (G, *green*), alpha-helix (H, *red*), turn (T, *cyan*). (*D*) Root mean-square deviation (RMSD) computed over the production run for all three replicas (R1–R3). (*E*) Root mean-square fluctuation (RMSF) of PglB computed on the production run for all three replicas.

(see materials and methods for more details). A dynamic behavior was observed, where in all cases contacts could be established and lost in an unbiased way (Fig. S11; Table S11).

Electroporation

Application of an electric field perpendicular to the bilayer has been explored extensively via MD as a method to induce pores (83–89)—a process known as electroporation. The mechanism of electroporation begins with the formation of a water wire through the bilayer, favoring locations with local defects in the headgroup region (85,86). The water wire is stabilized through interactions with lipid headgroups, which move into the hydrophobic core to further stabilize the water within the bilayer (86,87). This allows the water wire to expand, resulting in pores in the membrane. In simulations, this will often cause the rapid acceleration of water molecules into the channel and expansion of the pore in the bilayer plane. As the presence of varied lipid types results in a greater number of defects in the bilayer due to changes in the hydrophobic packing of the tails and hydrogen bonding between headgroups, we hypothesized that the presence of LPLs will allow

TABLE 4 Area per Lipid (APL) for the Lipids in Each Protein-Bilayer System

	Area per Lipid/nm^2		
	R1	R2	R3
All	0.673 ± 0.001	0.671 ± 0.001	0.670 ± 0.001
POPG	0.715 ± 0.002	0.713 ± 0.002	0.722 ± 0.002
POPE	0.612 ± 0.001	0.641 ± 0.002	0.601 ± 0.001
POPA	0.621 ± 0.004	0.601 ± 0.004	0.692 ± 0.005
LysoPE$_{(18:1)}$	0.698 ± 0.007	0.593 ± 0.004	0.685 ± 0.008
LysoPE$_{(16:0)}$	0.633 ± 0.006	0.583 ± 0.004	0.581 ± 0.003
LysoPE$_{(19:0c)}$	0.590 ± 0.003	0.658 ± 0.007	0.643 ± 0.005
LysoPG$_{(18:1)}$	0.831 ± 0.007	0.719 ± 0.005	0.714 ± 0.006

Each value is presented as the mean ± standard error of the mean across the last 500 ns of each simulated system. Species-specific values were calculated using 2D Voronoi tessellation of phosphorus atomic positions of the lipids as implemented in fatslim (70).

electroporation at lower field strengths. To compare the integrity of the bilayers containing the lysophospholipids versus those without, we subjected these bilayers to an electric field normal to the membrane plane.

Equilibrated bilayers containing either 20% LPLs or exclusively phospholipids were subjected to a constant electric field along the z-axis, with field strengths from 0.1 to 0.2 V nm^{-1}. The time taken for the bilayer to electroporate was then measured as the time at which the box x (equivalent to y) dimension increases to >10% greater than the equilibrium box dimensions (Fig. S12) (i.e., the bilayer is expanding in the xy plane to accommodate a large pore that has formed in the membrane).

The electroporation times indicate that bilayers containing LPLs are more susceptible to electroporation than those containing only phospholipids (Table 5). Phospholipid-only bilayers were resistant to electroporation on the simulated timescales up to field strengths of 0.200 V nm^{-1}, whereas bilayers containing LPLs were susceptible to electroporation at 0.150 V nm^{-1} and could not withstand field strengths greater than this. This increase in susceptibility to electroporation is consistent with the increase in tail disorder. Due to the stochastic nature of the membrane defects that lead to electroporation, the time taken for the membranes to electroporate is variable across replicates. The pores were found to close when the porated bilayers were subjected to equilibrium conditions. The time taken for the pores to close was measured as the time at which all water had retreated from the hydrophobic core. On average, these pores closed faster in the phospholipid-only systems (Table 5).

LysoPC has been shown experimentally to reduce electrical resistance and increase permeability of phosphatidylcholine bilayers (20,90). The lysophospholipids in those bilayers are hypothesized to spontaneously form ion channels in the bilayer wherein two pairs of dimerized LPLs (each pair containing one lysophospholipid from each leaflet) stabilize a channel through the membrane (20,90). In our simulations, some pores formed in the bilayers containing 20% LPLs appear to support this mechanism (Fig. 11). Initial water wires form at positions where there are proximal LPLs in both leaflets, with the headgroups of these LPLs (and other nearby phospholipids) moving toward the bilayer center to stabilize the pore. Even though some local enrichment of LPLs could be observed in the vicinity of the pores or the initial water wire, this behavior is not observed across all replicas, and consequently, we are not claiming this as a general mechanistic point (Fig. S13; Table S12).

Limitations

It is useful to reflect on any potential limitations of the current study. Perhaps the most important, although we have qualitatively evaluated these models against general properties of this lipid family, the absence of quantitative data on the physical properties of these specific lipids in the literature has precluded quantitative assessment.

Our simulations in which phase behavior was tested were somewhat limited by the system size and pressure coupling regimes. Small system sizes lead to difficulties in reproducing bulk properties as there is a requirement for periodic replication of the unit cell. Similarly, only the use of anisotropic coupling can allow the box to deform in such a way that accessible phases are not limited by box dimensions and enforcement of equal deformation in all dimensions. However, it can also lead to enormous deformation, to the point where the box collapses; this was observed in several simulations.

The electric field strengths utilized in the electroporation simulations are an order of magnitude greater than those employed in experimental studies (83), as is usual for MD simulations given the much shorter timescales compared with laboratory experiments. The field strengths applied here are consistent with other MD studies (83,84,91,92). Previous studies have also shown that the probability of observing poration increases with bilayer patch size (91). Although we attempted to generate bilayers of comparable

TABLE 5 Electroporation Times for the Modeled Bilayers Under Different Field Strengths and Pore Closer Times Under Equilibrium Conditions

	Poration Time/ns					
Field Strength	20% LPL			Phospholipids Only		
V nm^{-1}	R1	R2	R3	R1	R2	R3
0.100	–	–	–	–	–	–
0.125	–	–	–	–	–	–
0.150	11.1	–	14.7	–	–	–
0.175	69.6	73.6	41.9	–	–	–
0.200	20.4	11.2	7.8	31.0	17.5	5.5
	**Pore closure time**/ns					
–	66.9	10.2	76.5	8.4	7.8	15.3

Poration time measured as the time at which there is a large pore in the bilayer, resulting in the x dimension increasing by >10% compared with the equilibrium box dimensions. Pore closure time measured as the time at which all water had retreated from the hydrophobic core.

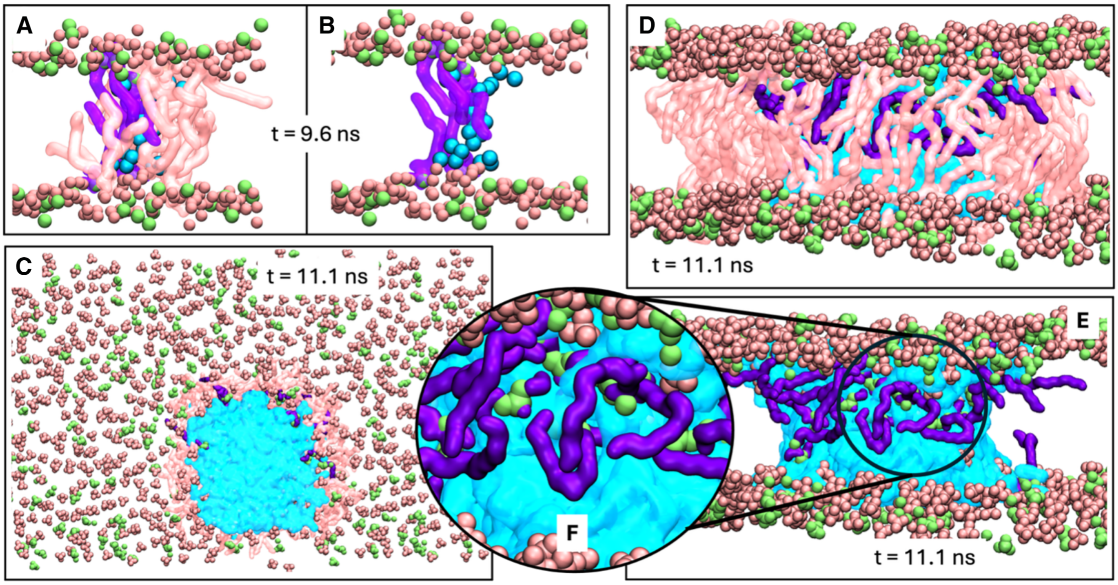

FIGURE 11 Electroporation of a 20% LPL bilayer (replicate 1, field strength 0.15 V nm^{-1}). POPG, POPE, and POPA are in light pink and LPLs in purple. The phosphorus atoms of the PL are depicted as pink spheres; for the LPLs, the full phosphate groups are shown as lime spheres; only lipids in contact with the water in a pore are shown as sticks. Oxygen atoms from water molecules producing a pore are shown in cyan. (*A*) Side view of the first full water wire instance observed (t = 9.6 ns). (*B*) Same as (*A*), but with the PL removed for clarity. (*C*) Top-down view of the bilayer at the reported pore formation time in Table 5 (t = 11.1 ns), with water shown as cyan surface. (*D*) Zoomed-in side-view from panel (*C*). (*E*) shows the LPL lipids that line one side of the pore, with a zoomed-in view in (*F*), in which the reoriented LPLs are clearly seen, and the lime-colored phosphate groups are in contact with the water in the core of the bilayer.

size for these simulations, the phospholipid-only bilayers were slightly larger than their 20% LPL counterparts (*x* dimensions of 13.5 nm versus ~13.0 nm, respectively; ~4% larger). Whether this small difference in size influenced the field strength required for electroporation was not investigated here. However, the phospholipid-only bilayers were the larger of the two configurations yet required greater field strengths to electroporate: the notion that LPLs reduce the integrity of the bilayer holds.

Finally, the complexity of the bilayers generated still falls short of the complete *C. jejuni* lipidome, which has been shown to contain more than 200 lipid species (9). Here, we have selected four lysophospholipids to model, and our bilayers contain seven lipid species in total; the tail and headgroup diversity in vivo is substantially greater than that modeled. Notable species that we have omitted include lysophospholipids with myristoyl tails (C_{14}), which have recently been highlighted as a novel virulence factor for *C. jejuni* (93), and acylphospholipids and phospholipids containing cyclic moieties in their tails (9). The latter species have greater acyl tail volume, which can complement the lysophospholipids, including acylphospholipids, and a greater proportion of lipids with cyclic moieties may be necessary to accommodate higher levels of LPLs (9) in model membranes.

CONCLUSIONS

In this work, we have developed and tested atomistic models for four lysophospholipids found in the *C. jejuni* lipidome. Our work builds upon previous simulation work, both atomistic (79,94) and coarse grained (95) of other single-tailed lysophospholipids from other bacterial species.

We have shown that our atomistic models qualitatively match the expected behaviors of this family of lipids: micelles are formed at low lipid concentration, but at higher concentrations, hexagonal, lamellar, and cubic phases may form. It was shown that solutions of a phospholipid-lysophospholipid mixture could self-assemble into bilayers on the submicrosecond timescale. These bilayers were found to be slightly thinner than their phospholipid-only counterparts. Thinning of a model *H. pylori.* membrane upon addition of LPLs was also reported by Rice et al., with both 16-carbon and 14-carbon LPS tails (94).

Although we did not observe any significant changes in lipid diffusion across the two bilayer compositions, the presence of LPLs was shown to reduce the APL and tail order parameters for each phospholipid species in the bilayer. We have shown that a glycosylated native inner membrane protein is conformationally stable within the mixed lipid bilayer and does not disrupt the membrane environment. Furthermore, these mixed membranes displayed increased susceptibility to electroporation compared with bilayers without LPLs, consistent with the LPLs increasing the permeability of bilayers (18–20) and imparting greater flexibility, fluidity, and susceptibility to poration from other simulation studies (79,94). One of the most biochemically intriguing aspects of the *C. jejuni* cell envelope is the presence of glycosylated proteins in both membranes. Our

simulations of the N-glycosylated protein PglB enabled characterization of its conformational dynamics and revealed dynamic protein-lipid interactions, and on the timescales simulated here, no lipid preference was observed for glycan interactions. Overall, we have provided parameter sets for four LPLs that enable more thorough and biological relevant modeling of the *C. jenuni* lipidome than has previously been possible. We have used these parameters to characterize the phase behavior of each of the four lipids individually, and the impact of all four together on the biophysical properties of the inner membrane of *C. jejuni*, and we found the latter to be similar to the effect of the presence of LPS in *H. pylori* (94). We have shown that model *C. jejuni* inner membranes containing our lipid parameter sets are suitable for simulations of complex membrane proteins such as the glycosylated PglB, and given that the Pgl system plays key roles in several phenotypes including motility, biofilm formation, and resistance to antibiotics (96,97), our data represent a first step and benchmark for future work that explores PglB and other proteins in the PgL system in greater depth. We note here that coarse-grained models would be beneficial to enable longer and larger simulations of the inner membrane that incorporate multiple proteins in future work to study, for example, the mechanism of action of antibiotics, but we appreciate the challenges for developing such models (98).

ACKNOWLEDGMENTS

K.E.N. is supported by EPSRC (PhD Studentship Project Number: 2446840). A.F.B. is funded by EP/V030779/1. S.K. is funded by EPSRC grants EP/X035603, EP/V030779/1, and EP/Y008693/2. J.W.E. is funded by EP/Y008693/2. This work used the ARCHER2 UK National Supercomputing Service. The authors acknowledge the use of the IRIDIS High Performance Computing Facility, and associated support services at the University of Southampton, in the completion of this work.

AUTHOR CONTRIBUTIONS

S.K. designed the research, K.E.N. and A.F.B. performed the research, and A.F.B. and K.E.N. analyzed the data. All authors contributed to the writing of the manuscript.

DECLARATION OF INTERESTS

The authors declare no competing interests.

SUPPORTING MATERIAL

Supporting Material can be found online at https://doi.org/10.1016/j.bpj.2025.08.024.

REFERENCES

1. Cronan, J. E. 2003. Bacterial Membrane Lipids: Where Do We Stand? *Annu. Rev. Microbiol.* 57:203–224.
2. Zheng, L., Y. Lin, …, M. Bogdanov. 2017. Transport and Remodeling of Lysophospholipids in Gram-Negative Bacteria. *Biochim. Biophys. Acta. Mol. Cell Biol. Lipids.* 1862:1404–1413.
3. Bishop, R. E., H. S. Gibbons, …, C. R. Raetz. 2000. Transfer of Palmitate from Phospholipids to Lipid A in Outer Membranes of Gram-Negative Bacteria. *EMBO J.* 19:5071–5080.
4. Weiss, J. P. 2015. Molecular Determinants of Bacterial Sensitivity and Resistance to Mammalian Group IIA Phospholipase A2. *Biochim. Biophys. Acta.* 1848:3072–3077.
5. Jackowski, S., and C. O. Rock. 1986. Transfer of Fatty Acids from the 1-Position of Phosphatidylethanolamine to the Major Outer Membrane Lipoprotein of Escherichia Coli. *J. Biol. Chem.* 261:11328–11333.
6. Cao, X., J. P. M. van Putten, and M. M. S. M. Wösten. 2023. Biological Functions of Bacterial Lysophospholipids. *Adv. Microb. Physiol.* 82:129–154.
7. Zou, D., J. Pei, …, Z. Lin. 2020. A SNP of Bacterial Blc Disturbs Gut Lysophospholipid Homeostasis and Induces Inflammation through Epithelial Barrier Disruption. *EBioMedicine.* 52:102652.
8. Casares, D., P. V. Escribá, and C. A. Rosselló. 2019. Membrane Lipid Composition: Effect on Membrane and Organelle Structure, Function and Compartmentalization and Therapeutic Avenues. *Int. J. Mol. Sci.* 20:2167.
9. Cao, X., J. F. H. M. Brouwers, …, M. M. S. M. Wösten. 2020. The Unique Phospholipidome of the Enteric Pathogen Campylobacter Jejuni: Lysophosholipids Are Required for Motility at Low Oxygen Availability. *J. Mol. Biol.* 432:5244–5258. https://doi.org/10.1016/j.jmb.2020.07.012.
10. Bogdanov, M., M. Umeda, and W. Dowhan. 1999. Phospholipid-Assisted Refolding of an Integral Membrane Protein: Minimum Structural Features for Phosphatidylethanolamine to Act as a Molecular Chaperone. *J. Biol. Chem.* 274:12339–12345.
11. Lin, Y., M. Bogdanov, …, L. Zheng. 2016. Substrate Selectivity of Lysophospholipid Transporter LplT Involved in Membrane Phospholipid Remodeling in Escherichia Coli. *J. Biol. Chem.* 291:2136–2149.
12. Israelachvili, J. N., D. J. Mitchell, and B. W. Ninham. 1976. Theory of Self-Assembly of Hydrocarbon Amphiphiles into Micelles and Bilayers. *J. Chem. Soc., Faraday Trans. 2.* 72:1525–1568.
13. Stafford, R. E., and E. A. Dennis. 1987. Lysophospholipids as Biosurfactants. *Colloids Surf.* 30:47–64.
14. Slater, J. L., C.-H. Huang, …, I. W. Levin. 1989. Polymorphic Phase Behavior of Lysophosphatidylethanolamine Dispersions. A Thermodynamic and Spectroscopic Characterization. *Biophys. J.* 56:243–252.
15. Marsh, D. 2013. Handbook of Lipid Bilayers. CRC Press.
16. Fuller, N., and R. P. Rand. 2001. The Influence of Lysolipids on the Spontaneous Curvature and Bending Elasticity of Phospholipid Membranes. *Biophys. J.* 81:243–254. https://doi.org/10.1016/S0006-3495(01)75695-0.
17. Michel, G. 1979. The Changes in Cell Size and Phospholipid Composition during Growth of a Chain-Forming EnvC Mutant of Escherichia Coli. *FEBS Lett.* 100:258–260.
18. Henriksen, J. R., T. L. Andresen, …, J. H. Ipsen. 2010. Understanding Detergent Effects on Lipid Membranes: A Model Study of Lysolipids. *Biophys. J.* 98:2199–2205.
19. Yaginuma, S., J. Omi, …, J. Aoki. 2023. Lysophospholipids and Their Producing Enzymes: Their Pathological Roles and Potential as Pathological Biomarkers. *Pharmacol. Ther.* 246:108415.
20. Lee, Y., and S. I. Chan. 1977. Effect of Lysolecithin on the Structure and Permeability of Lecithin Bilayer Vesicles. *Biochemistry.* 16:1303–1309.
21. Sasaki, H., H. Arai, …, S. H. White. 2009. Dependence of Sphingosine Aggregation. *Biophys. J.* 96:2727–2733.
22. Arvidson, G., I. Brentel, …, K. Fontell. 1985. Phase Equilibria in Four Lysophosphatidylcholine/Water Systems: Exceptional Behaviour of 1-Palmitoyl-Glycerophosphocholine. *Eur. J. Biochem.* 152:753–759.
23. Newman, K. E., and S. Khalid. 2023. Conformational Dynamics and Putative Substrate Extrusion Pathways of the N-Glycosylated Outer

Membrane Factor CmeC from Campylobacter Jejuni. *PLoS Comput. Biol.* 19:e1010841.

24. Parrill, A. L. 2008. Lysophospholipid Interactions with Protein Targets. *Biochim. Biophys. Acta.* 1781:540–546.

25. Li, Y.-F., R.-S. Li, …, X.-F. Yang. 2016. Lysophospholipids and Their G Protein-Coupled Receptors in Atherosclerosis. *Front. Biosci.* 21:70–88.

26. Wang, L.-P., J. Chen, and T. Van Voorhis. 2013. Systematic Parametrization of Polarizable Force Fields from Quantum Chemistry Data. *J. Chem. Theory Comput.* 9:452–460.

27. Mallajosyula, S. S., S. Jo, …, A. D. MacKerell. 2015. Molecular Dynamics Simulations of Glycoproteins Using CHARMM. *Methods Mol. Biol.* 1273:407–429.

28. Huang, J., and A. D. MacKerell. 2013. CHARMM36 All-Atom Additive Protein Force Field: Validation Based on Comparison to NMR Data. *J. Comput. Chem.* 34:2135–2145. https://doi.org/10.1002/jcc.23354.

29. Huang, J., S. Rauscher, …, A. D. MacKerell. 2017. CHARMM36m: An Improved Force Field for Folded and Intrinsically Disordered Proteins. *Nat. Methods.* 14:71–73. https://doi.org/10.1038/nmeth.4067.

30. Klauda, J. B., R. M. Venable, …, R. W. Pastor. 2010. Update of the CHARMM All-Atom Additive Force Field for Lipids: Validation on Six Lipid Types. *J. Phys. Chem. B.* 114:7830–7843. https://doi.org/10.1021/jp101759q.

31. Jorgensen, W. L., J. Chandrasekhar, …, M. L. Klein. 1983. Comparison of Simple Potential Functions for Simulating Liquid Water. *J. Chem. Phys.* 79:926–935. https://doi.org/10.1063/1.445869.

32. Berendsen, H. J. C., D. van der Spoel, and R. van Drunen. 1995. GROMACS: A Message-Passing Parallel Molecular Dynamics Implementation. *Comput. Phys. Commun.* 91:43–56.

33. Van Der Spoel, D., E. Lindahl, …, H. J. C. Berendsen. 2005. GROMACS: Fast, Flexible, and Free. *J. Comput. Chem.* 26:1701–1718.

34. Hess, B., H. Bekker, …, J. G. E. M. Fraaije. 1997. LINCS: A Linear Constraint Solver for Molecular Simulations. *J. Comput. Chem.* 18:1463–1472.

35. Essmann, U., L. Perera, …, L. G. Pedersen. 1995. A Smooth Particle Mesh {Ewald} Method. *J. Chem. Phys.* 103:8577–8593. https://doi.org/10.1063/1.470117.

36. Bussi, G., D. Donadio, and M. Parrinello. 2007. Canonical Sampling through Velocity Rescaling. *J. Chem. Phys.* 126:014101. https://doi.org/10.1063/1.2408420.

37. Harris, C. R., K. J. Millman, …, T. E. Oliphant. 2020. Array Programming with NumPy. *Nature.* 585:357–362. https://doi.org/10.1038/s41586-020-2649-2.

38. McKinney, W. 2010. Pandas: A Foundational Python Library for Data Analysis and Statistics. *In* Proceedings of the 9th Python in Science Conference, pp. 57–61.

39. Gowers, R., M. Linke, O. Beckstein…, 2016. MDAnalysis: a Python package for the rapid analysis of molecular dynamics simulations. In Proceedings of the 15th Python in Science Conference. SCIPY, pp. 98–105. https://doi.org/10.25080/Majora-629e541a-00e.

40. Michaud-Agrawal, N., E. J. Denning, …, O. Beckstein. 2011. MDAnalysis: A Toolkit for the Analysis of Molecular Dynamics Simulations. *J. Comput. Chem.* 32:2319–2327.

41. Smith, P., and C. D. Lorenz. 2021. LiPyphilic: A Python Toolkit for the Analysis of Lipid Membrane Simulations. *J. Chem. Theory Comput.* 17:5907–5919. https://doi.org/10.1021/acs.jctc.1c00447.

42. Humphrey, W., A. Dalke, and K. Schulten. 1996. VMD: Visual Molecular Dynamics. *J. Mol. Graph.* 14:33-8–27-8. https://doi.org/10.1016/0263-7855(96)00018-5.

43. Baptista, A. M., L. da Rocha, and S. R. R. Campos. 2022. FixBox: A General Algorithm to Fix Molecular Systems in Periodic Boxes. *J. Chem. Inf. Model.* 62:4435–4447.

44. Pandit, K. R., and J. B. Klauda. 2012. Membrane Models of E. Coli Containing Cyclic Moieties in the Aliphatic Lipid Chain. *Biochim. Biophys. Acta.* 1818:1205–1210. https://doi.org/10.1016/j.bbamem.2012.01.009.

45. Goldstein, A. A. 1965. On Steepest Descent. *J. Soc. Ind. Appl. Math. Control.* 3:147–151. https://doi.org/10.1137/0303013.

46. Seelig, J. 1977. Deuterium Magnetic Resonance: Theory and Application to Lipid Membranes. *Q. Rev. Biophys.* 10:353–418.

47. Schindler, H., and J. Seelig. 1975. Deuterium Order Parameters in Relation to Thermodynamic Properties of a Phospholipid Bilayer. Statistical Mechanical Interpretation. *Biochemistry.* 14:2283–2287.

48. Piggot, T. J., Á. Piñeiro, and S. Khalid. 2012. Molecular Dynamics Simulations of Phosphatidylcholine Membranes: A Comparative Force Field Study. *J. Chem. Theory Comput.* 8:4593–4609.

49. Vermeer, L. S., B. L. De Groot, …, J. Czaplicki. 2007. Acyl Chain Order Parameter Profiles in Phospholipid Bilayers: Computation from Molecular Dynamics Simulations and Comparison with 2H NMR Experiments. *Eur. Biophys. J.* 36:919–931. https://doi.org/10.1007/s00249-007-0192-9.

50. Heller, H., M. Schaefer, and K. Schulten. 1993. Molecular Dynamics Simulation of a Bilayer of 200 Lipids in the Gel and in the Liquid Crystal Phase. *J. Phys. Chem.* 97:8343–8360. https://doi.org/10.1021/j100133a034.

51. Piggot, T. J., J. R. Allison, …, J. W. Essex. 2017. On the Calculation of Acyl Chain Order Parameters from Lipid Simulations. *J. Chem. Theory Comput.* 13:5683–5696. https://doi.org/10.1021/acs.jctc.7b00643.

52. Egberts, E., S.-J. Marrink, and H. J. Berendsen. 1994. Molecular Dynamics Simulation of a Phospholipid Membrane. *Eur. Biophys. J.* 22:423–436.

53. NMRLipids/Match. https://doi.org/10.5281/zenodo.1234.

54. Carr, M., and C. E. MacPhee. 2015. Membrainy: A "Smart", Unified Membrane Analysis Tool. *Source Code Biol. Med.* 10:3. https://doi.org/10.1186/s13029-015-0033-7.

55. VMD-L Mailing List. https://www.ks.uiuc.edu/Research/vmd/mailing_list/vmd-l/att-14731/calc_op.tcl.

56. GROMACS Reference Manual. https://manual.gromacs.org/current/reference-manual/index.html.

57. Bochicchio, A., A. F. Brandner, …, R. A. Böckmann. 2020. Spontaneous Membrane Nanodomain Formation in the Absence or Presence of the Neurotransmitter Serotonin. *Front. Cell Dev. Biol.* 8:601145. https://doi.org/10.3389/fcell.2020.601145.

58. Parrinello, M., and A. Rahman. 1981. Polymorphic Transitions in Single Crystals: A New Molecular Dynamics Method. *J. Appl. Phys.* 52:7182–7190. https://doi.org/10.1063/1.328693.

59. Nosé, S., and M. L. Klein. 1983. Constant Pressure Molecular Dynamics for Molecular Systems. *Mol. Phys.* 50:1055–1076. https://doi.org/10.1080/00268978300102851.

60. Berendsen, H. J. C., J. P. M. Postma, …, J. R. Haak. 1984. Molecular Dynamics with Coupling to an External Bath. *J. Chem. Phys.* 81:3684–3690. https://doi.org/10.1063/1.448118.

61. Jo, S., T. Kim, …, W. Im. 2008. CHARMM-GUI: A Web-Based Graphical User Interface for CHARMM. *J. Comput. Chem.* 29:1859–1865. https://doi.org/10.1002/jcc.20945.

62. Lee, J., X. Cheng, …, W. Im. 2016. CHARMM-GUI Input Generator for NAMD, GROMACS, AMBER, OpenMM, and CHARMM/OpenMM Simulations Using the CHARMM36 Additive Force Field. *J. Chem. Theory Comput.* 12:405–413. https://doi.org/10.1021/acs.jctc.5b00935.

63. Lee, J., D. S. Patel, …, W. Im. 2019. CHARMM-GUI Membrane Builder for Complex Biological Membrane Simulations with Glycolipids and Lipoglycans. *J. Chem. Theory Comput.* 15:775–786. https://doi.org/10.1021/acs.jctc.8b01066.

64. Waterhouse, A., M. Bertoni, …, T. Schwede. 2018. SWISS-MODEL: Homology Modelling of Protein Structures and Complexes. *Nucleic Acids Res.* 46:W296–W303. https://doi.org/10.1093/nar/gky427.

65. Napiórkowska, M., J. Boilevin, …, K. P. Locher. 2018. Structure of Bacterial Oligosaccharyltransferase PglB Bound to a Reactive LLO

and an Inhibitory Peptide. *Sci. Rep.* 8:16297. https://doi.org/10.1038/s41598-018-34534-0.

66. Jo, S., X. Cheng, …, W. Im. 2014. CHARMM-GUI PDB Manipulator for Advanced Modeling and Simulations of Proteins Containing Nonstandard Residues. *Adv. Protein Chem. Struct. Biol.* 96:235–265. https://doi.org/10.1016/bs.apcsb.2014.06.002.
67. Park, S.-J., N. Kern, …, W. Im. 2023. CHARMM-GUI PDB Manipulator: Various PDB Structural Modifications for Biomolecular Modeling and Simulation. *J. Mol. Biol.* 435:167995. https://doi.org/10.1016/j.jmb.2023.167995.
68. Schrödinger, L. 2015. The PyMOL Molecular Graphics System.
69. Kim, S., J. Lee, …, W. Im. 2017. CHARMM-GUI Ligand Reader and Modeler for CHARMM Force Field Generation of Small Molecules. *J. Comput. Chem.* 38:1879–1886. https://doi.org/10.1002/jcc.24829.
70. Buchoux, S. 2017. FATSLiM: A Fast and Robust Software to Analyze MD Simulations of Membranes. *Bioinformatics.* 33:133–134. https://doi.org/10.1093/bioinformatics/btw563.
71. Skjevik, Å. A., B. D. Madej, …, I. R. Gould. 2016. Simulation of Lipid Bilayer Self-Assembly Using All-Atom Lipid Force Fields. *Phys. Chem. Chem. Phys.* 18:10573–10584.
72. de Kruijff, B. 1997. Lipid Polymorphism and Biomembrane Function. *Curr. Opin. Chem. Biol.* 1:564–569.
73. de Kruijff, B. 1997. Lipids beyond the Bilayer. *Nature.* 386:129.
74. van den Brink-van der Laan, E., J. A. Killian, and B. de Kruijff. 2004. Nonbilayer Lipids Affect Peripheral and Integral Membrane Proteins via Changes in the Lateral Pressure Profile. *Biochim. Biophys. Acta Biomembr.* 1666:275–288.
75. Liu, J., and J. C. Conboy. 2005. 1, 2-Diacyl-Phosphatidylcholine Flip-Flop Measured Directly by Sum-Frequency Vibrational Spectroscopy. *Biophys. J.* 89:2522–2532.
76. Pomorski, T. G., and A. K. Menon. 2016. Lipid Somersaults: Uncovering the Mechanisms of Protein-Mediated Lipid Flipping. *Prog. Lipid Res.* 64:69–84.
77. Nakano, M., M. Fukuda, …, T. Handa. 2009. Flip-Flop of Phospholipids in Vesicles: Kinetic Analysis with Time-Resolved Small-Angle Neutron Scattering. *J. Phys. Chem. B.* 113:6745–6748.
78. Kornberg, R. D., and H. M. McConnell. 1971. Inside-Outside Transitions of Phospholipids in Vesicle Membranes. *Biochemistry.* 10:1111–1120.
79. Rice, A., J. Zimmerberg, and R. W. Pastor. 2023. Initiation and Evolution of Pores Formed by Influenza Fusion Peptides Probed by Lysolipid Inclusion. *Biophys. J.* 122:1018–1032. https://doi.org/10.1016/j.bpj.2022.12.029.
80. Seu, K. J., L. R. Cambrea, …, J. S. Hovis. 2006. Influence of Lipid Chemistry on Membrane Fluidity: Tail and Headgroup Interactions. *Biophys. J.* 91:3727–3735.
81. Shinoda, W., and S. Okazaki. 1998. A Voronoi Analysis of Lipid Area Fluctuation in a Bilayer. *J. Chem. Phys.* 109:1517–1521.
82. Voronoi, G. 1908. Nouvelles Applications Des Paramètres Continus à La Théorie Des Formes Quadratiques. Deuxième Mémoire. Recherches Sur Les Parallélloèdres Primitifs. *J. für die Reine Angewandte Math. (Crelle's J.).* 1908:198–287.
83. Tarek, M. 2005. Membrane Electroporation: A Molecular Dynamics Simulation. *Biophys. J.* 88:4045–4053.
84. Piggot, T. J., D. A. Holdbrook, and S. Khalid. 2011. Electroporation of the E. Coli and S. Aureus Membranes: Molecular Dynamics Simulations of Complex Bacterial Membranes. *J. Phys. Chem. B.* 115:13381–13388.
85. Tieleman, D. P., H. Leontiadou, …, S.-J. Marrink. 2003. Simulation of Pore Formation in Lipid Bilayers by Mechanical Stress and Electric Fields. *J. Am. Chem. Soc.* 125:6382–6383.
86. Tieleman, D. P. 2004. The Molecular Basis of Electroporation. *BMC Biochem.* 5:10–12.
87. Vernier, P. T., and M. J. Ziegler. 2007. Nanosecond Field Alignment of Head Group and Water Dipoles in Electroporating Phospholipid Bilayers. *J. Phys. Chem. B.* 111:12993–12996.
88. Levine, Z. A., Y.-H. Wu, …, P. T. Vernier. 2009. Electroporation Sensitivity of Oxidized Phospholipid Bilayers. *Biophys. J.* 96:41a.
89. Vernier, P. T., M. J. Ziegler, …, D. P. Tieleman. 2006. Nanopore Formation and Phosphatidylserine Externalization in a Phospholipid Bilayer at High Transmembrane Potential. *J. Am. Chem. Soc.* 128:6288–6289.
90. van Zutphen, H., and L. L. M. Van Deenen. 1967. The Effect of Lysolecithin on the Electrical Resistance of Lecithin Bilayer Membranes. *Chem. Phys. Lipids.* 1:389–391.
91. Böckmann, R. A., B. L. De Groot, …, H. Grubmüller. 2008. Kinetics, Statistics, and Energetics of Lipid Membrane Electroporation Studied by Molecular Dynamics Simulations. *Biophys. J.* 95:1837–1850. https://doi.org/10.1529/biophysj.108.129437.
92. Casciola, M., D. Bonhenry, …, M. Tarek. 2014. A Molecular Dynamic Study of Cholesterol Rich Lipid Membranes: Comparison of Electroporation Protocols. *Bioelectrochemistry.* 100:11–17.
93. Cao, X., C. H. A. van de Lest, …, M. M. S. M. Wösten. 2022. Campylobacter Jejuni Permeabilizes the Host Cell Membrane by Short Chain Lysophosphatidylethanolamines. *Gut Microbes.* 14:2091371.
94. Rice, A., A. C. Zourou, …, M. L. Cotten. 2025. Investigating How Lysophosphatidylcholine and Lysophosphatidylethanolamine Enhance the Membrane Permeabilization Efficacy of Host Defense Peptide Piscidin 1. *J. Phys. Chem. B.* 129:210–227. https://doi.org/10.1021/acs.jpcb.4c05845.
95. Yoo, J., and Q. Cui. 2009. Curvature Generation and Pressure Profile Modulation in Membrane by Lysolipids: Insights from Coarse-Grained Simulations. *Biophys. J.* 97:2267–2276. https://doi.org/10.1016/j.bpj.2009.07.051.
96. Abouelhadid, S., S. J. North, …, B. W. Wren. 2019. Quantitative Analyses Reveal Novel Roles for N-Glycosylation in a Major Enteric Bacterial Pathogen. *mBio.* 10:e00297-19. https://doi.org/10.1128/mBio.00297-19.
97. Abouelhadid, S., J. Raynes, …, B. W. Wren. 2020. Characterization of Posttranslationally Modified Multidrug Efflux Pumps Reveals an Unexpected Link between Glycosylation and Antimicrobial Resistance. *mBio.* 11:e02604-20. https://doi.org/10.1128/mBio.02604-20.
98. Brandner, A. F., I. P. S. Smith, …, S. Khalid. 2025. Systematic Approach to Parametrization of Disaccharides for the Martini 3 Coarse-Grained Force Field. *J. Chem. Inf. Model.* 65:1537–1548. https://doi.org/10.1021/acs.jcim.4c01874.

Biophysical Journal, Volume *124*

Supplemental information

Atomistic modeling of lysophospholipids from the *Campylobacter jejuni* lipidome

Astrid F. Brandner, Kahlan E. Newman, Jonathan W. Essex, and Syma Khalid

Supplemental Material

Atomistic modelling of lysophospholipids from the *Campylobacter jejuni* lipidome

Kahlan E. Newman,[†,&] Astrid F. Brandner,[‡,&] Jonathan W. Essex,[†] and Syma Khalid[†,¶,*]

†*School of Chemistry, University of Southampton, Southampton, UK*

‡*Department of Biochemistry, University of Oxford, Oxford, UK*

¶*Department of Biochemistry, University of Oxford, Oxford, UK*

*E-mail: syma.khalid@bioch.ox.ac.uk

[&]These authors contributed equally

Self-assembly Simulations, Excess Water

Table S1: System contents for self-assembly simulations in excess water

Molecule	Small Single LPL systems				Large Single LPL systems (Anisotropic)			
	$LysoPE_{(18:1)}$	$LysoPE_{(16:0)}$	$LysoPE_{(19:0c)}$	$LysoPG_{(18:1)}$	$LysoPE_{(18:1)}$	$LysoPE_{(16:0)}$	$LysoPE_{(19:0c)}$	$LysoPG_{(18:1)}$
$LysoPE_{(18:1)}$	140	0	0	0	500	0	0	0
$LysoPE_{(16:0)}$	0	140	0	0	0	500	0	0
$LysoPE_{(19:0c)}$	0	0	140	0	0	0	500	0
$LysoPG_{(18:1)}$	0	0	0	140	0	0	0	500
Water	7000	7000	7000	7000	31059	29373	31967	33002
K^+	10	10	10	150	40	40	40	500
CL^-	10	10	10	10	40	40	40	0
Initial box dimensions / nm	7.0 x 7.0 x 7.0				10.9 x 10.9 x 10.9			

Self-assembly Simulations, C_W=0.4

Table S2: System contents for self-assembly simulations at C_W = 0.4

Molecule	Small Single LPL systems				Large Single LPL systems (Anisotropic)			
	$LysoPE_{(18:1)}$	$LysoPE_{(16:0)}$	$LysoPE_{(19:0c)}$	$LysoPG_{(18:1)}$	$LysoPE_{(18:1)}$	$LysoPE_{(16:0)}$	$LysoPE_{(19:0c)}$	$LysoPG_{(18:1)}$
$LysoPE_{(18:1)}$	140	0	0	0	500	0	0	0
$LysoPE_{(16:0)}$	0	140	0	0	0	500	0	0
$LysoPE_{(19:0c)}$	0	0	140	0	0	0	500	0
$LysoPG_{(18:1)}$	0	0	0	140	0	0	0	500
Water	2512	2377	2585	2870	8874	8392	9134	9429
K^+	2	2	2	142	20	20	20	500
CL^-	2	2	2	2	20	20	20	0
Initial box dimensions / nm	7.0 x 7.0 x 7.0				10.9 x 10.9 x 10.9			

Self-assembly Simulations, C_W=0.1

Table S3: System contents for self-assembly simulations at C_W = 0.1

Molecule	Small Single LPL systems				Large Single LPL systems (Anisotropic)			
	**LysoPE$_{(18:1)}$**	**LysoPE$_{(16:0)}$**	**LysoPE$_{(19:0c)}$**	**LysoPG$_{(18:1)}$**	**LysoPE$_{(18:1)}$**	**LysoPE$_{(16:0)}$**	**LysoPE$_{(19:0c)}$**	**LysoPG$_{(18:1)}$**
**LysoPE$_{(18:1)}$**	140	0	0	0	500	0	0	0
**LysoPE$_{(16:0)}$**	0	140	0	0	0	500	0	0
**LysoPE$_{(19:0c)}$**	0	0	140	0	0	0	500	0
**LysoPG$_{(18:1)}$**	0	0	0	140	0	0	0	500
**Water**	419	396	431	478	1479	1399	1523	1572
**K^+**	1	1	1	141	5	5	5	500
**CL^-**	1	1	1	1	5	5	5	0
**Initial box dimensions / nm**	5.4 x 5.4 x 5.4				9.9 x 9.9 x 9.9			

Bilayer Self-assembly Simulations

Table S4: System contents for bilayer self-assembly simulations

Molecule	Small Single LPL systems			
	POPG	POPE	POPA	20 % LPL
LysoPE$_{(18:1)}$	0	0	0	7
LysoPE$_{(16:0)}$	0	0	0	7
LysoPE$_{(19:0c)}$	0	0	0	7
LysoPG$_{(18:1)}$	0	0	0	7
POPG	140	0	0	63
POPE	0	140	0	42
POPA	0	0	140	7
Water	7000	7000	7000	7000
K^+	10	10	150	87
CL^-	10	10	10	10
Initial box dimensions / nm	7.2 x 7.2 x 7.2			

Equilibrium Bilayer Simulations

Table S5: System contents for equilibrium simulation of bilayers with and without lysophospholipids

Molecule	20% LPL			Phospholipids Only		
	R1	R2	R3	R1	R2	R3
LysoPE$_{(18:1)}$	63	63	63	0	0	0
LysoPE$_{(16:0)}$	63	63	63	0	0	0
LysoPE$_{(19:0c)}$	63	63	63	0	0	0
LysoPG$_{(18:1)}$	63	63	63	0	0	0
POPG	567	567	567	828	828	828
POPE	376	376	376	552	552	552
POPA	63	63	63	92	92	92
Water	103808	104583	104663	71713	71691	71756
K^+	1145	1147	1147	1113	1114	1113
CL^-	452	454	454	193	194	193
Initial box dimensions / nm	7.2 x 7.2 x 7.2			21.1 x 21.1 x 8.9	21.0 x 21.0 x 9.0	21.1 x 21.1 x 8.9

Equilibrium Bilayer Simulation Details

Table S6: Restraints for lipid phosphorus atoms and dihedrals; timestep (dt); and duration for equilibration stages

Equilibration stage	Position restraint kJ mol $^{-1}$nm^{-2}		**dt** / fs	**Length /** ps
	**Dihedrals**	**Lipid P**		
**NVT1**	1000	1000	1	125
**NVT2**	400	400	1	125
**NPT1**	400	200	2	500
**NPT2**	200	200	2	500
**NPT3**	40	100	2	500
**NPT4**	-	-	2	500

Native protein embedded in Mixed Bilayer

Table S7: System contents for native protein in mixed bilayer. PglB protein was glycosylated in Asn534 (more details in main Method section). Pept refers to the acceptor sequon peptide; LLO refers to the glycosylated lipid donor.

**Molecule**	**20% LPL R1-R3**
**$LysoPE_{(18:1)}$**	22
**$LysoPE_{(16:0)}$**	24
**$LysoPE_{(19:0c)}$**	23
**$LysoPG_{(18:1)}$**	24
**POPG**	211
**POPE**	114
**POPA**	21
**LLO**	1
**PglB**	1
**Pept**	1
**MG^{2+}**	2
**Water**	61969
**K^{+}**	420
**CL^{-}**	169
**Initial box dimensions / nm**	12.6x12.6x15.8

Electroporation Simulations

Table S8: System contents for electric field simulation of bilayers with and without lysophospholipids

Molecule	20% LPL			Phospholipids Only		
	R1	R2	R3	R1	R2	R3
**LysoPE$_{(18:1)}$**	28	28	28	0	0	0
**LysoPE$_{(16:0)}$**	28	28	28	0	0	0
**LysoPE$_{(19:0c)}$**	28	28	28	0	0	0
**LysoPG$_{(18:1)}$**	28	28	28	0	0	0
**POPG**	252	252	252	324	324	324
**POPE**	168	168	168	216	216	216
**POPA**	28	28	28	36	36	36
**Water**	40150	40122	40194	40320	40323	40323
**K^+**	489	490	492	457	458	458
**CL^-**	181	182	184	97	98	98
**Initial box dimensions / nm**	13.0x13.0x10.9	13.1x13.1x10.8	12.9x12.9x11.1	13.5x13.5x10.4	13.5x13.5x10.4	13.5x13.5x10.5

Simulation box collapse

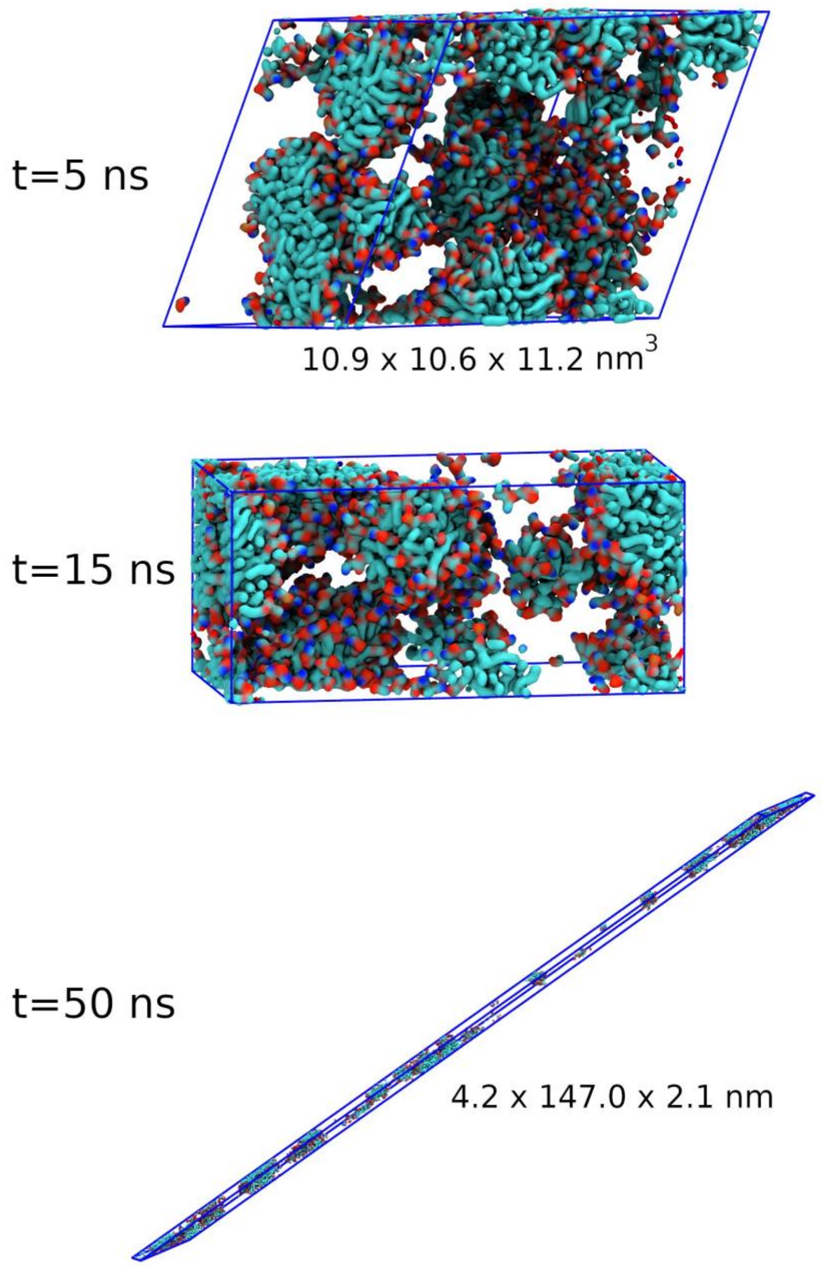

Figure S1: Collapse of a simulation box under anisotropic pressure coupling. Lipids shown as surfaces, coloured by element. Unit cell shown by blue lines. Simulation of lysoPE$_{(18:1)}$ (500 lipids, C_W=0.7) initially forms micelles. The unit cell deforms as the simulation progresses, resulting in box dimensions less than twice the electrostatics cut-off. The simulation crashes shortly after 50 ns.

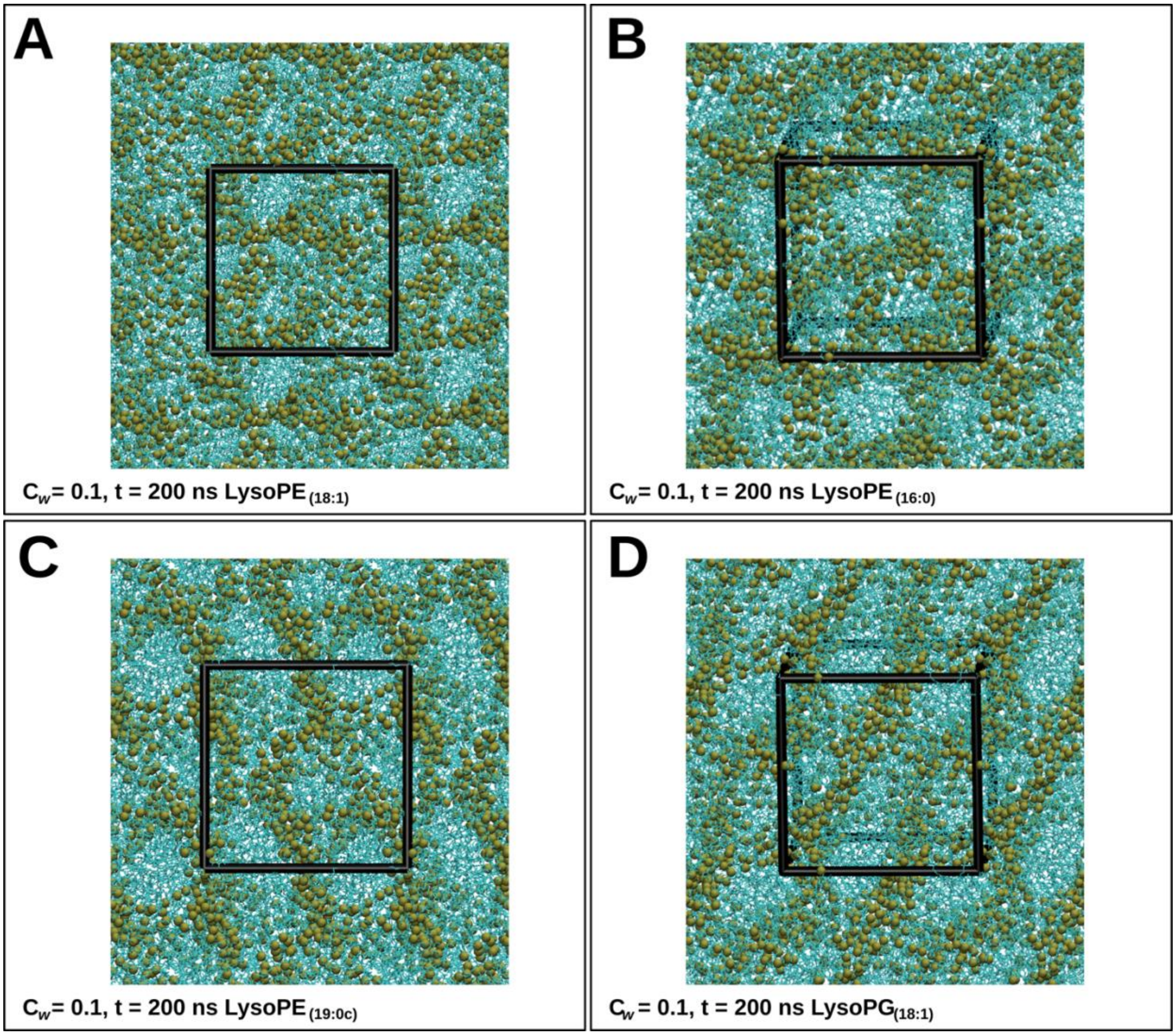

Figure S2: Final snapshots of the isotropic single LPL self-assembly of larger boxes at Cw= 0.1 (most concentrated systems). **(A)** LysoPE$_{(18:1)}$, **(B)** LysoPE$_{(16:0)}$, **(C)** LysoPE, **(D)** LysoPG(18:1). The simulated box is shown in black, and replicated in space to aid the visualisation of the aggregates.

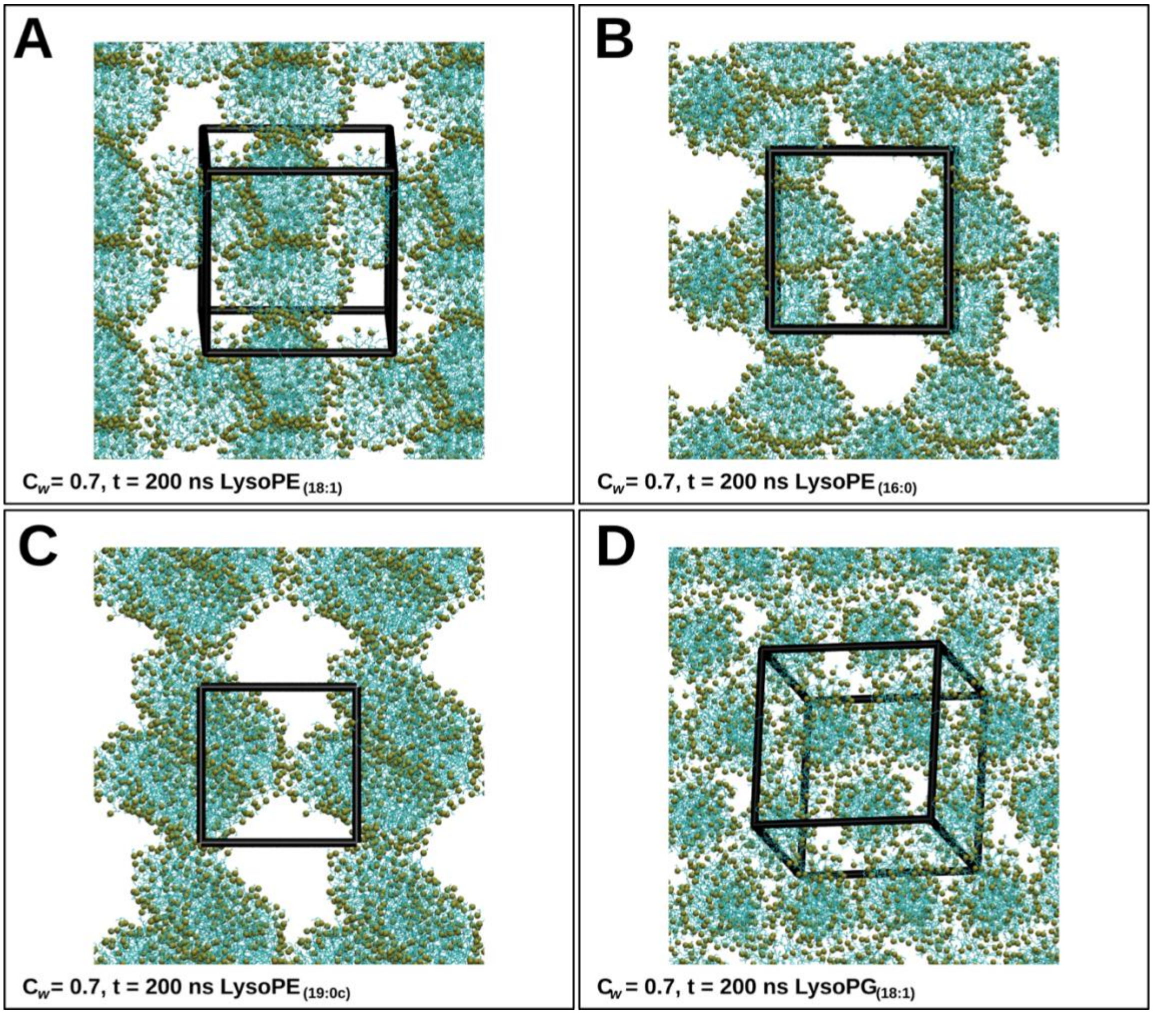

Figure S3: Final snapshots of the isotropic single LPL self-assembly of larger boxes at Cw= 0.7 (most diluted systems). **(A)** LysoPE$_{(18:1)}$, **(B)** LysoPE$_{(16:0)}$, **(C)** LysoPE$_{(19:0c)}$, **(D)** LysoPG(18:1). The simulated box is shown in black, and replicated in space to aid the visualisation of the aggregates.

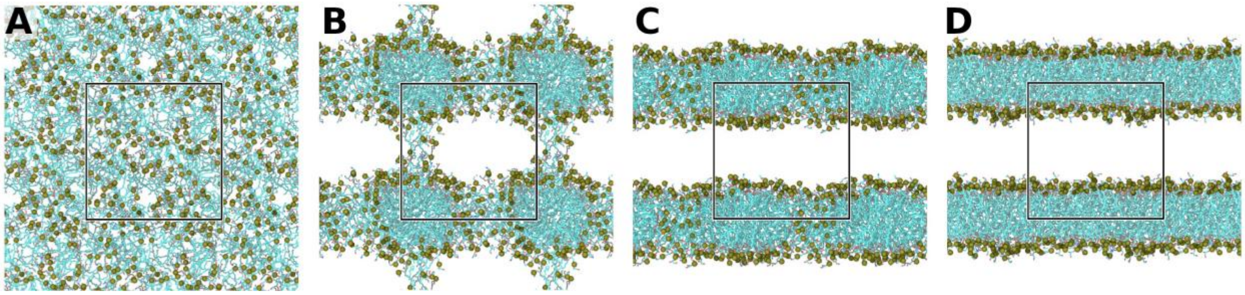

Figure S4: Stages of bilayer self-assembly as described by Skjevik et al. [71]. Snapshots taken from self-assembly simulation of 20% LPL-phospholipid mixture. Lipids shown in stick representation coloured by element. Phosphorus atoms of the lipid headgroups highlighted as tan spheres. Water and ions omitted for clarity. Unit cell indicated by a black box. **(A)** Initial lipid solution. **(B)** Micelle-like assembly with lipid bridges between micelles and their periodic images. **(C)** Porated bilayer with lipid headgroups within the hydrophobic core region. **(D)** Non-porous bilayer formed.

Depletion-enrichment indices

Table S9: Depletion-enrichment indices (DEI) for lipids in the mixed bilayers. Values are presented as the mean ± standard deviation over the second half (final 500 ns) of each simulation. Calculated using the Python package LiPyphilic[41].

**Replica**	**Molecule**	**DEI**						
		**POPG**	**POPE**	**POPA**	**lysoPE**$_{(18:1)}$	**lysoPE**$_{(16:0)}$	**lysoPE**$_{(19:0c)}$	**lysoPG**$_{(18:1)}$
**R1**	**POPG**	0.95 ± 0.02	1.00 ± 0.02	0.92 ± 0.05	0.96 ± 0.05	1.06 ± 0.04	0.97 ± 0.06	0.95 ± 0.05
	**POPE**	1.05 ± 0.02	0.97 ± 0.04	1.11 ± 0.09	1.02 ± 0.08	0.95 ± 0.07	1.02 ± 0.07	1.10 ± 0.06
	**POPA**	0.96 ± 0.05	1.11 ± 0.10	0.9 ± 0.26	1.17 ± 0.28	0.90 ± 0.21	1.17 ± 0.22	0.93 ± 0.21
	**lysoPE**$_{(18:1)}$	1.02 ± 0.06	1.03 ± 0.09	1.19 ± 0.28	0.92 ± 0.21	1.18 ± 0.19	0.98 ± 0.19	1.11 ± 0.20
	**lysoPE**$_{(16:0)}$	1.12 ± 0.05	0.95 ± 0.07	0.90 ± 0.21	1.16 ± 0.20	0.8 ± 0.26	0.97 ± 0.22	0.93 ± 0.20
	**lysoPE**$_{(19:0c)}$	1.04 ± 0.06	1.04 ± 0.08	1.20 ± 0.23	0.99 ± 0.19	0.99 ± 0.23	1.09 ± 0.31	0.99 ± 0.17
	**lysoPG**$_{(18:1)}$	0.95 ± 0.05	1.04 ± 0.07	0.88 ± 0.20	1.04 ± 0.19	0.89 ± 0.20	0.93 ± 0.17	0.93 ± 0.28
**R2**	**POPG**	0.96 ± 0.02	0.99 ± 0.02	0.9 ± 0.05	0.98 ± 0.05	1.03 ± 0.06	0.97 ± 0.06	0.92 ± 0.05
	**POPE**	1.05 ± 0.02	0.98 ± 0.04	1.11 ± 0.06	1.01 ± 0.06	0.98 ± 0.08	1.00 ± 0.06	1.10 ± 0.07
	**POPA**	0.95 ± 0.05	1.11 ± 0.07	1.07 ± 0.39	1.08 ± 0.19	1.16 ± 0.25	1.07 ± 0.19	0.84 ± 0.24
	**lysoPE**$_{(18:1)}$	1.04 ± 0.05	1.02 ± 0.07	1.09 ± 0.187	0.83 ± 0.2	0.97 ± 0.21	1.15 ± 0.19	1.16 ± 0.26
	**lysoPE**$_{(16:0)}$	1.08 ± 0.06	0.97 ± 0.08	1.16 ± 0.24	0.96 ± 0.20	0.75 ± 0.25	0.95 ± 0.23	1.11 ± 0.19
	**lysoPE**$_{(19:0c)}$	1.04 ± 0.06	1.02 ± 0.07	1.09 ± 0.20	1.16 ± 0.19	0.96 ± 0.23	1.14 ± 0.26	1.06 ± 0.19
	**lysoPG**$_{(18:1)}$	0.93 ± 0.05	1.04 ± 0.07	0.80 ± 0.23	1.10 ± 0.25	1.06 ± 0.18	0.99 ± 0.17	0.95 ± 0.30
**R3**	**POPG**	0.95 ± 0.01	1.00 ± 0.02	0.9 ± 0.05	0.98 ± 0.05	1.01 ± 0.06	1.00 ± 0.05	0.95 ± 0.05
	**POPE**	1.06 ± 0.02	0.97 ± 0.04	1.13 ± 0.07	1.02 ± 0.07	0.95 ± 0.07	1.01 ± 0.06	1.05 ± 0.06
	**POPA**	0.95 ± 0.05	1.13 ± 0.08	0.86 ± 0.24	1.23 ± 0.22	1.08 ± 0.23	0.94 ± 0.17	1.04 ± 0.20
	**lysoPE**$_{(18:1)}$	1.04 ± 0.05	1.03 ± 0.08	1.24 ± 0.21	1.01 ± 0.32	0.99 ± 0.19	0.88 ± 0.22	1.06 ± 0.21
	**lysoPE**$_{(16:0)}$	1.07 ± 0.06	0.95 ± 0.08	1.09 ± 0.24	0.98 ± 0.18	1.04 ± 0.30	0.98 ± 0.22	1.14 ± 0.20
	**lysoPE**$_{(19:0c)}$	1.07 ± 0.06	1.02 ± 0.07	0.95 ± 0.17	0.88 ± 0.22	0.99 ± 0.22	1.01 ± 0.24	1.18 ± 0.18
	**lysoPG**$_{(18:1)}$	0.95 ± 0.05	1.0 ± 0.06	0.98 ± 0.19	1.0 ± 0.20	1.08 ± 0.19	1.1 ± 0.18	0.71 ± 0.30

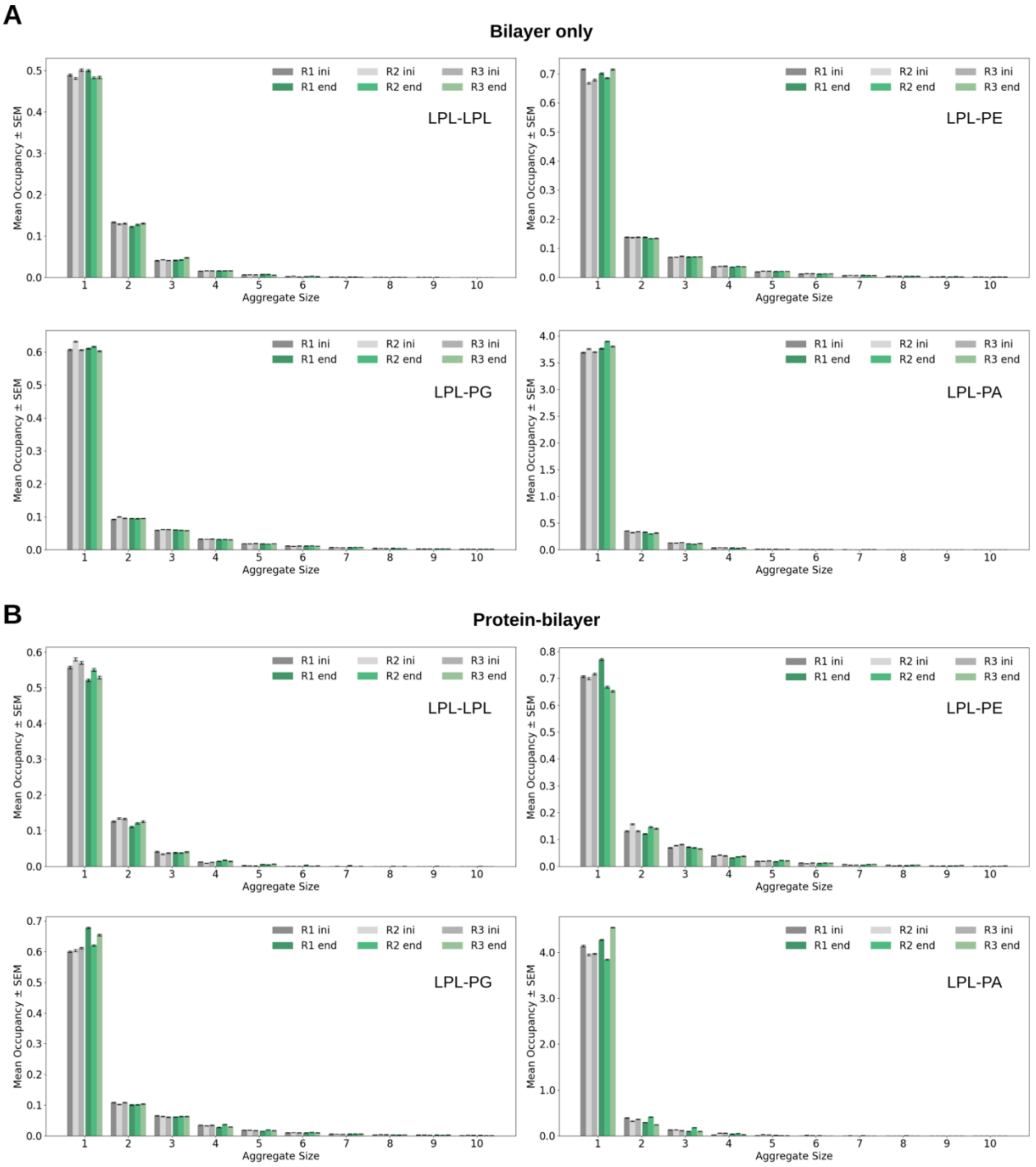

Figure S5: Aggregation propensity of LPLs with different lipid types. The frequency of observed aggregates was computed for each replica separately for the first and last 200 ns of the production run. Values are normalised by the total number of molecules of the second lipid species in the plot label. Plots are separated by phospholipid types in contact with LPL: POPA (LPL-PA), POPG (LPL-PG) and POPE (LPL-PE), or lysophopholipids (LPL-LPL). For clarity only aggregates up to size 10 are shown. Aggregates larger than 10 (if existed at all) were insignificant.

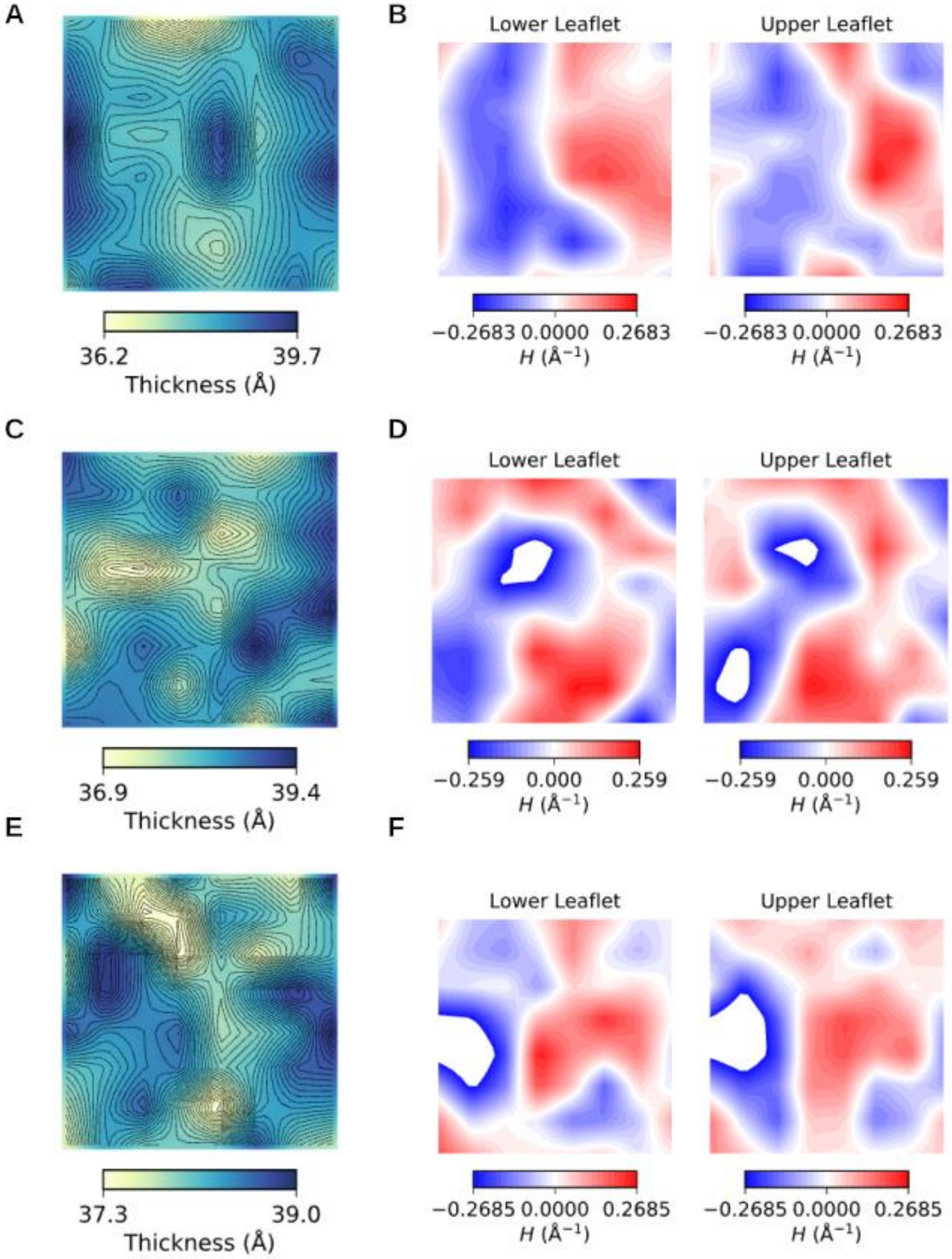

Figure S6: Mean Thickness and Mean Curvature for all 3 replicas (R1-R3) of 20% LPL bilayer systems.The last 100 ns of the production run were used to compute the average thickness and the mean curvature. (A,C,E) Average local thickness for R1, R2, and R3 respectively, projected on to the bilayer plane. (B,D,F) Plots of mean curvature for the lower and upper leaflet of the studied systems

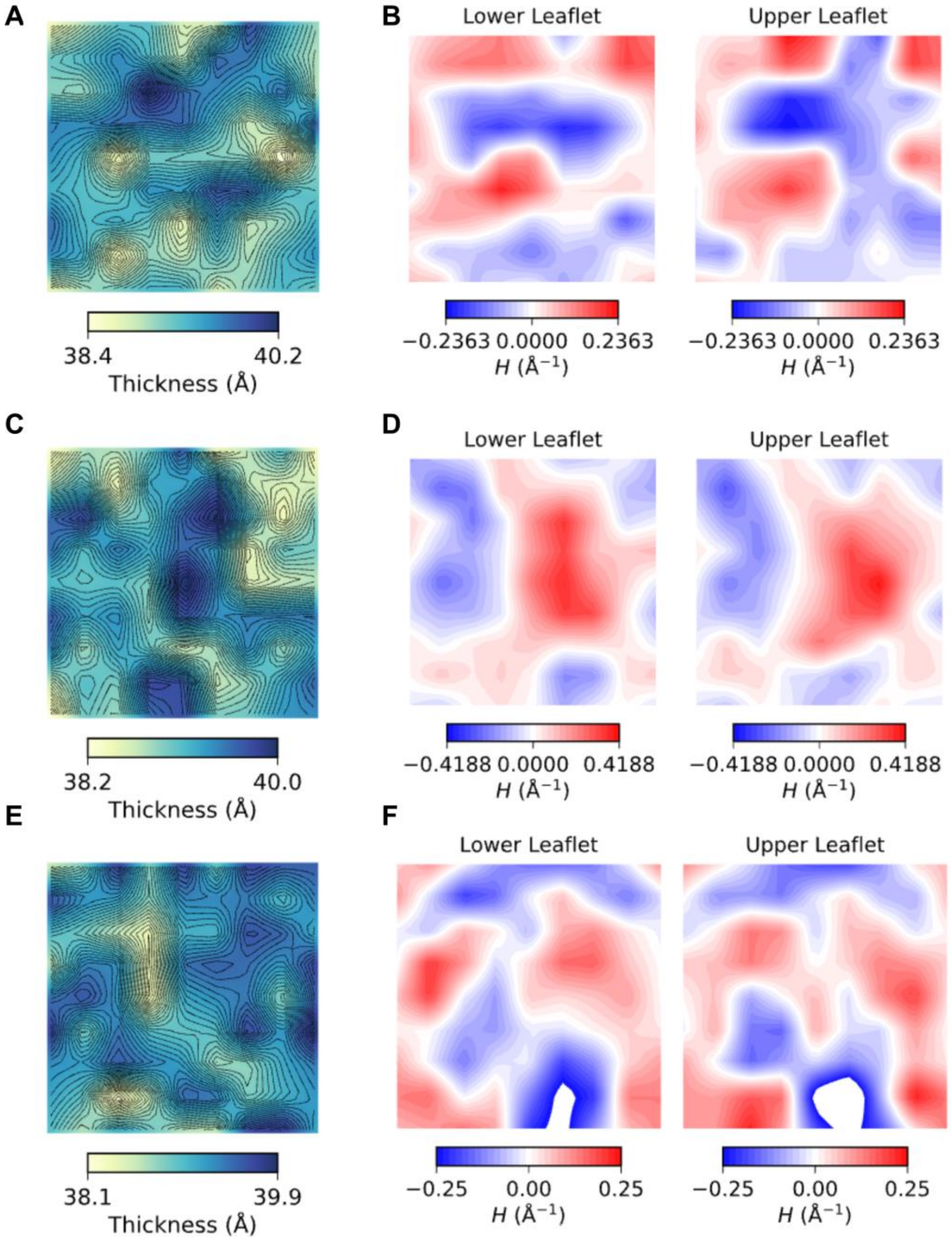

Figure S7: Mean Thickness and Mean Curvature for all 3 replicas (R1-R3) of the bilayers containing only POPA, POPG, POPE. The last 100 ns of the production run were used to compute the average thickness and the mean curvature. (A,C,E) Average local thickness for R1, R2, and R3 respectively, projected on to the bilayer plane. (B,D,F) Plots of mean curvature for the lower and upper leaflet of the studied systems

Table S10: Area compressibility moduli for bilayer systems. A block analysis was performed for the last 500 ns of the respective trajectories. Blocks of 100 ns were used, the means for each replica are reported below with their associated standard errors in brackets.

	**K_A (mN/m)**	
	**Phospholipids only**	**20 % LPL**
**R1**	201 (16)	200 (2)
**R2**	223 (13)	210 (26)
**R3**	202 (6)	186 (12)

Membrane Protein

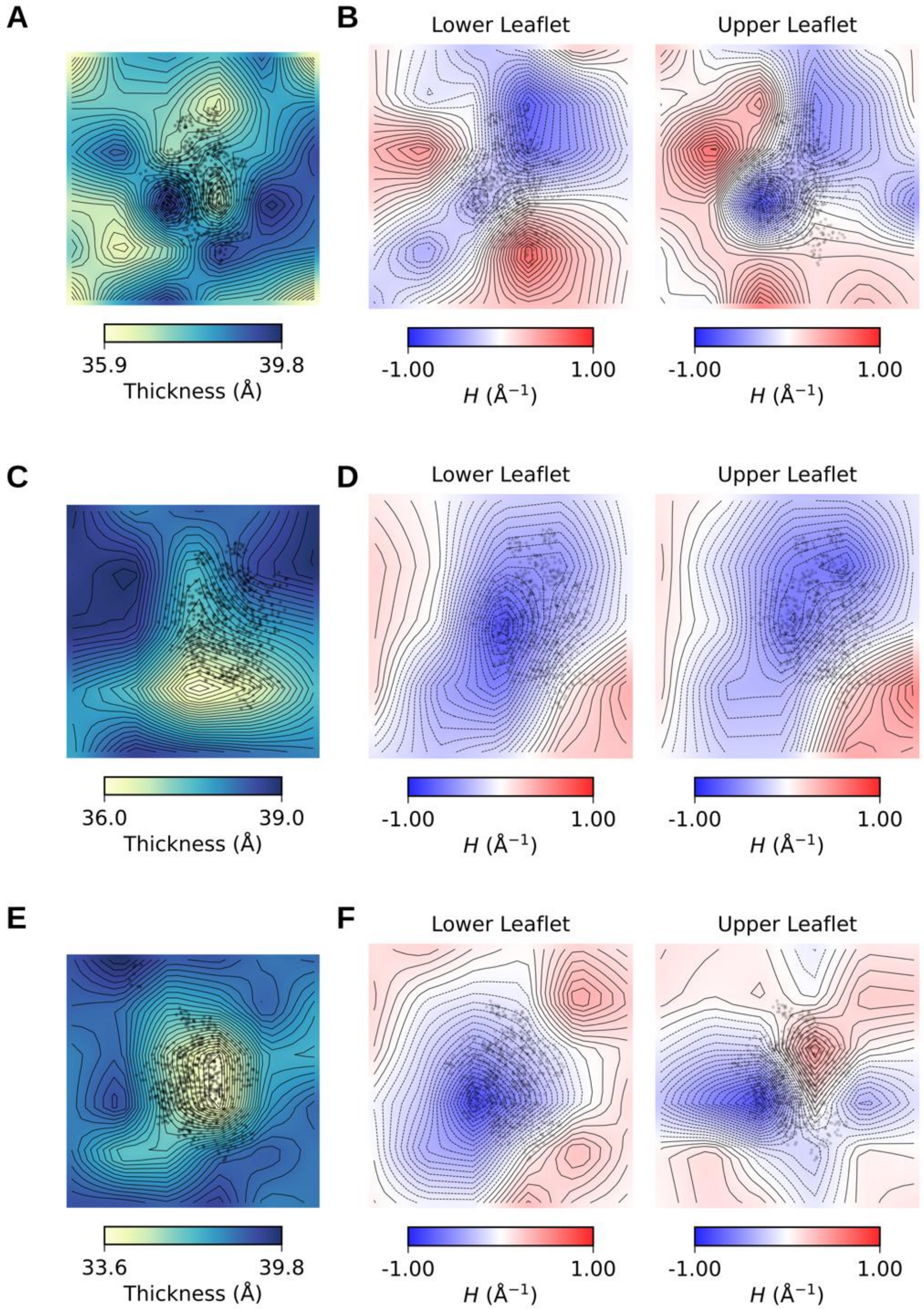

Figure S8: Mean Thickness and Mean Curvature for all 3 replicas (R1-R3) of Pglb-Bilayer systems.The last 100 ns of the production run were used to compute the average thickness and the mean curvature. (A,C,E) Average local thickness for R1, R2, and R3 respectively, projected on to the bilayer plane. (B,D,F) Plots of mean curvature for the lower and upper leaflet of the studied systems. In all plots the average protein positions are also plotted as black transparent dots for reference.

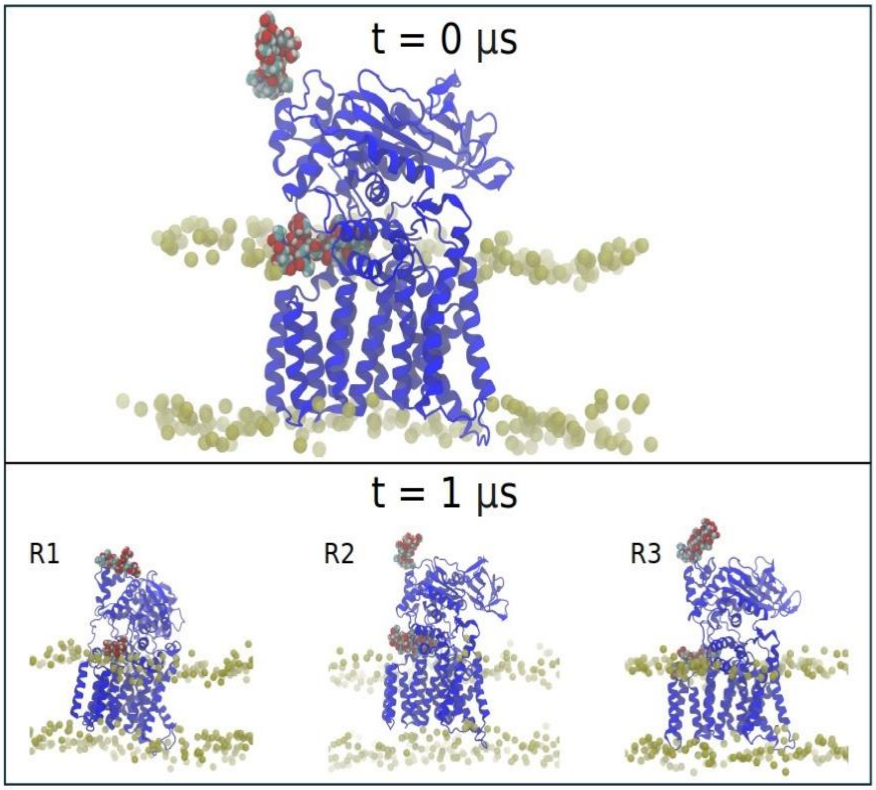

Figure S9: Zoomed-in sideviews of Initial and final snapshots of PglB/mixed bilayer for each of the replicas (R1-R3). PglB is coloured in blue cartoon representation, the glycans are depicted as coloured spheres and lipid phosphorus atoms are depicted as brown spheres.

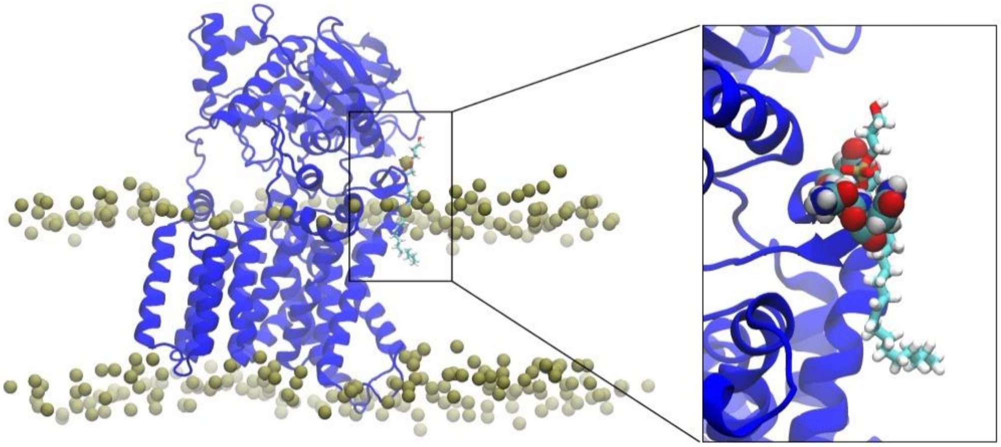

Figure S10: Final snapshot of R1 replica of PglB/mixed bilayer highlighting LysoPG$_{(18:1)}$ lipid (sticks) is located outside the bilayer alongside PglB (blue cartoon representation). Asparagine residues forming a 'cage' of interactions with the extracted LysoPG$_{(18:1)}$ are shown as spheres in the zoomed-in box. Lipid phosphorus atoms are depicted as brown spheres. Some phosphorus atoms in the immediate plane of vision in front of the protein have been omitted for clarity.

Table S11: Average Sugar-lipid contacts over the whole trajectories

Molecule	Average Sugar-lipid contacts		
	R1	R2	R3
**POPG**	0.416 ± 0.008	0.736 ± 0.011	1.021 ± 0.011
**POPE**	1.591 ± 0.014	0.386 ± 0.009	1.512 ± 0.013
**POPA**	0 ± 0	0.159 ± 0.005	0.266 ± 0.006
**LysoPE**$_{(18:1)}$	0.0204 ± 0.002	0.137 ± 0.005	0.184 ± 0.006
**LysoPE**$_{(16:0)}$	0 ± 0	0.042 ± 0.003	0 ± 0
**LysoPE**$_{(19:0c)}$	0.002 ± 0.001	0.091 ± 0.004	0.014 ± 0.002
**LysoPG**$_{(18:1)}$	0.019 ± 0.002	0.214 ± 0.006	0.021 ± 0.002

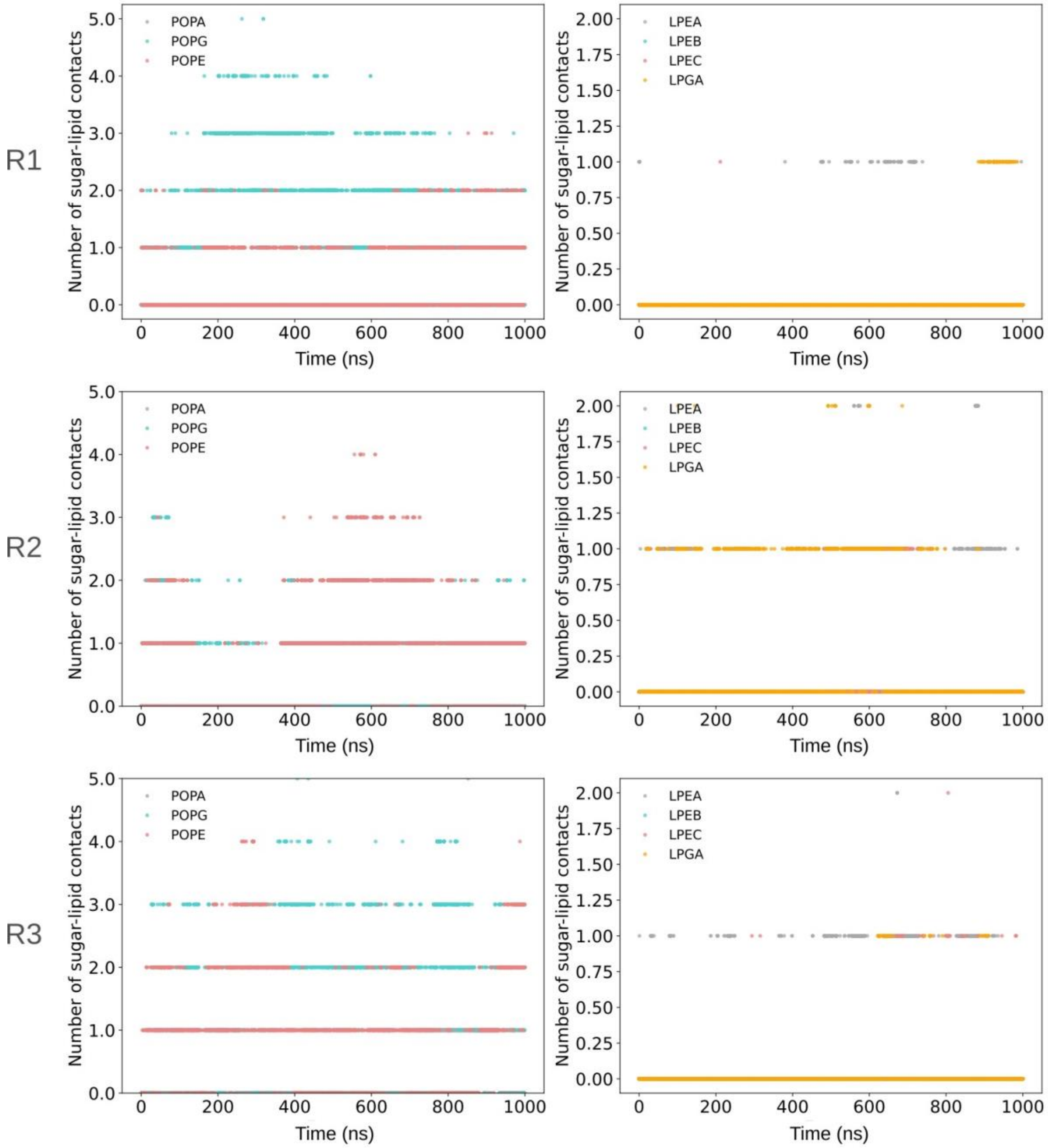

Figure S11 Contacts were computed between the sugar moieties of the LLO donor lipid and any lipid atom of the corresponding lipid type. Plots are separated for either phospholipids: POPA, POPG and POPE, or lysophopholipids: LPEA: LysoPE$_{(18:1)}$, LPEB: LysoPE$_{(16:0)}$, LPEC: LysoPE(19:0*c*), LPGA: LysoPG(18:1)

Electroporation Simulations

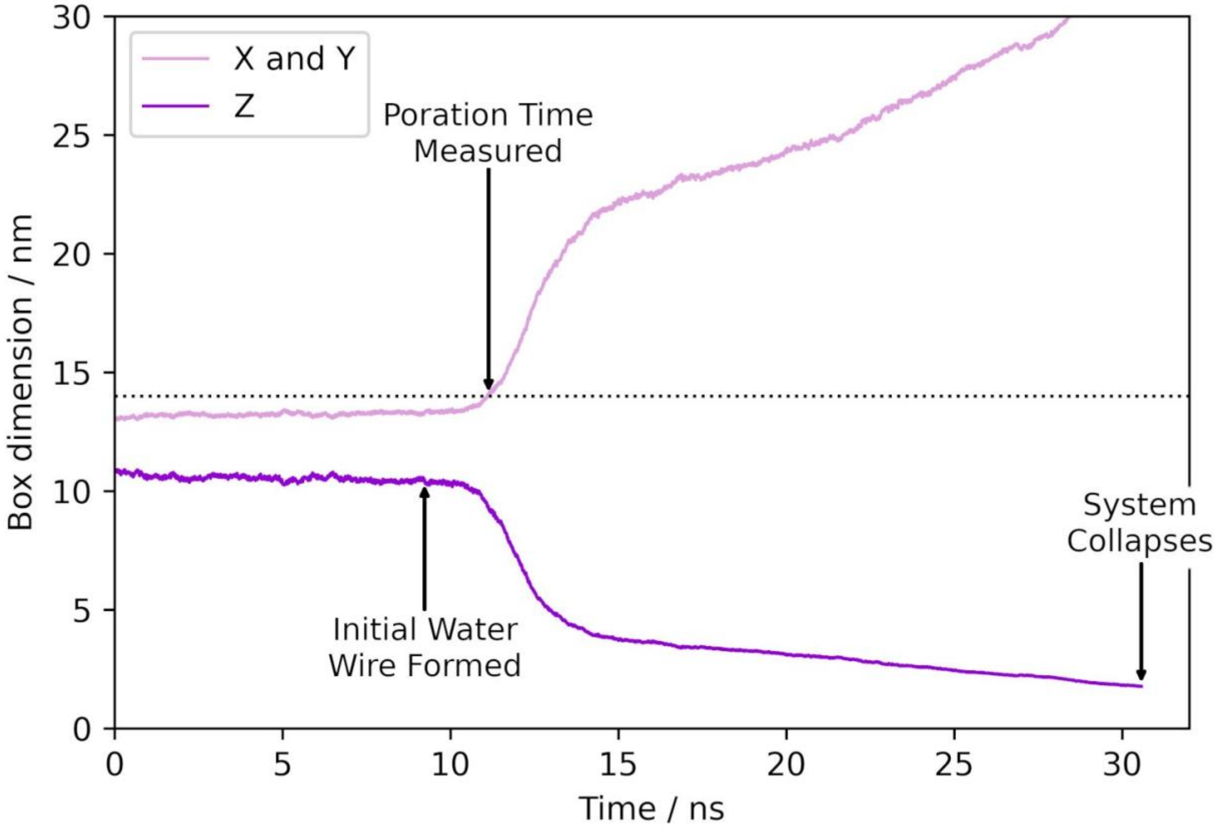

Figure S12: Box dimensions during electroporation of a mixed bilayer (R1, field strength 0.15 V nm [1]). The formation of the water wire has a negligible effect on the box dimensions, but as lipid headgroups move into the hydrophobic core to stabilise the water channel the bilayer expands in the *xy*-plane. The poration time was measured as the point at which the *x* (and *y*) box dimension increased to > 10% above the equilibrium box dimension; at this point, a substantial water channel has formed in the bilayer.

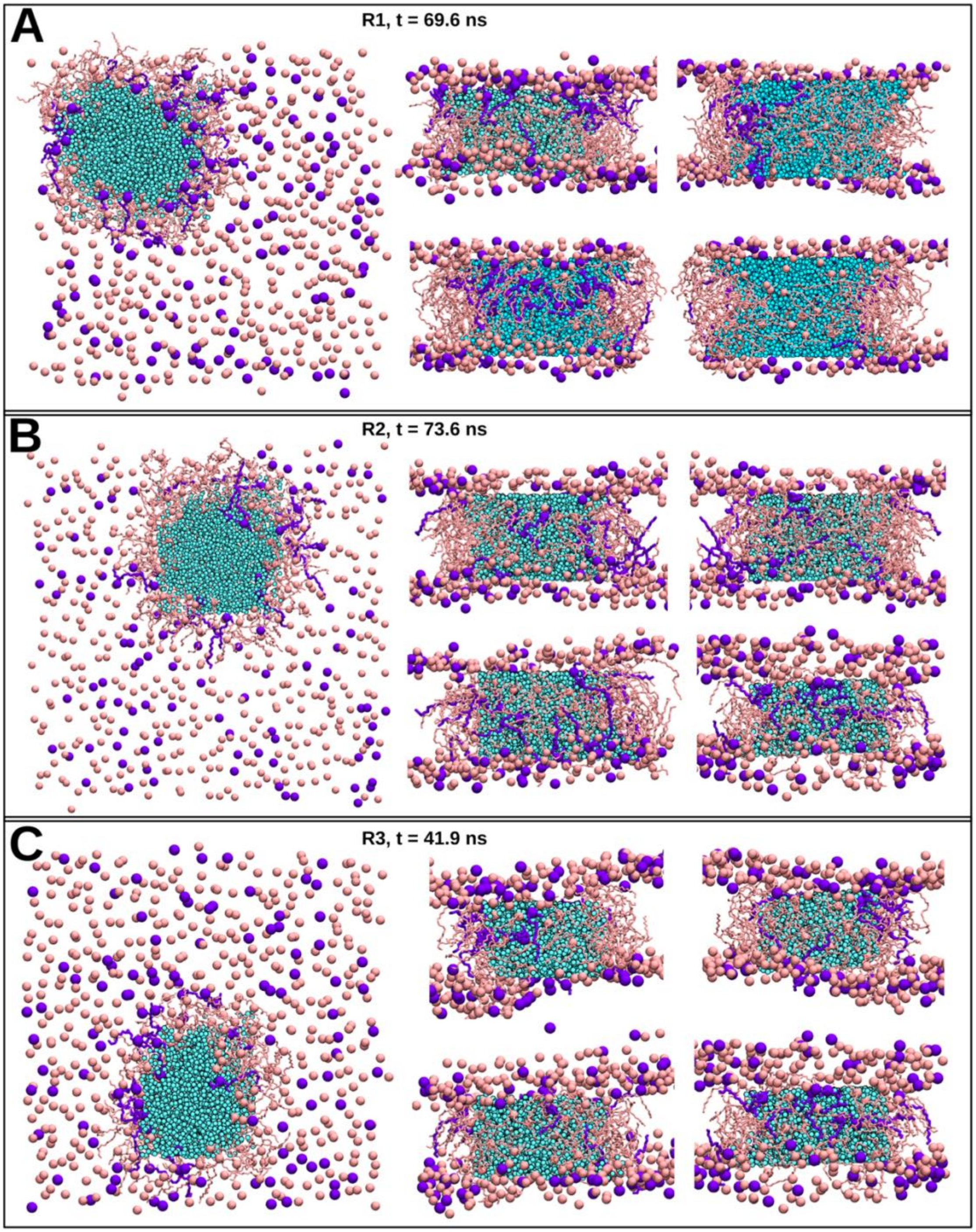

Figure S13: Simulation snapshots from the respective pore formation times reported for a field strength E= 0.175V nm^{-1}; the lowest studied strength that generated pores in all 3 replicas. (A,B,C) Top and side views for replicas R1, R2, and R3 respectively. The colouring scheme follows the same as in Fig.11: POP, POPG, POPA in pink; LPLs in purple; oxygen atoms from water inside the water pore are depicted as cyan spheres. Only the lipids in direct contact with the water in the pore are shown as sticks. All the phosphorus atoms from the lipids are shown as spheres.

Table S12: LPL ratio from lipids in contact with waters forming the initial water wire or the reported pore.

Replica	E= 0.15 Vnm^{-1}				E= 0.175 Vnm-1			
	**water wire**	**# LPL; # PL**	**pore**	**# LPL; # PL**	**water wire**	**# LPL; # PL**	**pore**	**# LPL; # PL**
**R1**	0.25	5; 15	0.23	18; 61	0.25	5; 15	0.21	20; 75
**R2**	-				0.16	5; 27	0.20	15; 63
**R3**	0.23	6; 20	0.26	22; 61	0.04	1; 24	0.19	12; 50
